# Fungi inhabiting attine ant colonies: reassessment of the genus *Escovopsis* and description of *Luteomyces* and *Sympodiorosea* gens. nov.

**DOI:** 10.1186/s43008-021-00078-8

**Published:** 2021-08-24

**Authors:** Quimi Vidaurre Montoya, Maria Jesus Sutta Martiarena, Rodolfo Bizarria Jr., Nicole Marie Gerardo, Andre Rodrigues

**Affiliations:** 1 Department of General and Applied Biology, São Paulo State University (UNESP), Avenida 24-A, n. 1515, Bela Vista, Rio Claro, SP 13.506-900 Brazil; 2 Center for the Study of Social Insects, São Paulo State University (UNESP), Rio Claro, SP Brazil; 3 Department of Biology, O. Wayne Rollins Research Center, Emory University, Atlanta, USA

**Keywords:** Attina, Evolution, Fungus-growing ants, *Hypocreaceae*, Symbiosis, Systematics, Four new taxa

## Abstract

*Escovopsis* is a diverse group of fungi, which are considered specialized parasites of the fungal cultivars of fungus-growing ants. The lack of a suitable taxonomic framework and phylogenetic inconsistencies have long hampered *Escovopsis* research. The aim of this study is to reassess the genus *Escovopsis* using a taxonomic approach and a comprehensive multilocus phylogenetic analysis, in order to set the basis of the genus systematics and the stage for future *Escovopsis* research. Our results support the separation of *Escovopsis* into three distinct genera. In light of this, we redefine *Escovopsis* as a monophyletic clade whose main feature is to form terminal vesicles on conidiophores. Consequently, *E. kreiselii* and *E. trichodermoides* were recombined into two new genera, *Sympodiorosea* and *Luteomyces*, as *S*. *kreiselii* and *L*. *trichodermoides*, respectively. This study expands our understanding of the systematics of *Escovopsis* and related genera, thereby facilitating future research on the evolutionary history, taxonomic diversity, and ecological roles of these inhabitants of the attine ant colonies.

**Supplementary Information:**

The online version contains supplementary material available at 10.1186/s43008-021-00078-8.

## Introduction

The kingdom *Fungi* comprises organisms with wide morphological and genetic diversity (Mueller and Schmit 2007; Hawksworth and Lücking 2017). Through systematic approaches, taxonomists have developed mechanisms to categorize organisms based on their phenotypic and genetic characters (Komarek and Beutel 2006; Pavlinov 2018). Notwithstanding, taxonomic and phylogenetic incongruities like non-monophyly preclude the study of many fungal groups, as is the case for the genus *Escovopsis* (*Ascomycota*: *Hypocreales*, *Hypocreaceae*). *Escovopsis* is a diverse group of fungi, members of which are presumed to have evolved parasitizing the mutualistic fungus of fungus-growing ants (*Formicidae: Myrmicinae: Attini*: *Attina*, the “attines”) (Yek et al. 2012). Besides, *Escovopsis* has only been found associated with fungus-growing ant colonies, suggesting that the genus has evolved in relation to these ants’ system, potentially for millions of years. Despite its biological importance in relation to a canonical system for the study of coevolution and symbiosis, the paucity of taxonomic studies and unresolved phylogenetic inconsistencies have prevented a comprehensive understanding of the systematics, ecology, and evolution of these hypocrealean fungi.

More than a century has passed since Möller (1893) observed a group of “fungi with strong conidial shapes” (i.e. more prevalent fungi) in attine gardens, which 80 years later would be named *Phialocladus* (Kreisel 1972). Almost two decades later, *Phialocladus* was considered an invalid name because of the lack of the type specimen for its type species, *P. zsoltii*, and consequently, it was renamed as *Escovopsis* with *E. weberi* designated as the type species of the latter genus (Muchovej and Della Lucia 1990). Although the macroscopic characters of *E. weberi* were not fully described by the authors, the description of this species established the foundation for *Escovopsis* taxonomy. In 1995, Seifert et al. (1995) described *Escovopsis aspergilloides* in a detailed taxonomic study. However, after this study, the taxonomy of *Escovopsis* was set aside for 18 years.

Unlike systematics studies, the relationship between *Escovopsis* and the attine ants’ mutualistic fungi has been the topic of numerous studies (Currie et al. 2003; Gerardo et al. 2006a, b; Taerum et al. 2007; Folgarait et al. 2011; Elizondo Wallace et al. 2014; Marfetán et al. 2015; Birnbaum and Gerardo 2016; de Man et al. 2016; Heine et al. 2018). A great diversity was attributed to the genus *Escovopsis* through these studies, yet the morphology and phylogenetic placement of most of the strains named as *Escovopsis* were not properly assessed. The first ideas about the phylogenetic position of *Escovopsis* were gradually emerging (Currie et al. 2003; Gerardo et al. 2006b; Augustin et al. 2013; Masiulionis et al. 2015; Meirelles et al. 2015a, b). Initially, some authors suggested that *Escovopsis* belonged to the *Hypocreales*, although, no phylogenetic evidence was provided to support that hypothesis at the time (Currie et al. 1999a, b). The first phylogenetic analysis of *Escovopsis* confirmed the genus to be placed in the *Hypocreales*, as a sister clade of the *Hypocreaceae* (Currie et al. 2003). However, a more extensive phylogenetic analysis of *Escovopsis* strains associated with fungus gardens of *Apterostigma* ants indicated that the genus belonged to the *Hypocreaceae* (Gerardo et al. 2006b).

Augustin et al. (2013) were the first authors to combine morphological and phylogenetic approaches to study *Escovopsis*. Based on internal transcribed spacer (ITS) and large subunit ribosomal RNA (LSU) sequences, the authors described *Escovopsis lentecrescens*, *E. microspora*, and *E. moelleri*, which formed a monophyletic clade with *E*. *weberi* and *E*. *aspergilloides*. The most remarkable character of these newly named species was the presence of conidiophores with vesicles, as previously described by Muchovej and Della Lucia (1990) and Seifert et al. (1995). Nonetheless, while phylogenetic analyses of Augustin et al. (2013) based on ITS and LSU sequences suggested that *Escovopsis* formed a monophyletic clade, their analyses based on translation elongation factor 1-alpha (*tef*1) (including all strains treated as *Escovopsis* at that time except for the ex-type culture of *E. weberi*) suggested that the genus may not be monophyletic.

For almost 30 years, the genus *Escovopsis* was morphologically defined by the presence of conidiophores with vesicles that support the phialides (i.e., enteroblastic conidiogenous cells), from which conidia are produced. However, that changed with the introduction of *Escovopsis trichodermoides* (Masiulionis et al. 2015) and *E. kreiselii* (Meirelles et al. 2015a). These species have conidiophores without vesicles and with poorly differentiated conidiogenous cells (i.e., holoblastic determinate conidiogenous cells with synchronous arrangement; *E. trichodermoides*) and sympodial conidiogenous cells (i.e., holoblastic proliferous conidiogenous cells; *E. kreiselii*) instead of phialides. Therefore, Meirelles et al. (2015a) amended the morphological description of *Escovopsis* to insert the morphological features of *E. kreiselii*. However, because Masiulionis et al. (2015) and Meirelles et al. (2015a) were published at nearly the same time, the new definition did not include *E. trichodermoides.*

The insertion of *E. trichodermoides* and *E. kreiselii* within *Escovopsis* not only meant changes to the morphological circumscription of the genus but also intensified the phylogenetic uncertainties showed by Augustin et al. (2013). In the phylogenies produced by Masiulionis et al. (2015) and Meirelles et al. (2015a, b), it was clear that vesiculate *Escovopsis* were more closely related to *Escovopsioides* (Augustin et al. 2013) than to *E. trichodermoides* and *E. kreiselii*. Nonetheless, both Masiulionis et al. (2015) and Meirelles et al. (2015a, b) preferred to maintain *E. trichodermoides*, *E. kreiselii* and the vesiculate *Escovopsis* as placing in the same genus.

Recently, Montoya et al. (2019) used the ITS, LSU, and *tef*1 markers in a multilocus phylogenetic approach to describe *Escovopsis clavata* and *E. multiformis*. The authors noticed that disagreements in the *Escovopsis* taxonomy occurred among vesiculate *Escovopsis*, *E. trichodermoides* and *E. kreiselii*. Therefore, they highlighted the need to utilize new molecular markers to resolve the phylogeny of the genus. However, subsequent description of five new *Escovopsis* species (Marfetán et al. 2018) further complicated *Escovopsis* taxonomy because interpretation of the phylogenetic analyses made by Marfetán et al. (2018) had some limitations: (1) it was based on the LSU and *tef*1 genes separately; (2) the *tef*1 sequences obtained in the study do not align with those sequences in previously published studies; (3) some LSU sequences do not have similarity with *Escovopsis*; and (4) some of the new species (*Escovopsis atlas*, *E. catenulata*, and *E. pseudoweberi*) fall in the same clade, but strains of *E. atlas* fall in different (non-monophyletic) clades.

Given this complicated and piecemeal research history, the aim of this study is to reassess the genus *Escovopsis* by using a comprehensive multilocus phylogeny based on five molecular markers. Our results fill an important gap in mycology and will help future researchers to access the taxonomy, diversity and the evolutionary history of *Escovopsis* and related genera that inhabit the colonies of fungus-growing ants.

## Materials and methods

### Strains

A total of 102 strains of *Escovopsis*, i.e., vesiculate (n = 64) and non-vesiculate (n = 38) species, were included in this study (Additional file 1: Table S1). Of these, 30 strains were obtained from previous studies (Augustin et al. 2013; Masiulionis et al. 2015; Meirelles et al. 2015b; Montoya et al. 2019), and the remaining (n = 72) were isolated from three regions in Brazil (Novo Airão and Camp 41, state of Amazonas; Botucatu, state of São Paulo—Additional file 1: Table S1). The process of isolation, purification, and preservation of the strains followed methods outlined in Montoya et al. (2019). Briefly, from each attine colony, 21 garden fragments (0.5–1 mm^3^) were inoculated on potato dextrose agar (PDA, Neogen Culture Media, Neogen, Bury) plates (seven fragments per plate) supplemented with chloramphenicol (150 μg mL^−1^, Sigma-Aldrich, St. Louis). The plates were incubated at 25 °C in darkness and monitored daily for 7 d. When *Escovopsis* mycelia grown out, they were transferred to new PDA plates without chloramphenicol. Axenic cultures were prepared by single conidial isolation and stored in sterile distilled water kept at 8–10 °C (Castellani 1963), and in 10% aqueous solution of glycerol at − 80 °C. Both the strains isolated in this study and those obtained from other studies are deposited at the Laboratory of Fungal Ecology and Systematics (LESF—Department of General and Applied Biology, São Paulo State University (UNESP), Rio Claro, SP, Brazil) and at the UNESP—Microbial Resources Center (CRM-UNESP), Rio Claro, Brazil, under the same conditions.

### DNA extraction, PCR and sequencing

The genomic DNA of the strains was extracted using a modified CTAB method (Möller et al. 1992). Briefly, fungal aerial mycelia, grown for 7 d at 25 °C on PDA, were crushed with the aid of glass microspheres (Sigma) in lysis solution and incubated at 65 °C for 30 min. The organic phase was separated using a solution of chloroform-isoamyl alcohol (24:1). Then, the material was centrifuged (10,000*g* for 10 min), and the supernatant with the genomic DNA was collected. This extract was precipitated with 3 M sodium acetate and isopropanol and purified with two successive washes of 70% ethanol. The DNA was suspended in 30 μL of Tris–EDTA solution and stored at − 20 °C.

Five molecular markers were amplified for all newly isolated *Escovopsis* strains: the internal transcribed spacer (ITS), the large subunit ribosomal RNA (LSU), the translation elongation factor 1-alpha (*tef*1), and the RNA polymerase II protein-coding genes (*rpb*1 and *rpb*2, Additional file 1: Table S2). For strains from previous publications, we utilized previously published ITS, LSU and *tef*1 sequences, when available, and generated missing sequences for other molecular markers (Additional file 1: Table S1). Sequences of *rpb*1 and *rpb*2 for 23 strains in the genus *Escovopsioides* were also generated in this study to complete our dataset (Additional file 1: Table S1).

PCR reactions were carried out following Meirelles et al. (2015b) for ITS, Meirelles et al. (2015a) for *tef*1 and Augustin et al. (2013) for LSU (Additional file 1: Table S2). PCRs for the *rpb*1 and *rpb*2 genes (Additional file 1: Table S2) were performed in a final volume of 25 μL (4 μL of dNTPs [1.25 mM each]; 5 μL of 5 × buffer; 1 μL of bovine serum albumin (BSA) [1 mg mL^−1^]; 2 μL of MgCl_2_ [25 mM]; 1 μL of each primer [10 μM]; 0.5 μL of Taq polymerase [5 U μL^−1^], 2 μL of diluted genomic DNA [1:100] and 8.5 μL of sterile ultrapure water). All PCR reagents were from Promega, Madison. When amplification was difficult, we added 1.5 μL of dimethyl sulfoxide (DMSO), decreasing the volume of sterile ultrapure water to 7.0 μL, or we used the PuReTaq™ Ready-to-Go™ PCR kit (illustra™) following the manufacturer’s protocol. Touchdown PCR conditions were used for *rpb*1 and *rpb2*: (1) 96 °C for 5 min; (2) 15 cycles of 94 °C for 30 s, 65 °C for 1.5 min for *rpb*1 and for 1 min for *rpb*2 (the annealing temperature gradually decreased 1 °C per cycle) and 72 °C for 1.5 min for *rpb*1 and for 1 min for *rpb*2; and then (3) 35 cycles of 94 °C for 30 s, 50 °C for 1 min and 72 °C for 1 min (Additional file 1: Table S2).

Final amplicons were purified with the Wizard SV Gel and PCR Clean-up System (Promega, Madison) following the manufacturer’s protocol. Sequences (forward and reverse) were generated on an ABI3500 (ThermoFisher Scientific, Waltham), and the consensus sequences were assembled in BioEdit v. 7.1.3 (Hall 1999) or Geneious (Kearse et al. 2012). All sequences are deposited in GenBank (Additional file 1: Table S1 for accession numbers).

### Phylogenetic analyses

In order to have a complete perspective of the *Escovopsis*’ phylogenetic incongruences and their possible solutions, we performed phylogenetic analysis to: (1) know the phylogenetic placement of all strains currently treated as *Escovopsis*, and (2) provide a phylogenetic framework that establishes the foundations of the genus’ systematics.

### Phylogenetic placement of fungi treated as *Escovopsis*

We reconstructed a phylogenetic tree combining all *tef*1 sequences of fungi treated as *Escovopsis* in the literature (Currie et al. 2003; Gerardo et al. 2004, 2006b; Taerum et al. 2007, 2010; Augustin et al. 2013; Masiulionis et al. 2015; Meirelles et al. 2015a, b; Montoya et al. 2019) with our *tef*1 data set and the *tef*1 sequences from other genera of the *Hypocreales* (Additional file 1: Table S3). It was not possible to perform a multilocus analysis because few strains on the literature were sequenced for more than one molecular marker. Nonetheless, the *tef*1 gene was the one used in most of the studies published already. The final data set contained a total of 440 *tef*1 sequences (754 bp), that included vesiculate-*Escovopsis* (n = 274 strains), non-vesiculate *Escovopsis* (n = 105 strains), 60 strains from five *Hypocreaceae* genera, i.e., *Escovopsioides*, *Hypomyces* (along with species under its anamorphic genus *Cladobotryum*) (Põldmaa 2011), *Protocrea, Sphaerostilbella,* and *Trichoderma*, and *Lecanicillium antillanum* CBS 350.85 as the outgroup (Additional file 1: Table S3). The *tef*1 sequences of species described in Marfetán et al. (2018) were not included in this analysis because they do not align with the *Escovopsis* sequences from the previous studies.

The data set was first aligned in MAFFT v.7 (Katoh and Standley 2013), and phylogenetic trees were reconstructed using maximum likelihood (ML) and Bayesian inferences (BI) in RAxML (Stamatakis 2014) and MrBayes v.3.2.2 (Ronquist et al. 2012), respectively. The nucleotide substitution model was GTR for ML and K80 + G for BI and was calculated in jModelTest 2 (Darriba et al. 2012), using the Akaike Information Criterion (AIC) with 95% confidence intervals. For ML analysis, 1000 independent trees and 1000 bootstrap replicates were performed, while for BI two separate runs (each consisting of three hot chains and one cold chain) were carried out. In the last case, five million generations of the Markov Chain Monte Carlo (MCMC) were enough to reach convergence (standard deviation (SD) of split frequencies fell below 0.01). To generate final BI tree, the first 25% of trees were discarded as burn-in. The final tree was edited in FigTree v.1.4 (http://tree.bio.ed.ac.uk/software/figtree/) and Adobe Illustrator CC v.17.1.

### Phylogenetic framework for *Escovopsis’* systematics

Phylogenetic analyses were performed at order and family levels. The objective of the analyses at the order level was to investigate whether all clades formally described as *Escovopsis* belong to the *Hypocreaceae* close to the *Cordycipitaceae* as previously observed by Augustin et al. (2013). The objective of the analyses at the family level was to investigate whether the vesiculate species of *Escovopsis* form a monophyletic clade, separating them from the non-vesiculate species*,* as previously observed by Montoya et al. (2019). Besides, we wanted to know if the monophyly of all those clades remains constant considering the Genealogical Concordance Phylogenetic Species Recognition (GCPSR) concept (Taylor et al. 2000), although it was applied to the consideration on the generic differentiation.

*Escovopsis* species described by Marfetán et al. (2018) were not included in this analyses because: (1) the inavailability of ITS, *rpb*1, and *rpb*2 sequences, (2) the *tef*1 sequences provided by the authors do not align with the *Escovopsis* sequences from other studies (Augustin et al. 2013; Masiulionis et al. 2015; Meirelles et al. 2015a, b; Montoya et al. 2019), and (3) the LSU sequences from *E. longivesica*, provided by the authors, do not have similarity with *Escovopsis* but with *Ceriporia alachuana* (95.4% identity, *E. longivesica* E5, E9) and *Penicillium glabrum* (95.3% identity, *E. longivesica* E10). However, the remaining LSU sequences, identical to *Escovopsis*, generated by the authors were combined with the LSU data to show its phylogenetic placement (Additional file 1: Table S4 and Additional file 2: Fig. S1).

For the ‘order-level’, we used a concatenated data set that included 145 sequences for LSU (625 bp), 143 for *rpb*1 (851 bp), 143 for *rpb*2 (980 bp), and 144 for *tef*1 (849 bp) from six families of the order *Hypocreales* (*Bionectriaceae*, *Clavicipitaceae*, *Cordycipitaceae*, *Hypocreaceae*, *Nectriaceae*, and *Ophiocordycipitaceae*, Additional file 1: Table S5). For this analysis, we generated the *rpb*1 and *rpb*2 sequences for *Escovopsis* (including the nine ex-type strains) and *Escovopsioides nivea* (including the ex-type strain). All other sequences were obtained from the NCBI GenBank database (Currie et al. 2003; Sung et al. 2008; Augustin et al. 2013; Masiulionis et al. 2015; Meirelles et al. 2015a, b; Montoya et al. 2019, Additional file 1: Table S5). The *Stachybotrys* clade was used to root the tree (Sung et al. 2008).

Multiple loci were used to address the ‘family-level’ questions, and all possible combinations of ITS, LSU, *tef*1, and *rpb*2 (25 combinations) were analyzed (GCPSR concept; Taylor et al. 2000). Data sets included 133 sequences of ITS (707 bp), LSU (594 bp), *tef*1 (758 bp), and *rpb*2 (1023 bp); and 131 sequences of *rpb*1 (725 bp). The sequences represented 102 strains from the *Escovopsis* clade (vesiculate (n = 64) and non-vesiculate (n = 38) species, including the nine ex-type strains), 30 strains from five *Hypocreaceae* genera (*Escovopsioides*, *Hypomyces* along with species under its anamorphic genus *Cladobotryum*, *Protocrea, Sphaerostilbella,* and *Trichoderma*), and *Lecanicillium antillanum* CBS 350.85 as the outgroup (Additional file 1: Table S1).

For all analyses, datasets were first aligned separately for each gene in MAFFT v.7 (Katoh and Standley 2013). The nucleotide substitution model for each alignment was calculated in jModelTest 2 (Darriba et al. 2012), using the Akaike Information Criterion (AIC) with 95% confidence intervals. Then, the datasets were concatenated in Winclada v.1.00.08 (Nixon 2002). All phylogenetic trees were constructed using ML in RAxML v.8 (Stamatakis 2014) and BI in MrBayes v.3.2.2 (Ronquist et al. 2012). For ML, we estimated 1000 independent trees and performed 1000 bootstrap replicates using the GTR + I + G model for each partition independently. For BI analyses, we carried out two separate runs (each consisting of three hot chains and one cold chain) using the GTR + I + G model for each partition independently; for all analyses, two million generations of the Markov Chain Monte Carlo (MCMC) were enough to reach convergence [standard deviation (SD) of split frequencies fell below 0.01]. To generate BI trees, the first 25% of trees were discarded as burn-in. Trees were edited in FigTree v.1.4 (http://tree.bio.ed.ac.uk/software/figtree/) and Adobe Illustrator CC v.17.1.

### Morphology

We examined the microscopic structures of nine ex-type cultures of *Escovopsis* species (*E. aspergilloides*, *E. clavata*, *E. kreiselii*, *E. lentecrescens*, *E. microspora*, *E. moelleri*, *E. multiformis*, *E. trichodermoides*, and *E. weberi*), representing the known diversity of the genus, to determine if those morphological features support the results observed in phylogenetic analyses.

To assess and compare the microscopic structures (i.e.*,* conidiophores, conidiophore branches, vesicles, conidiogenous cells, conidia, and chlamydospores) and their features (i.e., shape and pattern), we carried out slide culture preparations on PDA. To do so, we placed three cylinders of PDA (ca. 5 mm in diameter × 5 mm in height) on a sterilised microscopic slide, and we then inoculated each fragment with conidia of the fungus. Each inoculated fragment was covered with a coverslip and incubated at 25 °C for 4–7 d in the dark. After that, the fragments of PDA were removed and the coverslips with fungal mycelia were placed on slides with a drop of lactophenol. The slides were examined under a light microscope (DM750, Leica, Wetzlar), and the microscopic fungal structures were photographed using the software LAS EZ v.4.0 (Leica, Wetzlar).

## Results

### Phylogenetic placement of fungi previously known as *Escovopsis*

**Fig. 1:**
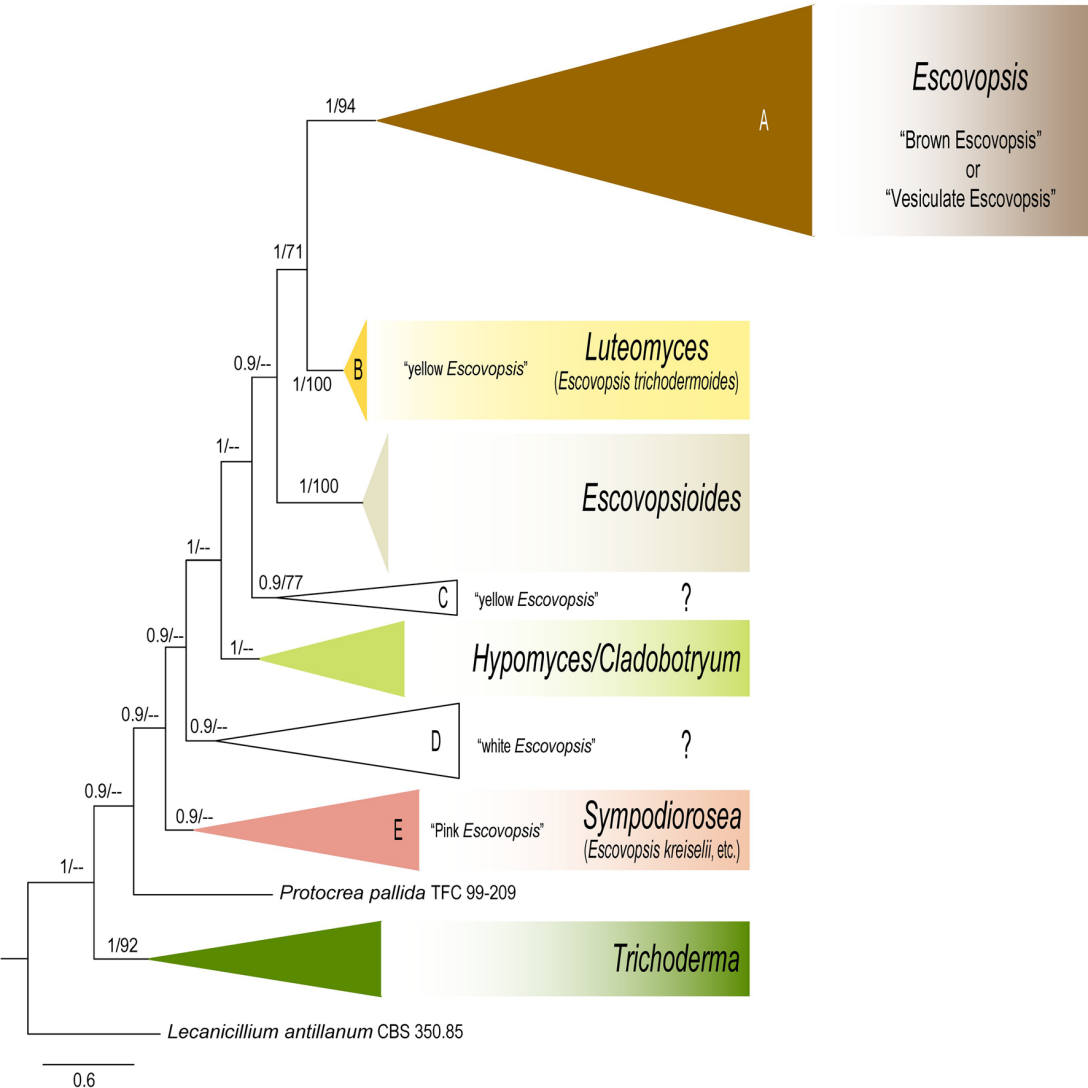
Collapsed phylogenetic tree (from Additional file 3: Fig. S2) indicating the placement of every isolate previously treated as *Escovopsis*. The tree shown was inferred using Bayesian Inference (BI). The tree gathers all available *tef*1 sequences found in the literature with the data set used in this study, including the sequences of the nine *Escovopsis* ex-type cultures. The tree contains a total of 440 sequences which include: 274 strains of vesiculate-*Escovopsis* (Clade A), 105 strains of non-vesiculate *Escovopsis* (Clades B, C, D, E) and 60 strains from four genera, i.e., *Escovopsioides*, *Hypomyces* (along with species under its anamorphic genus *Cladobotryum*), *Protocrea*, and *Trichoderma* in the *Hypocreaceae*. *Lecanicillium antillanum* CBS 350.85 is used as the outgroup of the tree (see Additional file 1: Table S3 for all strains and their associated metadata used to infer this phylogenetic tree). There is only information, in the literature, on the colour of the colonies of the clades C and D, but the microscopic features of these clades are unknown. Numbers on branches indicate BI posterior probabilities (PP) and Maximum Likelihood bootstrap support values (MLB), respectively. Hyphens (--) indicate MLB < 70%

The *tef*1 phylogenetic tree showed nine well supported monophyletic clades within the *Hypocreaceae* (Fig. 1 and Additional file 3: Fig. S2). The four previously recognized genera of the *Hypocreaceae*, i.e., *Escovopsioides*, *Hypomyces* (along with species under its anamorphic genus *Cladobotryum*), *Protocrea*, and *Trichoderma,* preserved their monophyly. On the other hand, the genus *Escovopsis* (clades A–E) was found polyphyletic and scattered throughout the family (Fig. 1 and Additional file 3: Fig. S2).

While clades A–E are treated as *Escovopsis*, clades A (vesiculate *Escovopsis*; the type species of *Escovopsis*, i.e., *E*. *weberi* belongs to this clade), B (*E*. *trichodermoides*), and E (*E*. *kreiselii*) are the only clades containing formally described *Escovopsis* species, so far. Although clades A and B are sister clades, they are far away separated from the clade E (paraphyletic clade; Fig. 1 and Additional file 3: Fig. S2). On the other hand, clades C and D introduced by Gerardo et al. (2006b) as yellow and white *Escovopsis*, respectively (Fig. 1 and Additional file 3: Fig. S2), are more closely related to *Escovopsioides* and *Hypomyces* than to the clade A of *Escovopsis* (paraphyletic).

### Phylogenetic framework to redefine the genus *Escovopsis*

**Fig. 2:**
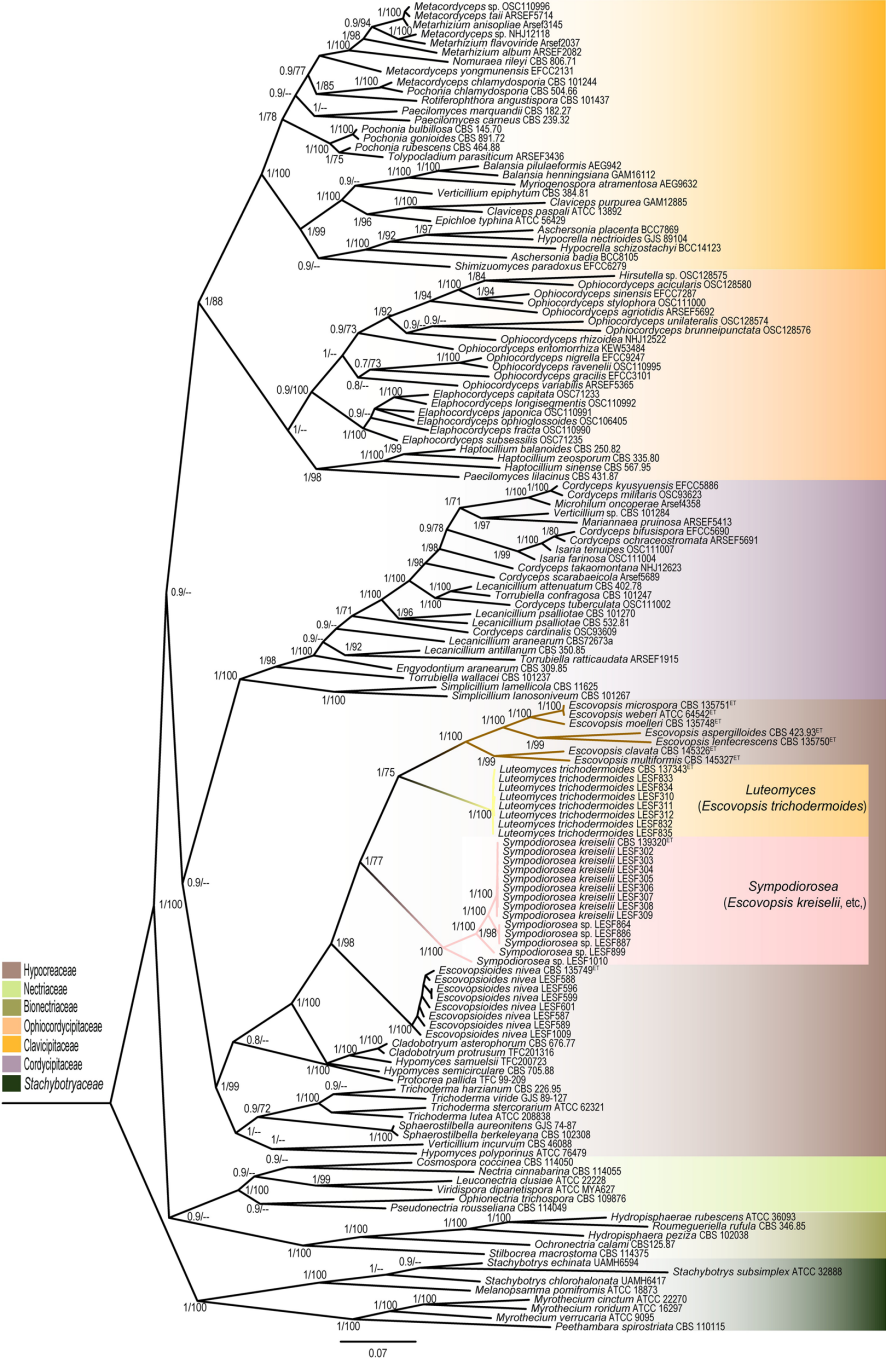
Phylogenetic tree indicating the placement of *Escovopsis* (brown branches), *Sympodiorosea* (pink branches) and *Luteomyces* (yellow branches) within the *Hypocreales*. The tree shown was inferred using Bayesian Inference (BI). Highlighted clades in different colours represent six different families (*Bionectriaceae, Clavicipitaceae, Cordycipitaceae, Hypocreaceae, Nectriaceae*, and *Ophiocordycipitaceae*) in the *Hypocreales*. The analysis was based on concatenated sequences of LSU, *tef*1, *rpb*1 and *rpb*2 (See Additional file 1: Table S5 for all strains and their associated metadata used to infer this phylogenetic tree). Numbers on branches indicate BI posterior probabilities (PP) and Maximum Likelihood bootstrap support values (MLB), respectively. Hyphens (--) indicate MLB < 70%. We used eight species from the family *Stachybotryaceae* (dark green box) to root the tree. ET indicates ex-type cultures

Phylogenetic analyses (BI and ML) at the order level placed both the vesiculate and non-vesiculate *Escovopsis* within the *Hypocreaceae* (Fig. 2). Vesiculate *Escovopsis*, i.e., *E. aspergilloides*, *E. clavata*, *E. lentecrescens*, *E. microspora*, *E. moelleri*, *E. multiformis* and *E. weberi*, formed a monophyletic clade in both the BI and ML analyses (BI Posterior Probability (PP) = 1; ML bootstrap value (MLB) = 100) (Fig. 2). They can be called as the true *Escovopsis*. On the other hand, non-vesiculate *Escovopsis*, i.e., *E. kreiselii* and *E. trichodermoides*, formed well-supported, monophyletic clades (PP = 1, MLB = 100, each one) respectively outside of the vesiculate *Escovopsis* and are separated from all other *Hypocreaceae* genera (Fig. 2).

**Fig. 3:**
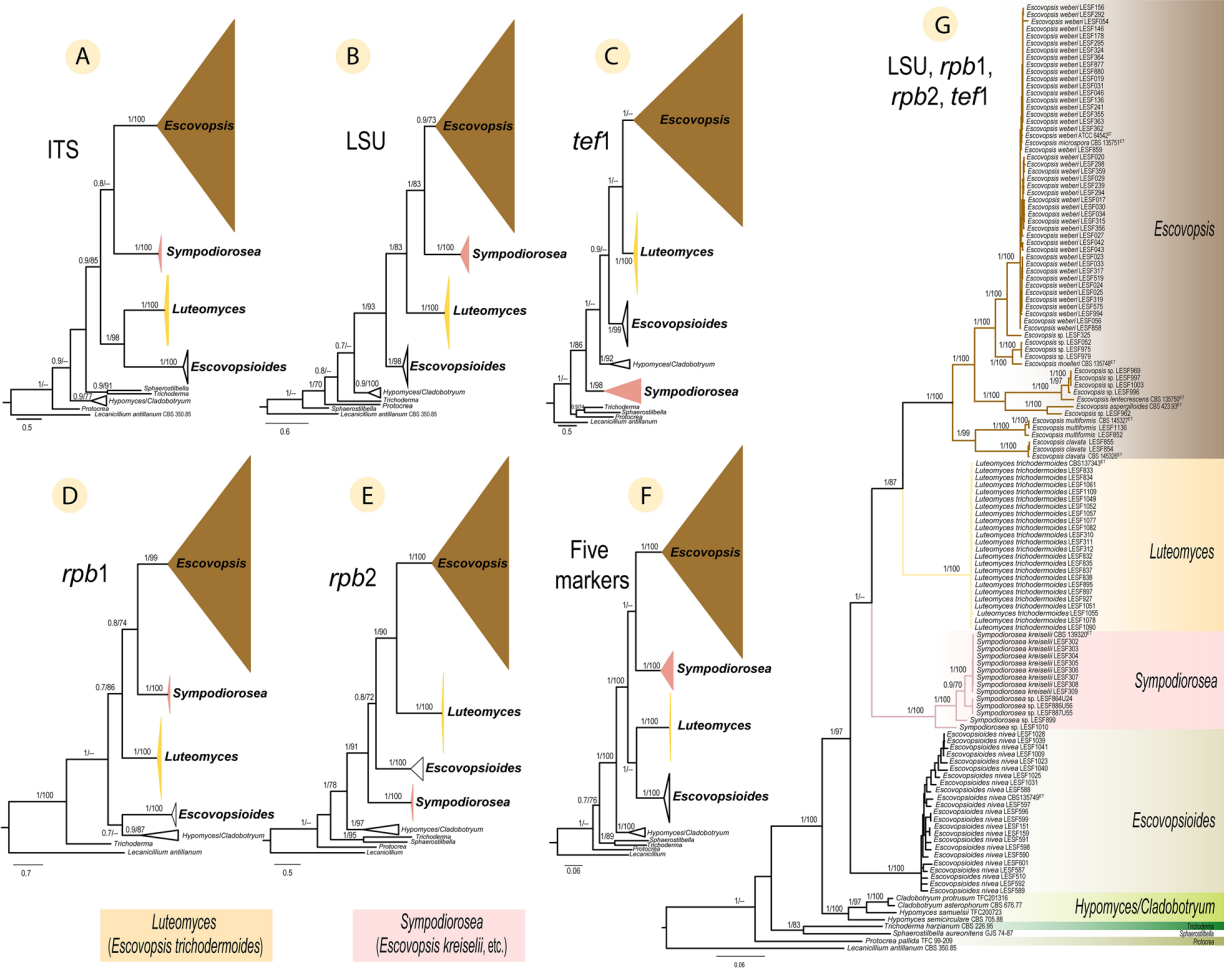
Phylogenetic placement of *Escovopsis*, *Sympodiorosea* and *Luteomyces* within the *Hypocreaceae*. Phylogenies shown were inferred using Bayesian Inference (BI) and are separated for each region (molecular marker): (**A**) ITS, (**B**) LSU, (**C**) *tef*1, (**D**) *rpb*1, and (**E**) *rpb*2. The remaining two trees are based on concatenated datasets of (**F**) all five markers, and (**G**) four of the five markers (LSU, *rpb*1, *rpb*2, and *tef*1). Numbers on branches indicate BI posterior probabilities (PP) and Maximum Likelihood bootstrap support values (MLB), respectively. Hyphens (--) indicate MLB < 70%. *Lecanicillium antillanum* CBS 350.85 was used as the outgroup. ET indicates ex-type cultures. See Table S1 for all strains and their associated metadata used to infer these phylogenetic trees

Analyses on the family level based on GCPSR (Taylor et al. 2000) revealed that *Hypomyces*, *Escovopsioides*, *E. kreiselii*, *E. trichodermoides*, and vesiculate-*Escovopsis* each form separate, monophyletic clades (Fig. 3). The phylogenetic placement of the five clades varies depending on the molecular marker used for the analysis. The analyses made separately with ITS, LSU, and *rpb*1 (Fig. 3A, B, D, respectively), as well as the concatenated analysis based on the five markers (Fig. 3F) indicate vesiculate-*Escovopsis*, and *E. kreiselii* as sister clades. The analyses made separately with *tef*1 and *rpb*2 (Fig. 3C, E, respectively), and the concatenated analysis based on four markers (LSU, *tef*1, *rpb*1, and *rpb*2—Fig. 3G) indicate vesiculate-*Escovopsis* and *E. trichodermoides* as forming sister clades. In addition, the analyses performed with ITS and the five concatenated-markers showed *Escovopsioides* and *E. trichodermoides* forming a monophyletic clade (PP = 1, MLB = 98—Fig. 3A and PP = 1, MLB < 70—Fig. 3F, respectively). The analysis based on *rpb*1, however, indicated *Escovopsioides* forming a monophyletic clade with *Hypomyces* (Fig. 3D), while analysis based on *tef*1 placed *Hypomyces* between *Escovopsioides* and *E. kreiselii* (Fig. 3C). Finally, the analyses carried out with LSU, *tef*1, and *rpb*2, separately (Fig. 3B, C, E, respectively), and the concatenated analysis (LSU, *tef*1, *rpb*1 and *rpb*2—Fig. 3G) placed *Hypomyces*, *Escovopsioides*, *E. kreiselii*, *E. trichodermoides*, and vesiculate-*Escovopsis* in well-supported, monophyletic clades (clearly separated from one another). It is important to highlight that the analysis at this level also showed that *E. aspergilloides*, *E. clavata*, *E. lentecrescens*, *E. moelleri*, and *E. multiformis* and four unnamed phylogenetic species, i.e., LESF 325, LESF 962, and strain groups (LESF 052, LESF 975 and LESF 979) and (LESF 969, LESF 997, LESF 1003 and LESF 996) formed well-supported monophyletic clades, that were clearly separated from one another (Fig. 3G). Nonetheless, the ex-type cultures of *E. weberi* and *E. microspora* grouped together with 45 other isolates in the same well-supported clade (Fig. 3G).

Regarding the five species described by Marfetán et al. (2018), our analysis of the available LSU data indicates that they form two different clades closely related to *E. aspergilloides* and *E. lentecrescens* (Additional file 2: Fig. S1). Specifically, the four strains of *E. primorosea* formed a well-supported monophyletic clade (Additional file 2: Fig. S1), however *E. atlas*, *E. catenulata*, *E. pseudoweberi*, and five strains named as *E. weberi* by the authors formed a single monophyletic clade (Additional file 2: Fig. S1). This result apparently does not support the new species hypothesis. Future research will be required to clarify the existence of these species.

### Morphological evidence to redefine the genus *Escovopsis*

Based on morphological analysis, three different groups of fungi are clearly distinguished in the genus *Escovopsis*. They are: (1) the vesiculate group, composed of *E. aspergilloides*, *E. clavata*, *E. lentecrescens*, *E. microspora*, *E. moelleri*, *E. multiformis*, *E. weberi*, together with the four unnamed phylogenetic species, i.e., LESF 325, LESF 962 and strain groups A (LESF 052, LESF 975 and LESF 979) and B (LESF 969, LESF 996, LESF 997, and LESF 1003); (2) the non-vesiculate group 1, including *E. kreiselii* and the three unnamed phylogenetic species, i.e., LESF 889, LESF 1010 and the strain group (LESF 864, LESF 886 and LESF 887); and (3) the non-vesiculate group 2 as *E. trichodermoides*.

**Fig. 4:**
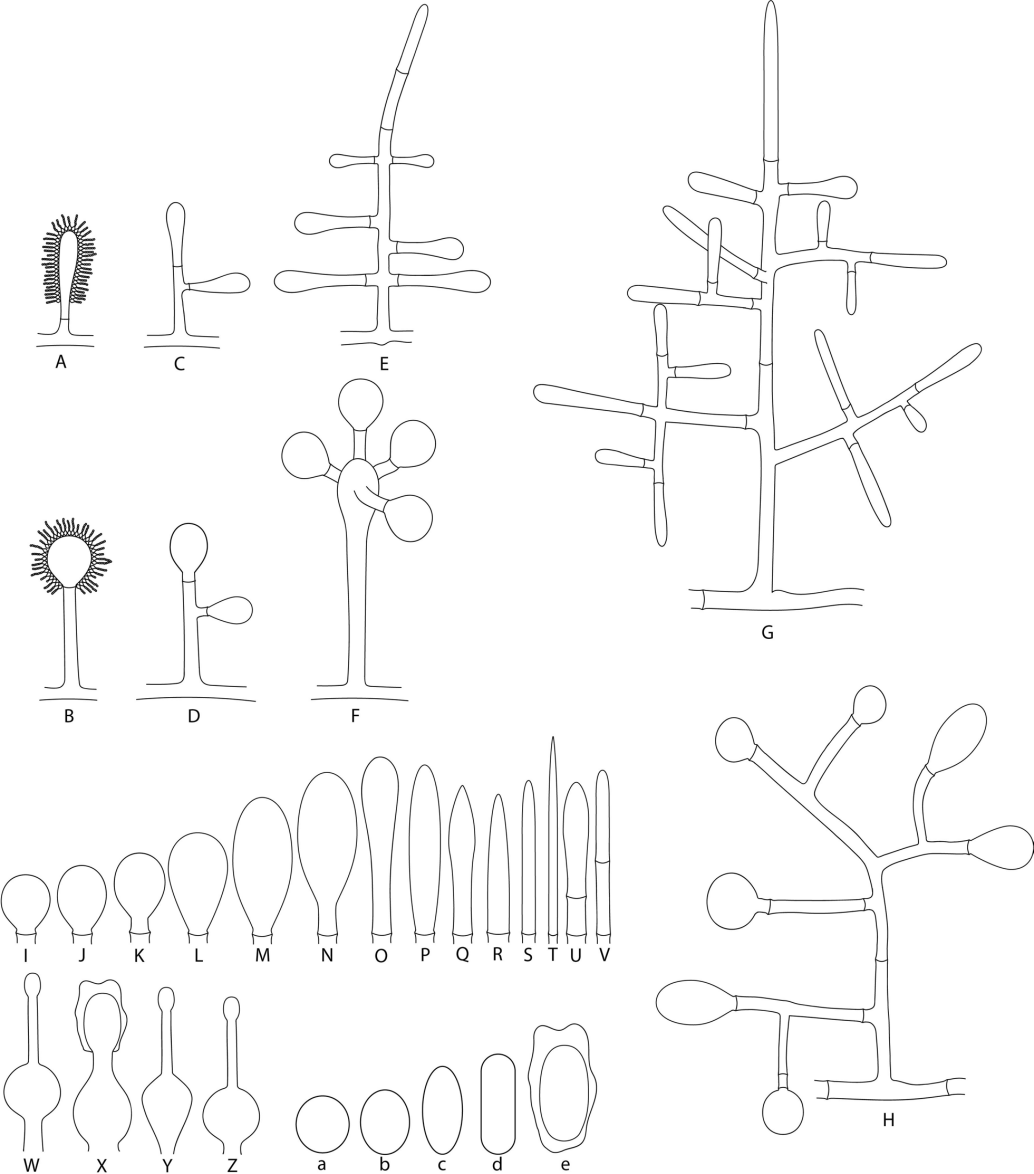
Illustration of microscopic structures of the genus *Escovopsis*. **A**, **B** Mono-vesiculate conidiophores. **C**–**H** Poly-vesiculate conidiophores. F: Conidiophore with “swollen cell”. **I**–**V** Different shapes of vesicles (**I**: Globose, **J**: Subglobose, **K**: Capitate, **L**: Obovoid, **M**: Prolate, **N**: Spatulate, **O**: Clavate, **P**: Cymbiform, **Q**: Lanceolate, **R**: Subulate, **S**: Cylindric, **T**: Filiform, **U**: Clavate-septate, **V**: Cylindric-septate; only vesicles without phialides are illustrated). **W**–**Z**: Different shapes of the phialides. a–e: Different shapes of conidia (a: Globose, b: Subglobose, c: Ellipsoidal, d: Oblong, e: Oblong-ornamented)

As the first group, vesiculate *Escovopsis* spp. present conidiophores with one apical vesicle (mono-vesiculate) or with two to more vesicles (poly-vesiculate) (Fig. 4). Mono-vesiculate conidiophores (Fig. 4A, B) emerge from aerial mycelia in an alternating and opposite pattern. Poly-vesiculate conidiophores (Fig. 4C–H) also emerge from the aerial mycelia and can present short branches composed of one or two cells or long branches with multiple cells. Some poly-vesiculate conidiophores have a swollen cell at the apex from where branches emerge (Fig. 4F). Conidiophores that have swollen cells form their branches only from swollen cells. Vesicles, mostly composed of a single cell (non-septate vesicles—Fig. 4I–T), and rarely of two cells (septate vesicles—Fig. 4U, V). Vesicles can emerge from the apices and axes of the conidiophore and its branches (usually only one vesicle in the short branches and two or more in long branches) (Fig. 4G, H). Vesicles are connected to the hypha from where they emerge by a basal septum or by a peduncle made up of one or two cells. Vesicles can exhibit different shapes (globose, subglobose, capitate, obovoid, prolate, spatulate, clavate, cymbiform, lanceolate, subulate, cylindric, filiform, clavate-septate, cylindric-septate) (Fig. 4I–V) and sizes, depending on the species. Phialides (i.e., enteroblastic conidiogenous cells; Fig. 4W–Z) emerge mainly from the vesicles and less frequently from the aerial mycelia. These structures are lageniform and ampulliform, forming conidia. Main differences of phialides among the different species are mostly related to the sizes of their base, the widened part and the neck (Fig. 4W–Z). Conidia are single-celled, produced in chains from the phialides (enteroblastic conidiogenesis), and can vary in shape (globose, subglobose, ellipsoidal, oblong, and oblong-ornamented) (Fig. 4a–e). Conidia can be smooth or with ornamentations on cell walls (Fig. 4e). Chlamydospores are rarely observed.

**Fig. 5:**
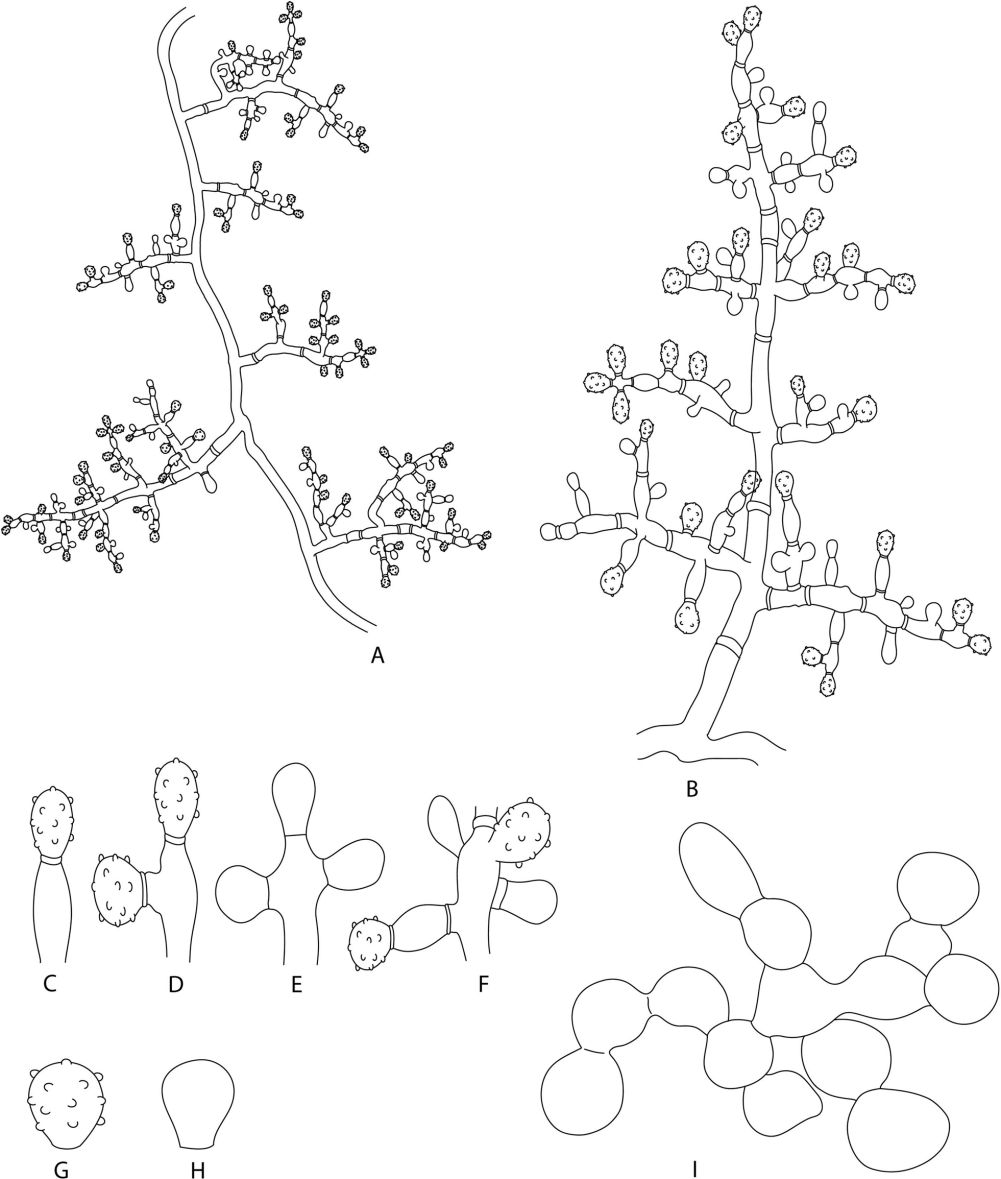
Illustration of the microscopic structures of *Luteomyces trichodermoides* (syn. *Escovopsis trichodermoides*). **A** Pattern of disposition of conidiophores on aerial mycelium. **B** A conidiophore. **C**–**F** Poorly differentiated conidiogenous cells (holoblastic determinate conidiogenous cells with synchronous arrangement). **G** Conidium with ornamentation. **H** Smooth conidium. **I** Chlamydospores

The two non-vesiculate groups form conidiophores remarkably different from those of the vesiculate group. Conidiophores of *E. trichodermoides* lack vesicles and are mostly pyramidal (*Trichoderma*-like), with one to six short levels of branches arising at more or less right angles from the conidiophore axis (Fig. 5A, B). In contrast to vesiculate *Escovopsis*, this species has poorly differentiated conidiogenous cell (i.e., holoblastic determinate conidiogenous cells with synchronous arrangement; Fig. 5C–F) with ampulliform shapes producing either solitary (Fig. 5C) or up to three conidia (Fig. 5D–F). The conidia are subglobose to obovate, yellow–brown, mostly ornamented (verrucose; Fig. 5G) and rarely smooth (Fig. 5H). Unlike the vesiculate *Escovopsis* spp., *E*. *trichodermoides* regularly forms chlamydospores (Fig. 5I).

**Fig. 6:**
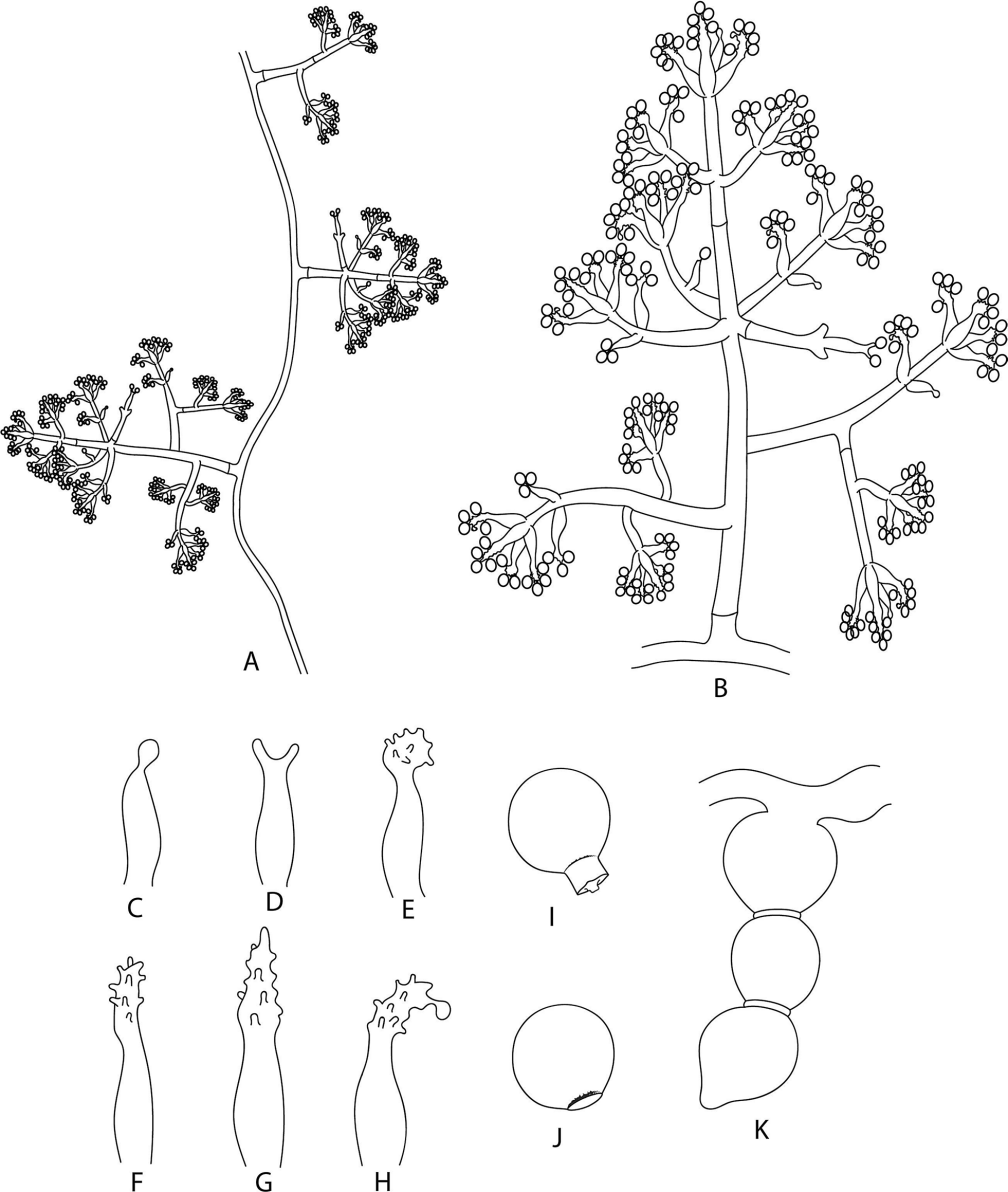
Illustration of the microscopic structures of *Sympodiorosea kreiselii* (syn*. Escovopsis kreiselii*). **A** Pattern of disposition of conidiophores on aerial mycelium. **B** A conidiophore. **C**–**H** Shapes of the sympodial conidiogenous cells. **I** Conidia with denticle (detached from the conidiogenous cell), **J** Conidia with a hole (when the denticle remains on the conidiogenous cell). **K** Chlamydospores in chain

Conidiophores of *E. kreiselii* also lack vesicles and are formed on the aerial mycelium in an alternated or opposite pattern (Fig. 6A, B), and are more branched (with irregular branching conformation) than those of vesiculate *Escovopsis*. Conidiophores are usually irregularly shaped but some can be pyramidal (*Trichoderma*-like). Conidiogenous cells of *E. kreiselii* are not phialidic but sympodial (i.e., holoblastic proliferous conidiogenous cells; Fig. 6C–H), and they are formed on the apex and on both the main and branch axes of the conidiophore (Fig. 6B). Conidia of *E. kreiselii* are solitary, globose to subglobose, smooth but with thick-walled, light brown to dark brown, and usually with a denticle (Fig. 6I) or with a lesion (when the denticle remains on the conidiogenous cell; Fig. 6J). Like *E*. *trichodermoides*, *E. kreiselii* also forms chlamydospores regularly (Fig. 6K).

## Taxonomy

Phylogenetic analyses in this study unambiguously demonstrate that species previously treated as *Escovopsis* form three distinct monophyletic clades (Fig. 3). These groups also differ significantly from one another in morphological characters. The first clade (Fig. 3G) includes the ex-type cultures of *E. weberi* (the type species of *Escovopsis*), *E. aspergilloides*, *E. clavata*, *E. lentecrescens*, *E. microspora*, *E. moelleri*, *E. multiformis*, and four undescribed phylogenetic species, i.e., two strains (LESF 325 and LESF 962) and two groups of strains (LESF 052, LESF 975 and LESF 979; LESF 969, LESF 996, LESF 997, and LESF 1003), which are all vesiculate species. We recognize these species as true *Escovopsis* because they form a monophyletic clade including the type species of the genus and preserve the main character (presence of vesicles on conidiophores) that gave origin to the generic name (Muchovej and Della Lucia 1990). Therefore, we redefine and restrict *Escovopsis* to include only vesiculate species (Fig. 3G) and the description of the genus is emended based on the criteria adopted in this study.

The two remaining clades (Fig. 3G) are made up of (1) *E. kreiselii* together with the three unnamed phylogenetic species, i.e., LESF 889, LESF 1010 and a group of strains (LESF 864, LESF 886 and LESF 887), and (2) *E. trichodermoides* (monotypic clade), which are only non-vesiculate *Escovopsis* species, respectively. Here, we establish two new genera, *Sympodiorosea* and *Luteomyces*, for reassigning these two non-vesiculate clades.

***Escovopsis*** J.J. Muchovej & Della Lucia, *Mycotaxon* **37**: 192 (1990).

(Fig. 4).

MycoBank: MB 11249.

*Type species*: *Escovopsis weberi* J.J. Muchovej & Della Lucia.

*Original description*: Muchovej and Della Lucia (1990), emended by Meirelles et al. (2015a).

*Description*: Monophyletic genus belonging to the *Hypocreaceae* that presents mono- to poly-vesiculate conidiophores formed on hyaline aerial mycelia (Fig. 4A–H). *Vesicles* terminal, mostly non-septate (Fig. 4I–T), rarely with one septum (Fig. 4U, V), various shaphed (globose, subglobose, capitate, obovoid, prolate, spatulate, clavate, cymbiform, lanceolate, subulate, cylindric, filiform, clavate-septate, cylindric-septate (Fig. 4I–V). *Conidiogenous cells* phialides, hyaline, with a thin base, a swollen section and a thin neck (Fig. 4W–Z), formed on vesicles. *Conidia* smooth or ornamented, aseptate, hyaline to brown, various shaped (globose, subglobose, ellipsoidal, cylindric; Fig. 4a–e), produced in chains.

*Notes: Escovopsis* is phylogenetically placed within the *Hypocreaceae* as a sister clade of *Luteomyces* (Fig. 3G). *Escovopsis* exhibits faster growth and different colony colour (brown) than *Luteomyces* (yellow). Unlike *Luteomyces*, which presents poorly diferenciated conidiogenous cells (i.e., holoblastic determinate conidiogenous cells with synchronous arrangement), *Escovopsis* forms phialides (i.e., enteroblastic conidiogenous cells). The main feature of this genus is the presence of conidiophores with terminal vesicles that differentiate it from its sister clade and from all other known genera in the *Hypocreaceae*.

***Luteomyces*** Q.V. Montoya & A. Rodrigues, **gen. nov.**

**(**Fig. 5).

MycoBank: MB 835150.

*Etymology:* “*Luteomyces*” based on the colour exhibited by the colonies of the type species.

*Diagnosis:* Similar to *Escovopsis* and *Sympodiorosea* in the way it begins to grow, i.e., dense germination and forming stolon-like mycelia. However, *Luteomyces* differs from these genera and other known genera in the *Hypocreaceae* by its poorly differentiated holoblastic conidiogenous cells.

*Type species*: *Luteomyces trichodermoides* (M. Cabello et al*.*) Q.V. Montoya & A. Rodrigues 2021.

*Description*: Monophyletic genus in the *Hypocreaceae*. *Colonies* form floccose, white, beige and yellow mycelia, stolons, beige to yellow soluble pigments. *Conidiophores* formed on aerial mycelia, alternated, usually arising at right angles (Fig. 5A), smooth-walled, pyramidal shape (Fig. 5B). *Conidiogenous cells* poorly differenciated, holoblastic, determinate, in synchronous arrangement (Fig. 5C–F) on the apices and axes of conidiophores and their branches, solitary, ampulliform to lageniform (Fig. 5B). *Conidia* solitary, dry, smooth or ornamented (Fig. 5G, H), yellow to light-brown. *Chlamydospores* abundant, hyaline, smooth (Fig. 5I).

*Notes: Luteomyces* is phylogenetically placed in the *Hypocreaceae* as a sister clade of *Escovopsis* (Fig. 3G). Nonetheless, *Luteomyces* grows slower and has different colony colour (mainly yellow) than *Escovopsis* (mainly brown), and forms conidiophores without vesicles and large number of chlamydospores (rarely observed in *Escovopsis*).

***Luteomyces trichodermoides*** (M. Cabello et al*.*) Q.V. Montoya & A. Rodrigues, **comb. nov**.

(Fig. 5).

MycoBank: MB 835152.

*Basionym*: *Escovopsis trichodermoides* M. Cabello et al*.*, *Antonie van Leeuwenhoek* **107**: 737 (2015).

*Type*: **BRAZIL**: Rio Claro, São Paulo, 22° 23′ 46.93″ S; 47° 32′ 40.12″ W, isolated from the upper part of a fungus garden of *Mycocepurus goeldii*, 13 Aug 2011. *V. E. Masiulionis* (14.0662—CBS holotype, preserved as a freeze-dried sample; VEM001, CBS 137343, CBMAI 1620, LPSC 1176—ex-type cultures).

*Sequences*: KJ485699 (ITS), KF033128 (*tef*1), MF116052 (LSU), MT305417 (*rpb*1), and MT305542 (*rpb*2).

***Sympodiorosea*** Q.V. Montoya & A. Rodrigues, **gen. nov**.

(Fig. 6).

MycoBank: MB 835147.

*Etymology*: “*Sympodio*” refers to the sympodial conidiogenous cells, and “*rosea*” to the colony colour of the type species.

*Diagnosis*: Similar to *Escovopsis* and *Luteomyces* in the way it begins to grow, i.e., dense germination and forming stolon-like mycelia. However, *Sympodiorosea* differs from these genera and other known genera in the *Hypocreaceae* by its holoblastic sympodial proliferous conidiogenous cells.

*Type species*: *Sympodiorosea kreiselii* (L.A. Meirelles et al*.*) Q.V. Montoya & A. Rodrigues 2021.

*Description*: Monophyletic genus in the *Hypocreaceae*. *Colonies* form inconspicuous to floccose, white, pale-beige, pink, brown aerial mycelia. *Conidiophores* formed on aerial mycelia (Fig. 6A), alternate or opposite, usually at right angles, with irregular branching conformation (Fig. 6A, B). *Conidiogenous cells* holoblastic, sympodial, proliferous (Fig. 6C–H), in pairs or in verticils on the apices of conidiophores and their branches, and solitary, alternate or opposite, on both the axes of the conidiophore and their branches (Fig. 6B). *Conidia* formed solitary, globose to subglobose, smooth or rough (thick-walled), light-brown to dark-brown, with denticles or lesions like holes (Fig. 6I, J). *Chlamydospores* commonly formed (Fig. 6K).

*Notes: Sympodiorosea* is phylogenetically placed in the *Hypocreaceae* near the genera *Luteomyces* and *Escovopsioides* (Fig. 3G). However, *Sympodiorosea* grows slower and has different colony colour (pink) than *Luteomyces* (yellow) and *Escovopsioides* (white). *Sympodiorosea* forms more branched conidiophores than *Luteomyces* and *Escovopsioides*.

***Sympodiorosea kreiselii*** (L.A. Meirelles et al*.*) Q.V. Montoya & A. Rodrigues, **comb. nov**.

(Fig. 6).

MycoBank: MB 835148.

*Basionym*: *Escovopsis kreiselii* L.A. Meirelles et al*.*, *PLoS One* **10** (e0112067): 7 (2015).

*Type:***BRAZIL**: Santa Catarina, Florianópolis, Praia da Joaquina, 27° 37′ 50.01″ S; 48° 27′ 3.64″ W, elev. 1 m, isolated from fungus garden of *Mycetophylax morschi*, 6 Mar 2009. *A. Rodrigues* (CBS H-22062, dried culture on PDA – holotype; LESF 53, CBS 139320, CBMAI 1691—ex-type cultures).

*Sequences*: KJ808767 (ITS), KJ808766 (*tef*1), KJ808765 (LSU), MT305418 (*rpb*1), and MT305543 (*rpb*2).

## Discussion

Here, we provide the basis for the systematics of *Escovopsis* and related genera using a set of morphological characters, and comprehensive multilocus phylogenetic analyses. Our results supported the separation of species previously treated as *Escovopsis* into three distinct genera. Accordingly, we redefine and restrict *Escovopsis* to vesiculate species, and we describe *Sympodiorosea* to accommodate *E. kreiselii* and *Luteomyces* to accomodate *E. trichodermoides*. This study provides a long-awaited revision of *Escovopsis* systematics and related genera, thus helping future researchers to assess the diversity and evolutionary history of these fungus-growing ant associates.

Genera of the *Hypocreaceae* have morphological features that differentiate them from one another (Jaklitsch et al. 2008; Jaklitsch 2009; Põldmaa 2011). Due to the morphological plasticity of fungi (Slepecky and Starmer 2009; Wrzosek et al. 2017), variations in the shades of colours expressed by these organisms are highly expected. Nonetheless, the prevalent brown colour of *Escovopsis* colonies is a unique feature of this genus within the family. Curiously, other genera in the same family also exhibit unique colours, as is the case for the genus *Trichoderma,* which is characterized by its mostly green colonies (Jaklitsch 2009), and *Escovopsioides,* which is characterized by its white colour (Augustin et al. 2013). While *Sympodiorosea* and *Luteomyces* are currently monotypic genera, phylogenetic analysis suggests there are more *Sympodiorosea* species waiting to be described (Figs. 2, 3G). Colours exhibited by these genera (pink and yellow, respectively) are also unique within the *Hypocreaceae*. Interestingly, the separation of these clades by the colour patterns was previously observed by other authors (Gerardo et al. 2006b; Meirelles et al. 2015b), but the lack of a deep morphological analysis prevented reaching the conclusion that they were different genera.

Microscopic features also differentiate *Escovopsis* from other genera in the *Hypocreaceae*. Conidiophores with terminal-vesicles producing phialides, present in *Escovopsis*, are a unique feature of the genus within this family. *Escovopsioides* also presents vesicles, however they are formed intercalary on aerial mycelia and solitary on the apex of the conidiophores. Furthermore, the vesicles of this genus are smaller and have fewer phialides than those of *Escovopsis* (Augustin et al. 2013). On the other hand, *Sympodiorosea* and *Luteomyces* are the only genera within the *Hypocreaceae* that present sympodial and poorly differentiated holoblastic conidiogenous cells, respectively. Interestingly, only a distant group of entomopathogenic fungi, i.e., *Beauveria* (*Ascomycota*: *Hypocreales*, *Cordycipitaceae*), has sympodial conidiogenesis (Rehner et al. 2011) like *Sympodiorosea*, and there are no other groups of fungi within the *Hypocreaceae* that form poorly differentiated holoblastic conidiogenous cells like *Luteomyces*. Future studies will hopefully shed light on the evolutionary pressures that led *Escovopsis*, *Sympodiorosea*, and *Luteomyces* to form these unique microscopic characters.

The consideration of all fungi producing brown conidia in the attine ant’s colonies as *Escovopsis* made of this genus polyphyletic and paraphyletic (Fig. 1, Additional file 3: Fig. S2). Lack of a comprehensive phylogenetic analysis has precluded resolving the phylogenetic uncertainties of *Escovopsis* (Montoya et al. 2019). In light of our results, we considered two hypotheses to solve the phylogenetic disagreements of this group of fungi: first, *Escovopsis*, *Luteomyces*, and *Sympodiorosea* belong to the same genus. In this case, *Escovopsioides* (sister clade of *Luteomyces*, Fig. 3A, F) and *Hypomyces* (closely related with *Sympodiorosea* and *Escovopsioides*, Fig. 3C, D) would have to belong to the same genus to enforce monophyly (Baum and Smith, 2013). However, both *Escovopsioides* and *Hypomyces* are well supported, separate monophyletic clades (Figs. 1, 2, 3) and present unique morphological characters that differentiate them from other genera in the *Hypocreaceae*. *Escovopsioides*, for instance, is the only genus within the *Hypocreaceae* that forms phialides on tiny intercalary vesicles on the aerial mycelium. In contrast, many species of *Hypomyces* forms septate conidia and some species form sexual structures (despite being a physiological or genetic character not easily observed within the group), which are not observed in *Escovopsis*, *Luteomyces*, *Sympodiorosea* or *Escovopsioides*. Second, *Escovopsis*, *Luteomyces*, and *Sympodiorosea* represent taxa within different genera. Moreover, while different, *Escovopsioides* and *Hypomyces* (some species) are the only genera within the *Hypocreaceae* that form plural types of conidia (i.e., one type of conidia from phialides and another direct from aerial mycelia without conidiogenous cells (Põldmaa et al. 1999; Augustin et al. 2013). In this case, considering that: (1) the same genes, in different genera, follow different evolutionary paths (Gompel and Prud’homme 2009), and (2) regardless of the molecular markers used in this study, each clade preserves its monophyly within the *Hypocreaceae*; the variation of the phylogenetic position of the three clades (Fig. 3) could be better explained if they are different genera. In light of this evidence, the combination of morphological and phylogenetic data, using various molecular markers in a multilocus analysis (Taylor et al. 2000) suggests the second hypothesis as the most parsimonious. Future research, using genome-based phylogenetic methods, may resolve the relationship of these genera to one another.

The circumscription of *Escovopsis* raises important questions for the genus. How diverse is *Escovopsis*? What is its host range? How is the genus phylogeographically distributed? And, what is its role in attine gardens? For many years, *Escovopsis* was considered a diverse group of fungi (Gerardo et al. 2006a, b; Rodrigues et al. 2008, 2011; Caldera et al. 2009; Pagnocca et al. 2012; Yek et al. 2012; Meirelles et al. 2015a, b). However, that assumption was based on considering *Sympodiorosea* spp., *L. trichodermoides* and the clades C and D (putative new genera; Fig. 1, Additional file 3: Fig. S2) within *Escovopsis*. Currently, *E. aspergilloides*, *E. clavata*, *E. lentecrescens*, *E. microspora*, *E. moelleri*, *E. multiformis,* and *E. weberi* are the only species formaly described within the genus *Escovopsis* analysed using a multilocus approach based on five molecular markers (Fig. 3G). The species introduced by Marfetán et al. (2018) also belong to *Escovopsis* (Additional file 2: Fig. S1), nonetheless, the phylogeny of these species are still unclear. Therefore, future studies should consider that both the genetic diversity and the number of known species of the genus were overestimated. Since the taxonomic conditions to evaluate the macroscopic features and growth rates of *Escovopsis* species are still not standardized, the assessment to the morphological diversity of the genus and description of new species are still limited. Accordingly, future studies should access the morphology of the formally described *Escovopsis* species to create a standardized taxonomic framework of the genus, and strengthen the foundations of its systematics. Finally, several studies provided evidence that some *Escovopsis* species act as specialized mycoparasite (Currie et al. 1999a, 2003; Currie 2001; Gerardo et al. 2004; Little and Currie 2007; de Man et al. 2016). However, a recent study showed that *Escovopsis* species may act as an opportunistic fungus in attine ant colonies depending on host susceptibility (Jiménez-Gómez et al. 2021). Either way, the parasitic mechanisms of the *Escovopsis* species that have a parasitic behaviour are still poorly understood, and the hypothesis of specialized mycoparasite for the genus was also raised considering *Sympodiorosea* spp., *L. trichodermoides* and the clades C and D (Fig. 1 and Additional file 3: Fig. S2) within the same genus. Therefore, future studies should unveil the phylogenetic correspondence of *Escovopsis* with the ants and the mutualistic fungi, and carefully address the mechanisms of the parasitism of this group of fungi.

Similar questions as those raised for *Escovopsis* must be addressed in future studies for *Sympodiorosea*, *Luteomyces* and the clades C and D. The genetic and morphological diversity, as well as the geographical distribution of *Sympodiorosea*, *Luteomyces* and the two putative new genera (Fig. 1 and Additional file 3: Fig. S2), are still a mystery. Some authors suggested that *Sympodiorosea* spp. and *L. trichodermoides* are more likely to be associated with the colonies of lower attine ants than higher attine (e.g., leaf-cutting) ants (Gerardo et al. 2004, 2006b), but more evidence is necessary to confirm this hypothesis. Recent studies have shown that some strains of *S. kreiselii* behave as antagonists of the mutualistic fungus of *Mycetophylax morschi* being able to kill it under laboratory conditions (Custodio and Rodrigues 2019). However, the mechanisms by which it manages to kill the cultivars are completely unknown. On the other hand, Bizarria et al. (2020) demonstrated that *L. trichodermoides* had little negative impact on the mutualistic fungus of *Mycocepurus goeldii*, being just able to inhibit the fungus cultivars in-vitro and unable to overcome defenses of the ant colonies. The taxonomy, ecology, and lifestyle of the clades C and D (Fig. 1 and Additional file 3: Fig. S2) previously treated as *Escovopsis* are still unknown. Therefore, all assumptions about these groups of fungi should be reconsidered and examined carefully in future studies.

## Conclusion

Since Möller (1893) observed the “fungi of the strong conidial shapes” within the fungus gardens of fungus-growing ants, several groups of fungi that share the same habitat were classified as *Escovopsis*. Many phylogenetic incongruities have been reported in the last two decades, and the lack of phylogenetic studies for this genus has hampered scientists to recognize the root of the problem. After a detailed systematic study, we conclude that taxonomic disagreements in the genus *Escovopsis* were caused due to the inclusion of two groups of fungi that belong to different genera (*Luteomyces* and *Sympodiorosea*) within the same genus. This discovery not only solves the phylogenetic disagreements of the genus but significantly expands our understanding of the systematics of *Escovopsis*, and related genera, and provides a stable foundation from which to build future research on the evolutionary history, taxonomic diversity, and ecological roles of these unique fungi.

## Supplementary Information

**Additional file 1.Table S1**. Strains and their associated metadata used in the phylogenetic analyses at family level (Fig. 3). From these, 64 strains are from *Escovopsis* spp., 24 strains are from *Luteomyces trichodermoides*, 14 strains are from *Sympodiorosea* spp., 30 strains are from five *Hypocreaceae* genera [*Escovopsioides*, *Hypomyces* (along with species under its anamorphic genus *Cladobotryum*), *Protocrea*, *Sphaerostilbella*, and *Trichoderma*], and *Lecanicillium antillanum* CBS 350.85 was used as the outgroup. **Table S2**. Molecular markers, primers and polymerase chain reaction conditions used in this study. **Table S3**. Sequences and their associated metadata used to show the phylogenetic placement of all strains previously named as *Escovopsis *(Figs. 1 and S2). This table contains a total of 440* tef*1 sequences from 274 strains from vesiculate-*Escovopsis* spp., 105 strains from non-vesiculate *Escovopsis* [24 strains from *Luteomyces trichodermoides* (previously introduced as “*Escovopsis trichodermoides*”), 57 strains from *Sympodiorosea* spp. (9 *Sympodiorosea kreiselii* previously introduced as “*Escovopsis kreiselii*”, 5 *Sympodiorosea* spp. introduced in this study, 6 *Sympodiorosea* spp. previously introduced as “*Escovopsis*”, 37 *Sympodiorosea* spp. previously introduced as “Pink *Escovopsis*”), 24 strains labeled as “?” (because they form two new clades that likely correspond to new genera 20 previously introduced as “White *Escovopsis*”, 2 as “Yellow *Escovopsis*”, and 2 as “*Escovopsis*”)], 60 strains from five *Hypocreaceae* genera [*Escovopsioides*, *Hypomyces* (along with species under its anamorphic genus *Cladobotryum*), *Protocrea*, *Sphaerostilbella*, and *Trichoderma*], and *Lecanicillium antillanum *CBS 350.85 as the outgroup. **Table S4**. Strains and their associated metadata used to show the phylogenetic placement of *Escovopsis* species described by Marfetán et al. (2018) (Fig. S1). **Table S5**. Strains of the *Hypocreales* and their metadata used in the phylogenetic analysis at order-level (Fig. 2).

**Additional file 2: Figure S1.** Phylogenetic placement of *Escovopsis* species described by Marfetán et al. (2018). The phylogenetic tree was reconstructed to include the LSU sequences (in the green box on the tree) generated by Marfetán et al. (2018). The tree was performed using Maximum Likelihood (ML) in RAxML v.8 (Stamatakis 2014) and Bayesian Inference (BI) in MrBayes v.3.2.2 (Ronquist et al. 2012) using the GTR model. For ML analyses, 1000 independent trees and 1000 bootstrap replicates were generated. For BI analyses, two million generations of the Markov Chain Monte Carlo were enough to reach convergence. Numbers on branches indicate BI posterior probabilities (PP) and ML bootstrap support values (MLB), respectively. Hyphens (--) indicate MLB < 70%. *Lecanicillium antillanum* CBS 350.85 was used as the outgroup. Four species described by Marfetán et al. (2018) (*Escovopsis atlas*, *E. catenulata*, *E. pseudoweberi*, and *E. primorosea*) formed two clades (green box) within *Escovopsis* close to *E. aspergilloides* and *E. lentecrescens*. *Escovopsis atlas*, *E. catenulata*, *E. pseudoweberi* were placed into the same clade along with five strains identified as *E. weberi* (distant from the type of *E. weberi*) and four strains of *E. primorosea* formed a monophyletic clade. *Escovopsis longivesica* was not included in this tree because the LSU sequences of this species do not have similarity with *Escovopsis* but with *Ceriporia alachuana* (95.4% identity for *E. longivesica* E5 and *E. longivesica* E9) and *Penicillium glabrum* (95.3% identity for *E. longivesica* E10). See Additional file 1: Table S4 for all strains and their associated metadata used to infer this phylogenetic tree.

**Additional file 3: Figure S2.** Extended phylogenetic tree (from Fig. 1) indicating the placement of every isolate previously treated as *Escovopsis*. The tree shown was inferred using Bayesian Inference (BI). The tree gathers all available *tef*1 sequences found in the literature and the data set used in this study, including the sequences of the nine *Escovopsis* ex-type cultures. The tree contains a total of 440 sequences which include: 274 strains of vesiculate-*Escovopsis* (Clade A), 105 strains of non-vesiculate *Escovopsis* (Clades B, C, D, E) and 60 strains from four genera, i.e., *Escovopsioides*, *Hypomyces* (along with species under its anamorphic genus *Cladobotryum*), *Protocrea*, and *Trichoderma*, in the *Hypocreaceae*. *Lecanicillium antillanum* CBS 350.85 was used as the outgroup. Numbers on branches indicate BI posterior probabilities (PP) and Maximum Likelihood bootstrap support values (MLB), respectively. Hyphens (--) indicate MLB < 70%. There is only information, in the literature, on the colour of the colonies of the clades C and D, but the microscopic features of these clades are unknown. See Additional file 1: Table S3 for all strains and their associated metadata used to infer this phylogenetic tree.

## Data Availability

The datasets used in this study are available in the NCBI-GenBank (Additional file 1: Tables S1, S3, S4, and S5). The alignments and their respective phylogenetic trees generated in this study are available in TreeBASE (http://purl.org/phylo/treebase/phylows/study/TB2:S28662).

## Adherence to national and international regulations

We followed all the requirements for specimen acquisition, transportation and study according to the Nagoya Protocol to the Convention on Biological Diversity 2011.
