# Supplementary material for: Fungi inhabiting attine ant colonies: reassessment of the genus Escovopsis and description of Luteomyces and Sympodiorosea gens. nov
Source: IMA Fungus. 2021 Aug 24;12:23. doi: 10.1186/s43008-021-00078-8 (PMC8383443; doi:10.1186/s43008-021-00078-8)
Supplement: Supplementary file 1 — Additional file 1.Table S1. Strains and their associated metadata used in the phylogenetic analyses at family level (Fig. 3). From these, 64 strains are from Escovopsis spp., 24 strains are from Luteomyces trichodermoides, 14 strains are from Sympodiorosea spp., 30 strains are from five Hypocreaceae genera [Escovopsioides, Hypomyces (along with species under its anamorphic genus Cladobotryum), Protocrea, Sphaerostilbella, and Trichoderma], and Lecanicillium antillanum CBS 350.85 was used as the outgroup. Table S2. Molecular markers, primers and polymerase chain reaction conditions used in this study. Table S3. Sequences and their associated metadata used to show the phylogenetic placement of all strains previously named as Escovopsis (Figs. 1 and S2). This table contains a total of 440 tef1 sequences from 274 strains from vesiculate-Escovopsis spp., 105 strains from non-vesiculate Escovopsis [24 strains from Luteomyces trichodermoides (previously introduced as “Escovopsis trichodermoides”), 57 strains from Sympodiorosea spp. (9 Sympodiorosea kreiselii previously introduced as “Escovopsis kreiselii”, 5 Sympodiorosea spp. introduced in this study, 6 Sympodiorosea spp. previously introduced as “Escovopsis”, 37 Sympodiorosea spp. previously introduced as “Pink Escovopsis”), 24 strains labeled as “?” (because they form two new clades that likely correspond to new genera 20 previously introduced as “White Escovopsis”, 2 as “Yellow Escovopsis”, and 2 as “Escovopsis”)], 60 strains from five Hypocreaceae genera [Escovopsioides, Hypomyces (along with species under its anamorphic genus Cladobotryum), Protocrea, Sphaerostilbella, and Trichoderma], and Lecanicillium antillanum CBS 350.85 as the outgroup. Table S4. Strains and their associated metadata used to show the phylogenetic placement of Escovopsis species described by Marfetán et al. (2018) (Fig. S1). Table S5. Strains of the Hypocreales and their metadata used in the phylogenetic analysis at order-level (Fig. 2). [file 43008_2021_78_MOESM1_ESM.docx]

**Supplementary material**

**Fungi inhabiting attine ant colonies: reassessment of the genus *Escovopsis* and description of *Luteomyces* and *Sympodiorosea* gens. nov.**

Quimi Vidaurre Montoya, Maria Jesus Sutta Martiarena, Rodolfo Bizarria Jr., Nicole Marie Gerardo, Andre Rodrigues

Table S1. Strains and their associated metadata used in the phylogenetic analyses at family level (Fig. 3). From these, 64 strains are from *Escovopsis* spp., 24 strains are from *Luteomyces trichodermoides*, 14 strains are from *Sympodiorosea* spp., 30 strains are from five *Hypocreaceae* genera [*Escovopsioides*, *Hypomyces* (along with species under its anamorphic genus *Cladobotryum*)*, Protocrea*, *Sphaerostilbella*, and *Trichoderm*a], and *Lecanicillium antillanum* CBS 350.85 was used as the outgroup.

| **Previous fungal species name** | **Current fungal species name** | **Strain ID** | **Specimen voucher** | **City, State, Country** | **Geographical coordinates** | **Habitat** | **GenBank accessions** | | | | | **References** |
| --- | --- | --- | --- | --- | --- | --- | --- | --- | --- | --- | --- | --- |
|  |  |  |  |  |  |  | **ITS** | **LSU** | ***tef*1** | ***rpb*1** | ***rpb*2** |  |
| *Escovopsioides nivea* | *Escovopsioides nivea* | CBS 135749^ET^ | AUJ6 | Viçosa, Minas Gerais, Brazil | 20°44'31.71''S; 42°52 '43.83''W | Fungus garden of *Acromyrmex subterraneus subterraneus* | JQ815078 | JQ855716 | JQ855713 | MT305414^#^ | MT305539^#^ | Augustin et al. (2013) |
| *Escovopsioides* sp. | *Escovopsioides nivea* | LESF 159 | J08 | Corumbataí, São Paulo, Brazil | 22°17'21.7''S; 47°39'22.8''W | Fungus garden of *Atta sexdens rubropilosa* | MF116014 | MF116034 | MF140949 | MT305444^#^ | MT305569^#^ | Osti and Rodrigues (2018) |
| *Escovopsioides* sp. | *Escovopsioides nivea* | LESF 601 | J09 | Rio Claro, São Paulo, Brazil |  | Fungus garden of *Trachymyrmex* sp. | MF116029 | MF116049 | MF140964 | MT305487^#^ | MT305612^#^ | Osti and Rodrigues (2018) |
| *Escovopsioides* sp. | *Escovopsioides nivea* | LESF 151 | J02 | Corumbataí, São Paulo, Brazil | 22°17'21.7''S; 47°39'22.8''W | Fungus garden of *Atta sexdens rubropilosa* | MF116013 | MF116033 | MF140948 | MT305442^#^ | MT305567^#^ | Osti and Rodrigues (2018) |
| *Escovopsioides* sp. | *Escovopsioides nivea* | LESF 510 | J10 | Botucatu, São Paulo, Brazil | 22°54'28.4"S; 48°18'55.7"W | Fungus garden of *Atta sexdens rubropilosa* | MF116015 | MF116035 | MF140950 | MT305474^#^ | MT305599^#^ | Osti and Rodrigues (2018) |
| *Escovopsioides* sp. | *Escovopsioides nivea* | LESF 587 | J01 | Camacan, Bahia, Brazil | 15°23'18.2"S; 39°33'30.5"W | Fungus garden of *Atta cephalotes* | MF116016 | MF116036 | MF140951 | MT305477^#^ | MT305602^#^ | Osti and Rodrigues (2018) |
| *Escovopsioides* sp. | *Escovopsioides nivea* | LESF 588 | J03 | Camacan, Bahia, Brazil | 15°25'32.3"S 39°32'48.1"W | Fungus garden of *Atta cephalotes* | MF116017 | MF116037 | MF140952 | MT305478^#^ | MT305603^#^ | Osti and Rodrigues (2018) |
| *Escovopsioides* sp. | *Escovopsioides nivea* | LESF 589 | J05 | Camacan, Bahia, Brazil | 15°23'14.8"S; 39°33'28.4"W | Fungus garden of *Atta cephalotes* | MF116018 | MF116038 | MF140953 | MT305479^#^ | MT305604^#^ | Osti and Rodrigues (2018) |
| *Escovopsioides* sp. | *Escovopsioides nivea* | LESF 590 | J06 | Camacan, Bahia, Brazil | 15°23'15.2S; 39°33'28.0"W | Fungus garden of *Atta cephalotes* | MF116019 | MF116039 | MF140954 | MT305480^#^ | MT305605^#^ | Osti and Rodrigues (2018) |
| *Escovopsioides* sp. | *Escovopsioides nivea* | LESF 591 | J04 | Botucatu, São Paulo, Brazil | 22°54'26.6"S; 48°18'29.2"W | Fungus garden of *Atta capiguara* | MF116020 | MF116040 | MF140955 | MT305481^#^ | MT305606^#^ | Osti and Rodrigues (2018) |
| *Escovopsioides* sp. | *Escovopsioides nivea* | LESF 592 | J12 | Camacan, Bahia, Brazil | 15°22'50.3''S; 39°34'03.5''W | Fungus garden of *Acromyrmex* sp. | MF116021 | MF116041 | MF140956 | MT305482^#^ | MT305607^#^ | Osti and Rodrigues (2018) |
| *Escovopsioides* sp. | *Escovopsioides nivea* | LESF 596 | J16 | Chuvisca, Rio Grande do Sul, Brazil | 30°50'10.2"S; 51°55'10.4"W | Fungus garden of *Acromyrmex* sp. | MF116025 | MF116045 | MF140960 | MT305483^#^ | MT305608^#^ | Osti and Rodrigues (2018) |
| *Escovopsioides* sp. | *Escovopsioides nivea* | LESF 597 | J17 | Camacan, Bahia, Brazil | 15°23'29.7"S; 39°33'31.3"W | Fungus garden of *Atta cephalotes* | MF116026 | MF116046 | MF140961 | MT305484^#^ | MT305609^#^ | Osti and Rodrigues (2018) |
| *Escovopsioides* sp. | *Escovopsioides nivea* | LESF 598 | J18 | Camacan, Bahia, Brazil | 15°23'17.8"S; 39 33'22.3"W | Fungus garden of *Atta cephalotes* | MF116027 | MF116047 | MF140962 | MT305485^#^ | MT305610^#^ | Osti and Rodrigues (2018) |
| *Escovopsioides* sp. | *Escovopsioides nivea* | LESF 599 | J19 | Sentinela do Sul, Rio Grande do Sul, Brazil |  | Fungus garden of *Acromyrmex heyeri* | MF116028 | MF116048 | MF140963 | MT305486^#^ | MT305611^#^ | Osti and Rodrigues (2018) |
| *Escovopsioides* sp. | *Escovopsioides nivea* | LESF 1009 | QVM137 | Manaus, Amazonas, Brazil | 2°26'55.3''S; 59°46'10.9''W | Fungus garden of *Apterostigma* sp. | MT273483 | MT273572 | MT305392 | MT305516 | MT305641 | This study |
| *Escovopsioides* sp. | *Escovopsioides nivea* | LESF 1023 | QVM151 | Manaus, Amazonas, Brazil | 2°26'52.5''S; 59°45'53.4''W | Fungus garden of *Trachymyrmex* sp. | MT273485 | MT273574 | MT305394 | MT305518 | MT305643 | This study |
| *Escovopsioides* sp. | *Escovopsioides nivea* | LESF 1025 | QVM153 | Manaus, Amazonas, Brazil | 2°26'52.5''S; 59°45'53.4''W | Fungus garden of *Trachymyrmex* sp. | MT273486 | MT273575 | MT305395 | MT305519 | MT305644 | This study |
| *Escovopsioides* sp. | *Escovopsioides nivea* | LESF 1028 | QVM156 | Manaus, Amazonas, Brazil | 2°26'52.6''S; 59°45'52.4''W | Fungus garden of *Trachymyrmex* sp. | MT273487 | MT273576 | MT305396 | MT305520 | MT305645 | This study |
| *Escovopsioides* sp. | *Escovopsioides nivea* | LESF 1031 | QVM159 | Manaus, Amazonas, Brazil | 2°26'55.5''S; 59°45'54.2''W | Fungus garden of *Trachymyrmex* sp. | MT273488 | MT273577 | MT305397 | MT305521 | MT305646 | This study |
| *Escovopsioides* sp. | *Escovopsioides nivea* | LESF 1039 | QVM167 | Manaus, Amazonas, Brazil | 2°26'52.3''S; 59° 45'51.9''W | Fungus garden of *Cyphomyrmex* sp*.* | MT273489 | MT273578 | MT305398 | MT305522 | MT305647 | This study |
| *Escovopsioides* sp. | *Escovopsioides nivea* | LESF 1040 | QVM168 | Manaus, Amazonas, Brazil | 2°26'52.6''S; 59°45'52.4''W | Fungus garden of *Apterostigma* sp. | MT273490 | MT273579 | MT305399 | MT305523 | MT305648 | This study |
| *Escovopsioides* sp. | *Escovopsioides nivea* | LESF 1041 | QVM169 | Manaus, Amazonas, Brazil | 2°26'55.5''S; 59°45'54.2''W | Fungus garden of *Apterostigma* sp. | MT273491 | MT273580 | MT305400 | MT305524 | MT305649 | This study |
| *Escovopsis* *aspergilloides* | *Escovopsis* *aspergilloides* | CBS 423.93 ^ET^ | DAOM:216382 | Trinidad and Tobago: Trinidad |  | Fungus garden of *Trachymyrmex ruthae* | NR_137160 | KF293283 | AY172632 | MT305421^#^ | MT305546^#^ | Augustin et al. (2013),  Currie et al. (2003) |
| *Escovopsis clavata* | *Escovopsis clavata* | LESF 854 | 1704A | Florianópolis, Santa Catarina, Brazil | 27°44'38.94''S 48°31'9.3''W | Fungus garden of *Apterostigma* sp. | MH715097 | MH715111 | MH724271 | MT305495^#^ | MT305620^#^ | Montoya et al. (2019) |
| *Escovopsis clavata* | *Escovopsis clavata* | LESF 855 | 1705B | Florianópolis, Santa Catarina, Brazil | 27°44'39.49''S 48°31'9.72''W | Fungus garden of *Apterostigma* sp. | MH715098 | MH715112 | MH724272 | MT305496^#^ | MT305621^#^ | Montoya et al. (2019) |
| *Escovopsis clavata* | *Escovopsis clavata* | CBS 145326 ^ET^ | 1707 | Florianópolis, Santa Catarina, Brazil | 27°44'39.6''S 48°31'10.14''W | Fungus garden of *Apterostigma* sp. | MH715096 | MH715110 | MH724270 | MT305419^#^ | MT305544^#^ | Montoya et al. (2019) |
| *Escovopsis kreiselii* | *Sympodiorosea kreiselii* | LESF 302 | AR14022705 | Florianópolis, Santa Catarina, Brazil | 27°31'24.96''S  48°25'3.78''W | Fungus garden of *Mycetophylax morschi* | MH715085 | MH715099 | MH724259 | MT305452^#^ | MT305577^#^ | Montoya et al. (2019) |
| *Escovopsis kreiselii* | *Sympodiorosea kreiselii* | LESF 303 | AR14022705B | Florianópolis, Santa Catarina, Brazil | 27°31'24.96''S  48°25'3.78''W | Fungus garden of *Mycetophylax morschi* | MH715086 | MH715100 | MH724260 | MT305453^#^ | MT305578^#^ | Montoya et al. (2019) |
| *Escovopsis kreiselii* | *Sympodiorosea kreiselii* | LESF 304 | AR14022705T2D | Florianópolis, Santa Catarina, Brazil | 27°31'24.96''S  48°25'3.78''W | Fungus garden of *Mycetophylax morschi* | MH715087 | MH715101 | MH724261 | MT305454^#^ | MT305579^#^ | Montoya et al. (2019) |
| *Escovopsis kreiselii* | *Sympodiorosea kreiselii* | LESF 305 | AR14022601 | Florianópolis, Santa Catarina, Brazil | 27°37'49.6"S; 48°27'03.6"W | Fungus garden of *Mycetophylax morschi* | MT273438 | MT273524 | MT305353 | MT305455 | MT305580 | This study |
| *Escovopsis kreiselii* | *Sympodiorosea kreiselii* | LESF 306 | AR14022705A | Florianópolis, Santa Catarina, Brazil | 27°31'25.0"S; 48°25'03.8"W | Fungus garden of *Mycetophylax morschi* | MT273439 | MT273525 | MT305354 | MT305456 | MT305581 | This study |
| *Escovopsis kreiselii* | *Sympodiorosea kreiselii* | LESF 307 | AR14022705T2 | Florianópolis, Santa Catarina, Brazil | 27°31'25.0"S; 48°25'03.8"W | Fungus garden of *Mycetophylax morschi* | MT273440 | MT273526 | MT305355 | MT305457 | MT305582 | This study |
| *Escovopsis kreiselii* | *Sympodiorosea kreiselii* | LESF 308 | AR14022604AL | Florianópolis, Santa Catarina, Brazil | 27°37'49.6"S; 48°27'03.6"W | Fungus garden of *Mycetophylax morschi* | MT273441 | MT273527 | MT305356 | MT305458 | MT305583 | This study |
| *Escovopsis kreiselii* | *Sympodiorosea kreiselii* | LESF 309 | AR14022605T2 | Florianópolis, Santa Catarina, Brazil | 27°37'47.9"S; 48°27'04.0"W | Fungus garden of *Mycetophylax morschi* | MT273442 | MT273528 | MT305357 | MT305459 | MT305584^#^ | This study |
| *Escovopsis kreiselii* | *Sympodiorosea kreiselii* | CBS 139320 ^ET^ | LESF 053 | Florianópolis, Santa Catarina, Brazil | 27°37'50.01''S 48°27'03.64''W | Fungus garden of *Mycetophylax morschi* | KJ808767 | KJ808765 | KJ 808766 | MT305418^#^ | MT305543^#^ | Meirelles et al. (2015a) |
| *Escovopsis lentecrescens* | *Escovopsis lentecrescens* | CBS 135750 ^ET^ | VIC:31755 | Viçosa, Minas Gerais, Brazil | 20°44'31.71''S 42°52'43.83''W | Fungus garden of *Acromyrmex subterraneus subterraneus* | JQ815079 | JQ855717 | JQ855714 | MT305415^#^ | MT305540^#^ | Augustin et al. (2013) |
| *Escovopsis microspora* | *Escovopsis microspora* | CBS 135751^ET^ | VIC:31756 | Viçosa, Minas Gerais, Brazil | 20°44'31.71''S; 42°52'43.83''W | Fungus garden of *Acromyrmex subterraneus molestans* | JQ815076 | KF293284 | KJ935030^&^ | MT305416^#^ | MT305541^#^ | Augustin et al. (2013),  Meirelles et al. (2015a) |
| *Escovopsis moelleri* | *Escovopsis moelleri* | CBS 135748 ^ET^ | VIC:31753 | Viçosa, Minas Gerais, Brazil | 20°44'31.71''S 42°52'43.83''W | Fungus garden of *Acromyrmex subterraneus molestans* | JQ815077 | JQ855715 | JQ855712 | MT305413^#^ | MT305538^#^ | Augustin et al. (2013) |
| *Escovopsis multiformis* | *Escovopsis multiformis* | LESF 1136 | QVM277 | Alta Floresta, Mato Grosso, Brazil | 09°49'22.7''S 58°15'32.0''W | Fungus garden of *Apterostigma* sp. | MH715092 | MH715106 | MH724266 | MT305536^#^ | MT305661^#^ | Montoya et al. (2019) |
| *Escovopsis multiformis* | *Escovopsis multiformis* | CBS 145327 ^ET^ | 1606w | Florianópolis, Santa Catarina, Brazil | 27°28'11.28''S 48°22'39.48''W | Fungus garden of *Apterostigma* sp. | MH715091 | MH715105 | MH724265 | MT305420^#^ | MT305545^#^ | Montoya et al. (2019) |
| *Escovopsis* sp. | *Escovopsis multiformis* | LESF 852 | 1706B | Florianópolis, Santa Catarina, Brazil | 27°44'39.4''S; 48°31'10.0''W | Fungus garden of *Apterostigma* sp. | MT273460 | MT273549 | MT305372 | MT305494 | MT305619 | This study |
| *Escovopsis trichodermoides* | *Luteomyces trichodermoides* | LESF 310 | AR14022604A1 | Florianópolis, Santa Catarina, Brazil | 27°37'49.62''S, 48°27'3.6''W | Fungus garden of *Mycetophylax morschi* | MH715088 | MH715102 | MH724262 | MT305460^#^ | MT305585^#^ | Montoya et al. (2019) |
| *Escovopsis trichodermoides* | *Luteomyces trichodermoides* | LESF 311 | AR14022604A2 | Florianópolis, Santa Catarina, Brazil | 27°37'49.62''S, 48°27'3.6''W | Fungus garden of *Mycetophylax morschi* | MH715089 | MH715103 | MH724263 | MT305461^#^ | MT305586^#^ | Montoya et al. (2019) |
| *Escovopsis trichodermoides* | *Luteomyces trichodermoides* | LESF 312 | AR14022604ALA | Florianópolis, Santa Catarina, Brazil | 27°37'49.62''S, 48°27'3.6''W | Fungus garden of *Mycetophylax morschi* | MH715090 | MH715104 | MH724264 | MT305462^#^ | MT305587^#^ | Montoya et al. (2019) |
| *Escovopsis trichodermoides* | *Luteomyces trichodermoides* | LESF 832 | 13I3 | Rio Claro, São Paulo, Brazil |  | Fungus garden of *Mycocepurus smithii* | MT273453 | MT273542 | MT305366 | MT305488 | MT305613 | This study |
| *Escovopsis trichodermoides* | *Luteomyces trichodermoides* | LESF 833 | 13I1 | Rio Claro, São Paulo, Brazil |  | Fungus garden of *Mycocepurus smithii* | MT273454 | MT273543 | MT305367 | MT305489 | MT305614 | This study |
| *Escovopsis trichodermoides* | *Luteomyces trichodermoides* | LESF 834 | 13I2 | Rio Claro, São Paulo, Brazil |  | Fungus garden of *Mycocepurus smithii* | MT273455 | MT273544 | MT305368 | MT305490 | MT305615 | This study |
| *Escovopsis trichodermoides* | *Luteomyces trichodermoides* | LESF 835 | 3I1 | Rio Claro, São Paulo, Brazil |  | Midden of *Mycocepurus smithii* | MT273456 | MT273545 | MT305369 | MT305491 | MT305616 | This study |
| *Escovopsis trichodermoides* | *Luteomyces trichodermoides* | LESF 837 | 2I2 | Rio Claro, São Paulo, Brazil |  | Fungus garden of *Mycocepurus smithii* | MT273458 | MT273547 | MT305370 | MT305492 | MT305617 | This study |
| *Escovopsis trichodermoides* | *Luteomyces trichodermoides* | LESF 838 | 2I3 | Rio Claro, São Paulo, Brazil |  | Fungus garden of *Mycocepurus smithii* | MT273459 | MT273548 | MT305371 | MT305493 | MT305618 | This study |
| *Escovopsis trichodermoides* | *Luteomyces trichodermoides* | LESF 895 | Q03I | Botucatu, São Paulo, Brazil | 22°54'19.6''S; 48°14' 33.7''W | Fungus garden of *Mycocepurus goeldii* | MT273470 | MT273559 | MT305380 | MT305504 | MT305629 | This study |
| *Escovopsis* sp. | *Luteomyces trichodermoides* | LESF 897 | Q03III | Botucatu, São Paulo, Brazil | 22°54'19.6''S; 48°14'33.7''W | Fungus garden of *Mycocepurus goeldii* | MT273472 | MT273561 | MT305381 | MT305505 | MT305630 | This study |
| *Escovopsis trichodermoides* | *Luteomyces trichodermoides* | LESF 927 | Q23III | Botucatu, São Paulo, Brazil | 22° 54' 41.7''S; 48°14' 49.5'' NMG | Fungus garden of *Mycocepurus goeldii* | MT273474 | MT273563 | MT305383 | MT305507 | MT305632 | This study |
| *Escovopsis trichodermoides* | *Luteomyces trichodermoides* | LESF 1049 | QVM177 | Botucatu, São Paulo, Brazil | 22°54'20.6''S; 48°14'34.2''W | Fungus garden of *Mycocepurus goeldii* | MT273492 | MT273581 | MT305401 | MT305525 | MT305650 | This study |
| *Escovopsis trichodermoides* | *Luteomyces trichodermoides* | LESF 1051 | QVM179 | Botucatu, São Paulo, Brazil | 22°54'19.8''S; 48°14'33.6''W | Fungus garden of *Mycocepurus goeldii* | MT273493 | MT273582 | MT305402 | MT305526 | MT305651 | This study |
| *Escovopsis trichodermoides* | *Luteomyces trichodermoides* | LESF 1052 | QVM180 | Botucatu, São Paulo, Brazil | 22°54'19.8''S; 48°14'33.6''W | Fungus garden of *Mycocepurus goeldii* | MT273494 | MT273583 | MT305403 | MT305527 | MT305652 | This study |
| *Escovopsis trichodermoides* | *Luteomyces trichodermoides* | LESF 1055 | QVM183 | Botucatu, São Paulo, Brazil | 22°54'19.6''S; 48°14'33.6''W | Fungus garden of *Mycocepurus goeldii* | MT273495 | MT273584 | MT305404 | MT305528 | MT305653 | This study |
| *Escovopsis trichodermoides* | *Luteomyces trichodermoides* | LESF 1057 | QVM185 | Botucatu, São Paulo, Brazil | 22°54'18.8''S; 48°14'33.3''W | Fungus garden of *Mycocepurus goeldii* | MT273496 | MT273585 | MT305405 | MT305529 | MT305654 | This study |
| *Escovopsis trichodermoides* | *Luteomyces trichodermoides* | LESF 1061 | QVM189 | Botucatu, São Paulo, Brazil | 22°54'19.5''S; 48°14'32.7''W | Fungus garden of *Mycocepurus goeldii* | MT273497 | MT273586 | MT305406 | MT305530 | MT305655 | This study |
| *Escovopsis trichodermoides* | *Luteomyces trichodermoides* | LESF 1077 | QVM205 | Botucatu, São Paulo, Brazil | 22°54'19.2''S; 48°14'32.3''W | Fungus garden of *Mycocepurus goeldii* | MT273498 | MT273587 | MT305407 | MT305531 | MT305656 | This study |
| *Escovopsis trichodermoides* | *Luteomyces trichodermoides* | LESF 1078 | QVM206 | Botucatu, São Paulo, Brazil | 22°54'19.2''S; 48°14'32.3''W | Fungus garden of *Mycocepurus goeldii* | MT273499 | MT273588 | MT305408 | MT305532 | MT305657 | This study |
| *Escovopsis trichodermoides* | *Luteomyces trichodermoides* | LESF 1082 | QVM210 | Botucatu, São Paulo, Brazil | 22°54'19.2''S; 48°14'32.3''W | Fungus garden of *Mycocepurus goeldii* | MT273500 | MT273589 | MT305409 | MT305533 | MT305658 | This study |
| *Escovopsis trichodermoides* | *Luteomyces trichodermoides* | LESF 1090 | QVM218 | Botucatu, São Paulo, Brazil | 22°54'16.7''S; 48°14'31.3''W | Fungus garden of *Mycocepurus goeldii* | MT273501 | MT273590 | MT305410 | MT305534 | MT305659 | This study |
| *Escovopsis trichodermoides* | *Luteomyces trichodermoides* | LESF 1109 | QVM237 | Botucatu, São Paulo, Brazil | 22°54'17.9''S; 48°14'33.0''W | Fungus garden of *Mycocepurus goeldii* | MT273502 | MT273591 | MT305411 | MT305535 | MT305660 | This study |
| *Escovopsis trichodermoides* | *Luteomyces trichodermoides* | CBS 137343 ^ET^ | VEM001 | Rio Claro, São paulo, Brazil | 22°23'46.93''S, 47°32'40.12''W | Fungus garden of *Mycocepurus goeldii* | KJ485699 | MF116052^$^ | KF033128 | MT305417^#^ | MT305542^#^ | Masiulionis et al. (2015),  Osti and Rodrigues (2018) |
| *Escovopsis weberi* | *Escovopsis weberi* | ATCC 64542 ^ET^ |  | Viçosa, Minas Gerais, Brazil |  | Carpenter ant fungal mass | KF293285 | KF293281 | MZ170961^#^ | MT305412^#^ | MT305537^#^ | Augustin et al. (2013) and this study |
| *Escovopsis* sp. | *Escovopsis weberi* | LESF 046 | SES001 | Rio Claro, São Paulo, Brazil | 22°23'45.9''S; 47°32'43.2''W | Fungus garden of *Trachymyrmex* sp. | KM817084 | MT273511^#^ | KM817146 | MT305436^#^ | MT305561^#^ | Meirelles et al. (2015b) |
| *Escovopsis* sp. | *Escovopsis weberi* | LESF 355 | ES021 | Corumbataí, São Paulo, Brazil |  | Fungus garden of *Atta sexdens rubropilosa* | MT273445 | MT273534 | MT305358 | MT305468 | MT305593 | This study |
| *Escovopsis* sp. | *Escovopsis weberi* | LESF 017 | NL001 | Botucatu, São Paulo, Brazil | 22°50'46.44''S; 48°26'9.6''W | Midden of *Atta capiguara* | KM817072 | MH715113 | KM817142 | MT305422^#^ | MT305547^#^ | Meirelles et al. (2015b) |
| *Escovopsis* sp. | *Escovopsis weberi* | LESF 019 | NL005 | Botucatu, São Paulo, Brazil | 22°50'45.8''S; 48°26'09.4''W | Fungus garden of *Atta sexdens rubropilosa* | KM817074 | MH715115 | KM817144 | MT305423^#^ | MT305548^#^ | Meirelles et al. (2015b) |
| *Escovopsis* sp. | *Escovopsis weberi* | LESF 020 | NL006 | Botucatu, São Paulo, Brazil | 22°50'45.8''S; 48°26'09.4''W | Fungus garden of *Atta sexdens rubropilosa* | MT273425 | MT273503 | MT305340 | MT305424 | MT305549 | This study |
| *Escovopsis* sp. | *Escovopsis weberi* | LESF 023 | ES005 | Alta Floresta, Mato Grosso, Brazil |  | Fungus garden of *Atta cephalotes* | KM817056 | MH715117 | KM817126 | MT305425^#^ | MT305550^#^ | Meirelles et al. (2015b),  Montoya et al. (2019) |
| *Escovopsis* sp. | *Escovopsis weberi* | LESF 024 | ES006 | Alta Floresta, Mato Grosso, Brazil |  | Fungus garden of *Acromyrmex coronatus* | KM817057 | MT273504^#^ | KM817127 | MT305426^#^ | MT305551^#^ | Montoya et al. (2019) |
| *Escovopsis* sp. | *Escovopsis weberi* | LESF 025 | ES007 | Alta Floresta, Mato Grosso, Brazil |  | Fungus garden of *Acromyrmex coronatus* | KM817058 | MT273505^#^ | KM817128 | MT305427^#^ | MT305552^#^ | Montoya et al. (2019) |
| *Escovopsis* sp. | *Escovopsis weberi* | LESF 027 | ES010 | Rio Claro, São Paulo, Brazil |  | Fungus garden of *Acromyrmex landolti* | KM817061 | MH715119 | KM817131 | MT305428^#^ | MT305553^#^ | Meirelles et al. (2015b),  Montoya et al. (2019) |
| *Escovopsis* sp. | *Escovopsis weberi* | LESF 029 | ES012 | Corumbataí, São Paulo, Brazil | 22°17'22''S;  47°39'23''W | Fungus garden of *Atta sexdens* | KM817063 | MH715120 | KM817133 | MT305429^#^ | MT305554^#^ | Meirelles et al. (2015b),  Montoya et al. (2019) |
| *Escovopsis* sp. | *Escovopsis weberi* | LESF 030 | ES013 | Corumbataí, São Paulo, Brazil | 22°17'22''S;  47°39'23''W | Fungus garden of *Atta sexdens* | KM817064 | MH715121 | KM817134 | MT305430^#^ | MT305555^#^ | Meirelles et al. (2015b),  Montoya et al. (2019) |
| *Escovopsis* sp. | *Escovopsis weberi* | LESF 031 | ES014 | Corumbataí, São Paulo, Brazil | 22°17'22''S;  47°39'23''W | Fungus garden of *Atta sexdens* | MT273426 | MT273506 | MT305341 | MT305431 | MT305556 | This study |
| *Escovopsis* sp. | *Escovopsis weberi* | LESF 033 | ES004 | Bahia, Brazil |  | Fungus garden of *Acromyrmex* sp. | KM817055 | MT273507^#^ | KM817125 | MT305432^#^ | MT305557^#^ | Meirelles et al. (2015b) |
| *Escovopsis* sp. | *Escovopsis weberi* | LESF 034 | ES024 | Botucatu, São Paulo, Brazil |  | Fungus garden of *Acromyrmex balzanii* | MT273427 | MT273508 | MT305342 | MT305433 | MT305558 | This study |
| *Escovopsis* sp. | *Escovopsis weberi* | LESF 042 | RS053 | Chuvisca, Rio Grande do Sul, Brazil | 30°50'10.2"S; 51°55'10.4"W | Fungus garden of *Acromyrmex lundii* | KM817079 | MT273509^#^ | EU082797 | MT305434^#^ | MT305559^#^ | Meirelles et al. (2015b) |
| *Escovopsis* sp. | *Escovopsis weberi* | LESF 043 | RS055 | Chuvisca, Rio Grande do Sul, Brazil | 30°50'10.2"S; 51°55'10.4"W | Fungus garden of *Acromyrmex heyeri* | KM817080 | MT273510^#^ | EU082796 | MT305435^#^ | MT305560^#^ | Meirelles et al. (2015b) |
| *Escovopsis* sp. | *Escovopsis weberi* | LESF 054 | AR003 | Ilhéus, Bahia, Brazil | 14°47'56.8''S; 39°10'16.4''W | Fungus garden of *Acromyrmex* *balzanii* | KM817043 | MT273512^#^ | KM817113 | MT305438^#^ | MT305563^#^ | Meirelles et al. (2015b) |
| *Escovopsis* sp. | *Escovopsis weberi* | LESF 056 | AR033 | Camacan, Bahia, Brazil | 15°22'50.3''S; 39°34'03.5''W | Fungus garden of *Acromyrmex* sp. | KM817045 | MT273513^#^ | KM817115 | MT305439^#^ | MT305564^#^ | Meirelles et al. (2015b) |
| *Escovopsis* sp. | *Escovopsis weberi* | LESF 136 | 4a | Corumbataí, São Paulo, Brazil | 22°17'21.7''S; 47°39'22.8''W | Fungus garden of *Atta sexdens rubropilosa* | MT273428 | MT273514 | MT305343 | MT305440 | MT305565 | This study |
| *Escovopsis* sp. | *Escovopsis weberi* | LESF 146 | 1cT4 | Corumbataí, São Paulo, Brazil | 22°17'21.7''S; 47°39'22.8''W | Fungus garden of *Atta sexdens rubropilosa* | MT273429 | MT273515 | MT305344 | MT305441 | MT305566 | This study |
| *Escovopsis* sp. | *Escovopsis weberi* | LESF 156 | A088 | Corumbataí, São Paulo, Brazil | 22°17'21.7''S; 47°39'22.8''W | Fungus garden of *Atta sexdens rubropilosa* | MT273430 | MT273516 | MT305345 | MT305443 | MT305568 | This study |
| *Escovopsis* sp. | *Escovopsis weberi* | LESF 178 | A086a | Corumbataí, São Paulo, Brazil | 22°17'21.7''S; 47°39'22.8''W | Fungus garden of *Atta sexdens rubropilosa* | MT273431 | MT273517 | MT305346 | MT305445 | MT305570 | This study |
| *Escovopsis* sp. | *Escovopsis weberi* | LESF 239 | 13B | Corumbataí, São Paulo, Brazil | 22°17'21.7''S; 47°39'22.8''W | Fungus garden of *Atta sexdens rubropilosa* | MT273432 | MT273518 | MT305347 | MT305446 | MT305571 | This study |
| *Escovopsis* sp. | *Escovopsis weberi* | LESF 241 | H1b | Corumbataí, São Paulo, Brazil | 22°17'21.7''S; 47°39'22.8''W | Fungus garden of *Atta sexdens rubropilosa* | MT273433 | MT273519 | MT305348 | MT305447 | MT305572 | This study |
| *Escovopsis* sp. | *Escovopsis weberi* | LESF 292 | NL003 | Botucatu, São Paulo, Brazil | 22°50'46.4"S 48°26'09.6"W | Fungus garden of *Atta capiguara* | MT273434 | MT273520 | MT305349 | MT305448 | MT305573 | This study |
| *Escovopsis* sp. | *Escovopsis weberi* | LESF 294 | H33 | Corumbataí, São Paulo, Brazil | 22°17'21.7''S; 47°39'22.8''W | Fungus garden of *Atta sexdens rubropilosa* | MT273435 | MT273521 | MT305350 | MT305449 | MT305574 | This study |
| *Escovopsis* sp. | *Escovopsis weberi* | LESF 295 | NL009 | Botucatu, São Paulo, Brazil | 22°50'45.8''S; 48°26'09.4''W | Fungus garden of *Atta sexdens rubropilosa* | MT273436 | MT273522 | MT305351 | MT305450 | MT305575 | This study |
| *Escovopsis* sp. | *Escovopsis weberi* | LESF 298 | NL004 | Botucatu, São Paulo, Brazil | 22°50'46.4"S 48°26'09.6"W | Fungus garden of *Atta capiguara* | MT273437 | MT273523 | MT305352 | MT305451 | MT305576 | This study |
| *Escovopsis* sp. | *Escovopsis weberi* | LESF 315 | NL007 | Botucatu, São Paulo, Brazil | 22°50'45.8''S; 48°26'09.4''W | Fungus garden of *Atta sexdens rubropilosa* | KM817075 | MH715125 | KF240730 | MT305463^#^ | MT305588^#^ | Meirelles et al. (2015b),  Montoya et al. (2019) |
| *Escovopsis* sp. | *Escovopsis weberi* | LESF 317 | ES026 | Rio Claro, São Paulo, Brazil |  | Fungus garden of *Trachymyrmex* sp. | KM817067 | MT273531^#^ | KM817137 | MT305464^#^ | MT305589^#^ | Meirelles et al. (2015b) |
| *Escovopsis* sp. | *Escovopsis weberi* | LESF 319 | ES030 | Palmas, Tocantins, Brazil | 10°10'52.9"S; 48°21'42.0"W | Fungus garden of *Acromyrmex* sp. | KM817070 | MT273532 | KM817140 | MT305465^#^ | MT305590^#^ | Meirelles et al. (2015b) |
| *Escovopsis* sp. | *Escovopsis weberi* | LESF 324 | RS105 | Thermas de Santa Bárbara, São Paulo, Brazil | 22º49'10.6"S; 49º16'06.2"W | Fungus garden of *Atta laevigata* | KM817083 | MT273533^#^ | KM817145 | MT305466^#^ | MT305591^#^ | Meirelles et al. (2015b) |
| *Escovopsis* sp. | *Escovopsis weberi* | LESF 356 | ES032 | Botucatu, São Paulo, Brazil |  | Fungus garden of *Atta laevigata* | MT273446 | MT273535 | MT305359 | MT305469 | MT305594 | This study |
| *Escovopsis* sp. | *Escovopsis weberi* | LESF 359 | ES019 | Corumbataí, São Paulo, Brazil |  | Fungus garden of *Atta sexdens* | MT273447 | MT273536 | MT305360 | MT305470 | MT305595 | This study |
| *Escovopsis* sp. | *Escovopsis weberi* | LESF 362 | ES028 | Corumbataí, São Paulo, Brazil |  | Fungus garden of *Atta sexdens* | MT273448 | MT273537 | MT305361 | MT305471 | MT305596 | This study |
| *Escovopsis* sp. | *Escovopsis weberi* | LESF 363 | ES023 | Corumbataí, São Paulo, Brazil |  | Fungus garden of *Atta sexdens* | MT273449 | MT273538 | MT305362 | MT305472 | MT305597 | This study |
| *Escovopsis* sp. | *Escovopsis weberi* | LESF 364 | ES015 | Corumbataí, São Paulo, Brazil |  | Fungus garden of *Atta sexdens* | MT273450 | MT273539 | MT305363 | MT305473 | MT305598 | This study |
| *Escovopsis* sp. | *Escovopsis weberi* | LESF 519 | ES016 |  |  | Fungus garden of *Atta sexdens rubropilosa* | MT273451 | MT273540 | MT305364 | MT305475 | MT305600 | This study |
| *Escovopsis* sp. | *Escovopsis weberi* | LESF 575 | RS087 | Indaial, Santa Catarina, Brazil | 26º54'04.9"S; 49º10'51.2"W | Fungus garden of *Acromyrmex diciger* | MT273452 | MT273541 | MT305365 | MT305476 | MT305601 | This study |
| *Escovopsis* sp. | *Escovopsis weberi* | LESF 858 | A210201 | Camacan, Bahia, Brazil |  | Fungus garden of *Atta cephalotes* | MT273461 | MT273550 | MT305373 | MT305497 | MT305622 | This study |
| *Escovopsis* sp. | *Escovopsis weberi* | LESF 859 | B110302 | Camacan, Bahia, Brazil |  | Fungus garden of *Atta cephalotes* | MT273462 | MT273551 | MT305374 | MT305498 | MT305623 | This study |
| *Escovopsis* sp. | *Escovopsis weberi* | LESF 877 | NL010 |  |  |  | MT273466 | MT273555 | MT305376 | MT305500 | MT305625 | This study |
| *Escovopsis* sp. | *Escovopsis weberi* | LESF 880 | 2aT=3 |  |  |  | MT273467 | MT273556 | MT305377 | MT305501 | MT305626 | This study |
| *Escovopsis* sp. | *Escovopsis weberi* | LESF 994 | QVM81 | Novo Airão, Amazonas, Brazil | 2°36'37.9''S; 60°52'34.4''W | Fungus garden of *Acromyrmex* sp. | MT273479 | MT273568 | MT305388 | MT305512 | MT305637 | This study |
| *Escovopsis* sp*.* | *Escovopsis* sp*.* | LESF 052 | SES010 | Manaus, Amazonas, Brazil |  | Fungus garden of *Trachymyrmex diversus* | KM817093 | MH715124 | KM817154 | MT305437^#^ | MT305562^#^ | Meirelles et al. (2015b),  Montoya et al. (2019) |
| *Escovopsis* sp*.* | *Escovopsis* sp*.* | LESF 325 | BA004 | Camacan, Bahia, Brazil | 14°47'56.8''S; 39°10'16.4''W | Fungus garden of *Atta cephalotes* | KM817049 | MH715127 | KM817119 | MT305467^#^ | MT305592^#^ | Meirelles et al. (2015b),  Montoya et al. (2019) |
| *Escovopsis* sp. | *Escovopsis* sp. | LESF 962 | QVM49 | Novo Airão, Amazonas, Brazil | 2°16'15.7''S; 61°01'8.5''W | Fungus garden of *Acromyrmex* sp. | MT273475 | MT273564 | MT305384 | MT305508 | MT305633 | This study |
| *Escovopsis* sp*.* | *Escovopsis* sp*.* | LESF 969 | QVM56 | Novo Airão, Amazonas, Brazil | 2°31'23.4''S; 60°49'31.9''W | Fungus garden of *Apterostigma* sp. | MT273476 | MT273565 | MT305385 | MT305509 | MT305634 | This study |
| *Escovopsis* sp. | *Escovopsis* sp. | LESF 975 | QVM62 | Novo Airão, Amazonas, Brazil | 2°31'25.3''S; 60°49'33.1''W | Fungus garden of *Trachymyrmex* sp. | MT273477 | MT273566 | MT305386 | MT305510 | MT305635 | This study |
| *Escovopsis* sp. | *Escovopsis* sp. | LESF 979 | QVM66 | Novo Airão, Amazonas, Brazil |  | Fungus garden of *Trachymyrmex* sp | MT273478 | MT273567 | MT305387 | MT305511 | MT305636 | This study |
| *Escovopsis* sp. | *Escovopsis* sp. | LESF 996 | QVM83 | Novo Airão, Amazonas, Brazil | 2°32'02.7''S; 60°50'11.7''W | Fungus garden of *Apterostigma* sp. | MT273480 | MT273569 | MT305389 | MT305513 | MT305638 | This study |
| *Escovopsis* sp. | *Escovopsis* sp. | LESF 997 | QVM84 | Novo Airão, Amazonas, Brazil | 2°31'23.4''S; 60°49'31.9''W | Fungus garden of *Trachymyrmex* sp | MT273481 | MT273570 | MT305390 | MT305514 | MT305639 | This study |
| *Escovopsis* sp. | *Escovopsis* sp. | LESF 1003 | QVM90 | Novo Airão, Amazonas, Brazil | 2°32'1.4''S;  60°50'0.4''W | Fungus garden of *Trachymyrmex* sp. | MT273482 | MT273571 | MT305391 | MT305515 | MT305640 | This study |
| *Escovopsis* sp. | *Sympodiorosea* sp. | LESF 864 | SES030331-05 |  |  |  | MT273464 | MT273553 | MT305375 | MT305499 | MT305624 | This study |
| *Escovopsis* sp. | *Sympodiorosea* sp. | LESF 886 | UGM23 |  |  |  | MT273468 | MT273557 | MT305378 | MT305502 | MT305627 | This study |
| *Escovopsis* sp. | *Sympodiorosea* sp. | LESF 887 | UGM26(C) |  |  |  | MT273469 | MT273558 | MT305379 | MT305503 | MT305628 | This study |
| *Escovopsis* sp. | *Sympodiorosea* sp. | LESF 899 | Q03V | Botucatu, São Paulo, Brazil | 22°54'19.6''S; 48°14'33.7''W | Fungus garden of *Mycocepurus goeldii* | MT273473 | MT273562 | MT305382 | MT305506 | MT305631 | This study |
| *Escovopsis* sp. | *Sympodiorosea* sp. | LESF 1010 | QVM138 | Manaus, Amazonas, Brazil | 2°26'51.6''S; 59°45'53.4''W | Fungus garden of *Apterostigma* sp. | MT273484 | MT273573 | MT305393 | MT305517 | MT305642 | This study |
| *Cladobotryum asterophorum* | *Cladobotryum asterophorum* | CBS 676.77 |  | Japan |  |  | FN859395 | AJ583469 | FN868712 | FN868776 | FN868649 | Põldmaa (2011) |
| *Cladobotryum protrusum* | *Cladobotryum protrusum* | TFC 201316 |  | Madagascar |  | Eucalyptus forest | FN859414 | FN859414 | FN868732 | FN868795 | FN868668 | Põldmaa (2011) |
| *Hypomyces samuelsii* | *Hypomyces samuelsii* | TFC 2007-23 |  | Peru |  | on  basidioma of an agaricoid basidiomycete on a stem of a palm | FN859451 | FN859451 | FN868769 | FN868828 | FN868705 | Põldmaa (2011) |
| *Hypomyces semicirculare* | *Hypomyces semicirculare* | CBS 705. 88 |  | Cuba |  | On old  polypore | NR_121425 | FN859417 | FN868735 | FN868671 | FN868798 | Põldmaa (2011) |
| *Lecanicillium antillanum* | *Lecanicillium antillanum* | CBS 350.85 |  | Cuba |  | on  basidioma of an agaricoid | NR_111097 | AF339536 | DQ522350 | DQ522396 | DQ522450 | Spatafora et al. (2007) |
| *Protocrea*  *pallida* | *Protocrea pallida* | TFC 99-209 |  | New York, Cleaveland |  |  | NR_111329 | EU710769 | EU703903 | --- | EU703949 | Jaklitsch et al. (2011) |
| *Sphaerostilbella aureonitens* | *Sphaerostilbella aureonitens* | GJS 74-87 |  |  |  |  | FJ442633 | HM466683 | FJ467644 | --- | FJ442763 | unpublished |
| *Trichoderma harzianum* | *Trichoderma harzianum* | CBS 226.95 |  | England |  |  | AY605713 | HM466680 | AF534621 | JQ031082 | AF545549 | Chaverri et al. (2003) |

^ET^ Ex-type cultures; ^#^ sequences obtained in this study. LESF: Laboratory of Fungal Ecology and Systematics (UNESP, Rio Claro, Brazil). QVM: Quimi Vidaurre Montoya.

Table S2. Molecular markers, primers and polymerase chain reaction (PCR) conditions used in this study.

| **Marker** | **Primers** | **PCR conditions** | **References** |
| --- | --- | --- | --- |
| ITS | ITS4 (5’TCCTCCGCTTATTGATATGC3’)  ITS5 (5’GGAAGTAAAAGTCGTAACAAGG3’) | 96°C for 3 min, 35 cycles at 94°C for 1 min, 55°C for 1 min and a final  extension step at 72°C for 2 min | White et al. (1990);  Schoch et al. (2012) |
| *tef*1 | EF6–20F (5’AAGAACATGATCACTGGTACCT3’)  EF6–1000R (5’CGCATGTCRCGGACGGC3’) | 96°C for 3 min, 35 cycles at 96°C for 30 s, 61°C for 45 s and a final  extension step at 72°C for 1 min | Taerum et al. (2007) |
| LSU | CLA-F (5’GCATATCAATAAGCGGAGGA3’)  CLA-R (5’GACTCCTTGGTCCGTGTTTCA3’) | 96°C for 3 min, 35 cycles at 94°C for 1 min, 55°C for 1 min and a final  extension step at 72°C for 2 min | White et al. (1990), Haugland and Heckman (1998), Currie et al. (2003) |
| *rpb1* | RPB1-Af, RPB1Ac (5’GARTGYCCDGGDCAYTTYGG3’)  RPB1-Cr (5’CCNGCDATNTCRTTRTCCATRTA3’) | 96 °C for 5 min followed by 15 cycles at 94 °C for 30s, 65 °C for 1.5 min, (the annealing temperature gradually decreased 1 °C by cycle), and 72 °C for 1.5 min; and 35 cycles at 94°C for 30s, 50 °C for 1min and 72 °C for 1 min. | Liu et al. (1999) (primers),  This study (conditions) |
| *rpb2* | fRPB2-5F (F) (5’GA(T/C)GA(T/C)(A/C)G(A/T)GATCA(T/C)TT(T/C)GG-3’)  fRPB2-7cR (R) (5’CCCAT(A/G)GCTTG(T/C)TT(A/G)CCCAT3’) | 96 °C for 5 min followed by 15 cycles at 94 °C for 30s, 65 °C for 1 min, (the annealing temperature gradually decreased 1 °C by cycle), and 72 °C for 1 min; and 35 cycles at 94°C for 30s, 50 °C for 1min and 72 °C for 1 min. | Liu et al. (1999) (primers),  This study (conditions) |

Table S3. Sequences and their associated metadata used to show the phylogenetic placement of all strains previously named as *Escovopsis* (Figs. 1 and S2). This table contains a total of 440 *tef*1 sequences from 274 strains from vesiculate-*Escovopsis* spp., 105 strains from non-vesiculate *Escovopsis* [24 strains from *Luteomyces trichodermoides* (previously introduced as “*Escovopsis trichodermoides*”), 57 strains from *Sympodiorosea* spp. (9 *Sympodiorosea kreiselii* previously introduced as “*Escovopsis kreiselii*”, 5 *Sympodiorosea* spp. introduced in this study, 6 *Sympodiorosea* spp. previously introduced as “*Escovopsis*”, 37 *Sympodiorosea* spp. previously introduced as “Pink *Escovopsis*”), 24 strains labeled as “?” (because they form two new clades that likely correspond to new genera – 20 previously introduced as “White *Escovopsis*”, 2 as “Yellow *Escovopsis*, and 2 as “*Escovopsis*”)], 60 strains from five *Hypocreaceae* genera [*Escovopsioides*, *Hypomyces* (along with species under its anamorphic genus *Cladobotryum*), *Protocrea*, *Sphaerostilbella*, and *Trichoderma*], and *Lecanicillium antillanum* CBS 350.85 as the outgroup.

| **Previous fungal species name** | **Current fungal species name** | **Strain ID** | **Specimen voucher** | **City, State, Country** | **Habitat** | **GenBank accessions *tef*1** | **References** |
| --- | --- | --- | --- | --- | --- | --- | --- |
| *Escovopsis* *aspergilloides* | *Escovopsis* *aspergilloides* | CBS 423.93 ^ET^ | DAOM:216382 | Trinidad and Tobago: Trinidad | Fungus garden of *Trachymyrmex ruthae* | AY172632 | Augustin et al. (2013),  Currie et al. (2003) |
| *Escovopsis clavata* | *Escovopsis clavata* | LESF 854 | 1704A | Florianópolis, Santa Catarina, Brazil | Fungus garden of *Apterostigma* sp. | MH724271 | Montoya et al. (2019) |
| *Escovopsis clavata* | *Escovopsis clavata* | LESF 855 | 1705B | Florianópolis, Santa Catarina, Brazil | Fungus garden of *Apterostigma* sp. | MH724272 | Montoya et al. (2019) |
| *Escovopsis clavata* | *Escovopsis clavata* | CBS 145326 ^ET^ | 1707 | Florianópolis, Santa Catarina, Brazil | Fungus garden of *Apterostigma* sp. | MH724270 | Montoya et al. (2019) |
| *Escovopsis lentecrescens* | *Escovopsis lentecrescens* | CBS 135750 ^ET^ | VIC:31755 | Viçosa, Minas Gerais, Brazil | Fungus garden of *Acromyrmex subterraneus subterraneus* | JQ855714 | Augustin et al. (2013) |
| *Escovopsis microspora* | *Escovopsis microspora* | CBS 135751^ET^ | VIC:31756 | Viçosa, Minas Gerais, Brazil | Fungus garden of *Acromyrmex subterraneus molestans* | KJ935030^&^ | Augustin et al. (2013),  Meirelles et al. (2015a) |
| *Escovopsis moelleri* | *Escovopsis moelleri* | CBS 135748 ^ET^ | VIC:31753 | Viçosa, Minas Gerais, Brazil | Fungus garden of *Acromyrmex subterraneus molestans* | JQ855712 | Augustin et al. (2013) |
| *Escovopsis multiformis* | *Escovopsis multiformis* | LESF 1136 | QVM277 | Alta Floresta, Mato Grosso, Brazil | Fungus garden of *Apterostigma* sp. | MH724266 | Montoya et al. (2019) |
| *Escovopsis multiformis* | *Escovopsis multiformis* | CBS 145327 ^ET^ | 1606w | Florianópolis, Santa Catarina, Brazil | Fungus garden of *Apterostigma* sp. | MH724265 | Montoya et al. (2019) |
| *Escovopsis* sp. | *Escovopsis multiformis* | LESF 852 | 1706B | Florianópolis, Santa Catarina, Brazil | Fungus garden of *Apterostigma* sp. | MT305372 | This study |
| *Escovopsis weberi* | *Escovopsis weberi* | ATCC 64542 ^ET^ |  | Viçosa, Minas Gerais, Brazil | Carpenter ant fungal mass | MZ170961^#^ | This study |
| *Escovopsis weberi* | *Escovopsis weberi* | AtcoN1C2I1 |  | Panama | Fungus garden of *Atta colombica* | GQ240710 | Taerum et al. (2010) |
| *Escovopsis weberi* | *Escovopsis weberi* | AtceN1S3Pa |  | Panama | Fungus garden of *Atta cephalotes* | GQ240704 | Taerum et al. (2010) |
| *Escovopsis weberi* | *Escovopsis weberi* | AtceN1S2Pa |  | Panama | Fungus garden of *Atta cephalotes* | GQ240703 | Taerum et al. (2010) |
| *Escovopsis weberi* | *Escovopsis weberi* | AtcoN1C2I2 |  | Panama | Fungus garden of *Atta colombica* | GQ240711 | Taerum et al. (2010) |
| *Escovopsis weberi* | *Escovopsis weberi* | AtcoN1C2I3 |  | Panama | Fungus garden of *Atta colombica* | GQ240712 | Taerum et al. (2010) |
| *Escovopsis* sp. | *Escovopsis weberi* | LESF 046 | SES001 | Rio Claro, São Paulo, Brazil | Fungus garden of *Trachymyrmex* sp. | KM817146 | Meirelles et al. (2015b) |
| *Escovopsis* sp. | *Escovopsis weberi* | LESF 355 | ES021 | Corumbataí, São Paulo, Brazil | Fungus garden of *Atta sexdens rubropilosa* | MT305358 | This study |
| *Escovopsis* sp. | *Escovopsis weberi* | LESF 017 | NL001 | Botucatu, São Paulo, Brazil | Midden of *Atta capiguara* | KM817142 | Meirelles et al. (2015b) |
| *Escovopsis* sp. | *Escovopsis weberi* | LESF 019 | NL005 | Botucatu, São Paulo, Brazil | Fungus garden of *Atta sexdens rubropilosa* | KM817144 | Meirelles et al. (2015b) |
| *Escovopsis* sp. | *Escovopsis weberi* | LESF 020 | NL006 | Botucatu, São Paulo, Brazil | Fungus garden of *Atta sexdens rubropilosa* | MT305340 | This study |
| *Escovopsis* sp. | *Escovopsis weberi* | LESF 023 | ES005 | Alta Floresta, Mato Grosso, Brazil | Fungus garden of *Atta cephalotes* | KM817126 | Meirelles et al. (2015b),  Montoya et al. (2019) |
| *Escovopsis* sp. | *Escovopsis weberi* | LESF 024 | ES006 | Alta Floresta, Mato Grosso, Brazil | Fungus garden of *Acromyrmex coronatus* | KM817127 | Montoya et al. (2019) |
| *Escovopsis* sp. | *Escovopsis weberi* | LESF 025 | ES007 | Alta Floresta, Mato Grosso, Brazil | Fungus garden of *Acromyrmex coronatus* | KM817128 | Montoya et al. (2019) |
| *Escovopsis* sp. | *Escovopsis weberi* | LESF 027 | ES010 | Rio Claro, São Paulo, Brazil | Fungus garden of *Acromyrmex landolti* | KM817131 | Meirelles et al. (2015b),  Montoya et al. (2019) |
| *Escovopsis* sp. | *Escovopsis weberi* | LESF 029 | ES012 | Corumbataí, São Paulo, Brazil | Fungus garden of *Atta sexdens* | KM817133 | Meirelles et al. (2015b),  Montoya et al. (2019) |
| *Escovopsis* sp. | *Escovopsis weberi* | LESF 030 | ES013 | Corumbataí, São Paulo, Brazil | Fungus garden of *Atta sexdens* | KM817134 | Meirelles et al. (2015b),  Montoya et al. (2019) |
| *Escovopsis* sp. | *Escovopsis weberi* | LESF 031 | ES014 | Corumbataí, São Paulo, Brazil | Fungus garden of *Atta sexdens* | MT305341 | This study |
| *Escovopsis* sp. | *Escovopsis weberi* | LESF 033 | ES004 | Bahia, Brazil | Fungus garden of *Acromyrmex* sp. | KM817125 | Meirelles et al. (2015b) |
| *Escovopsis* sp. | *Escovopsis weberi* | LESF 034 | ES024 | Botucatu, São Paulo, Brazil | Fungus garden of *Acromyrmex balzanii* | MT305342 | This study |
| *Escovopsis* sp. | *Escovopsis weberi* | LESF 042 | RS053 | Chuvisca, Rio Grande do Sul, Brazil | Fungus garden of *Acromyrmex lundii* | EU082797 | Meirelles et al. (2015b) |
| *Escovopsis* sp. | *Escovopsis weberi* | LESF 043 | RS055 | Chuvisca, Rio Grande do Sul, Brazil | Fungus garden of *Acromyrmex heyeri* | EU082796 | Meirelles et al. (2015b) |
| *Escovopsis* sp. | *Escovopsis weberi* | LESF 054 | AR003 | Ilhéus, Bahia, Brazil | Fungus garden of *Acromyrmex* *balzanii* | KM817113 | Meirelles et al. (2015b) |
| *Escovopsis* sp. | *Escovopsis weberi* | LESF 056 | AR033 | Camacan, Bahia, Brazil | Fungus garden of *Acromyrmex* sp. | KM817115 | Meirelles et al. (2015b) |
| *Escovopsis* sp. | *Escovopsis weberi* | LESF 136 | 4a | Corumbataí, São Paulo, Brazil | Fungus garden of *Atta sexdens rubropilosa* | MT305343 | This study |
| *Escovopsis* sp. | *Escovopsis weberi* | LESF 146 | 1cT4 | Corumbataí, São Paulo, Brazil | Fungus garden of *Atta sexdens rubropilosa* | MT305344 | This study |
| *Escovopsis* sp. | *Escovopsis weberi* | LESF 156 | A088 | Corumbataí, São Paulo, Brazil | Fungus garden of *Atta sexdens rubropilosa* | MT305345 | This study |
| *Escovopsis* sp. | *Escovopsis weberi* | LESF 178 | A086a | Corumbataí, São Paulo, Brazil | Fungus garden of *Atta sexdens rubropilosa* | MT305346 | This study |
| *Escovopsis* sp. | *Escovopsis weberi* | LESF 239 | 13B | Corumbataí, São Paulo, Brazil | Fungus garden of *Atta sexdens rubropilosa* | MT305347 | This study |
| *Escovopsis* sp. | *Escovopsis weberi* | LESF 241 | H1b | Corumbataí, São Paulo, Brazil | Fungus garden of *Atta sexdens rubropilosa* | MT305348 | This study |
| *Escovopsis* sp. | *Escovopsis weberi* | LESF 292 | NL003 | Botucatu, São Paulo, Brazil | Fungus garden of *Atta capiguara* | MT305349 | This study |
| *Escovopsis* sp. | *Escovopsis weberi* | LESF 294 | H33 | Corumbataí, São Paulo, Brazil | Fungus garden of *Atta sexdens rubropilosa* | MT305350 | This study |
| *Escovopsis* sp. | *Escovopsis weberi* | LESF 295 | NL009 | Botucatu, São Paulo, Brazil | Fungus garden of *Atta sexdens rubropilosa* | MT305351 | This study |
| *Escovopsis* sp. | *Escovopsis weberi* | LESF 298 | NL004 | Botucatu, São Paulo, Brazil | Fungus garden of *Atta capiguara* | MT305352 | This study |
| *Escovopsis* sp. | *Escovopsis weberi* | LESF 315 | NL007 | Botucatu, São Paulo, Brazil | Fungus garden of *Atta sexdens rubropilosa* | KF240730 | Meirelles et al. (2015b),  Montoya et al. (2019) |
| *Escovopsis* sp. | *Escovopsis weberi* | LESF 317 | ES026 | Rio Claro, São Paulo, Brazil | Fungus garden of *Trachymyrmex* sp. | KM817137 | Meirelles et al. (2015b) |
| *Escovopsis* sp. | *Escovopsis weberi* | LESF 319 | ES030 | Palmas, Tocantins, Brazil | Fungus garden of *Acromyrmex* sp. | KM817140 | Meirelles et al. (2015b) |
| *Escovopsis* sp. | *Escovopsis weberi* | LESF 324 | RS105 | Thermas de Santa Bárbara, São Paulo, Brazil | Fungus garden of *Atta laevigata* | KM817145 | Meirelles et al. (2015b) |
| *Escovopsis* sp. | *Escovopsis weberi* | LESF 356 | ES032 | Botucatu, São Paulo, Brazil | Fungus garden of *Atta laevigata* | MT305359 | This study |
| *Escovopsis* sp. | *Escovopsis weberi* | LESF 359 | ES019 | Corumbataí, São Paulo, Brazil | Fungus garden of *Atta sexdens* | MT305360 | This study |
| *Escovopsis* sp. | *Escovopsis weberi* | LESF 362 | ES028 | Corumbataí, São Paulo, Brazil | Fungus garden of *Atta sexdens* | MT305361 | This study |
| *Escovopsis* sp. | *Escovopsis weberi* | LESF 363 | ES023 | Corumbataí, São Paulo, Brazil | Fungus garden of *Atta sexdens* | MT305362 | This study |
| *Escovopsis* sp. | *Escovopsis weberi* | LESF 364 | ES015 | Corumbataí, São Paulo, Brazil | Fungus garden of *Atta sexdens* | MT305363 | This study |
| *Escovopsis* sp. | *Escovopsis weberi* | LESF 519 | ES016 |  | Fungus garden of *Atta sexdens rubropilosa* | MT305364 | This study |
| *Escovopsis* sp. | *Escovopsis weberi* | LESF 575 | RS087 | Indaial, Santa Catarina, Brazil | Fungus garden of *Acromyrmex diciger* | MT305365 | This study |
| *Escovopsis* sp. | *Escovopsis weberi* | LESF 858 | A210201 | Camacan, Bahia, Brazil | Fungus garden of *Atta cephalotes* | MT305373 | This study |
| *Escovopsis* sp. | *Escovopsis weberi* | LESF 859 | B110302 | Camacan, Bahia, Brazil | Fungus garden of *Atta cephalotes* | MT305374 | This study |
| *Escovopsis* sp. | *Escovopsis weberi* | LESF 877 | NL010 |  |  | MT305376 | This study |
| *Escovopsis* sp. | *Escovopsis weberi* | LESF 880 | 2aT=3 |  |  | MT305377 | This study |
| *Escovopsis* sp. | *Escovopsis weberi* | LESF 994 | QVM81 | Novo Airão, Amazonas, Brazil | Fungus garden of *Acromyrmex* sp. | MT305388 | This study |
| *Escovopsis* sp. | *Escovopsis weberi* | Esc2 |  | Ecuador | Fungus garden of *Atta* cf. *cephalotes* | AY172620 | Currie et al. (2003) |
| *Escovopsis* sp. | *Escovopsis weberi* | LESF 106 | SES006 | Uberlândia, minas Gerais, Brazil | Fungus garden of *Trachymyrmex dichrou* | KM817150 | Meirelles et al. (2015b) |
| *Escovopsis* cf. *weberi* CRC-2007a | *Escovopsis weberi* | AcspAArM | CC030402-02 Acro Esc A | Argentina | Fungus garden of *Acromyrmex* sp. | EF589922 | Taerum et al. (2007) |
| *Escovopsis* cf. *weberi* CRC-2007a | *Escovopsis weberi* | AtspBr |  | Brazil | Fungus garden of *Atta* sp. | EF589916 | Taerum et al. (2007) |
| *Escovopsis* cf. *weberi* CRC-2007a | *Escovopsis weberi* | AtspAr2 | UGM030330-07 Atta Esc B | Argentina | Fungus garden of *Atta sexdens* | EF589910 | Taerum et al. (2007) |
| *Escovopsis* cf. *weberi* CRC-2007a | *Escovopsis weberi* | AtcoPaP | SP020523-01 A col Esc B slow | Panama | Fungus garden of *Atta colombica* | EF589912 | Taerum et al. (2007) |
| *Escovopsis* cf. *weberi* Ae291 | *Escovopsis* sp. | Ae291 |  |  | Fungus garden of *Acromyrmex echinatior* | EU283878 | Unpublished |
| *Escovopsis* cf. *weberi* CC031210-221 | *Escovopsis* sp. | CC031210-22 |  |  | Nets of *Acromyrmex octospinosus* CC031210-22 | EU283880 | Unpublished |
| *Escovopsis* cf. *weberi* CRC-2007a | *Escovopsis* sp. | AtsePaC1 | CC020529-01 | Panama | Fungus garden of *Atta sexdens* | EF589919 | Taerum et al. (2007) |
| *Escovopsis* cf. *weberi* CRC-2007a | *Escovopsis* sp. | AcecPaP1 | CC020610-02 Acro Esc 5 | Panama | Fungus garden of *Acromyrmex echinatior* | EF589920 | Taerum et al. (2007) |
| *Escovopsis* cf. *weberi* ST040116-01 | *Escovopsis* sp. | ST040116-1 |  |  | Nets of *Acromyrmex octospinosus* ST040116-01 | EU283870 | Unpublished |
| *Escovopsis* cf. *weberi* CRC-2007a | *Escovopsis* sp. | AchiArC | UGM030406-02 Acro hisp Esc B | Argentina | Fungus garden of *Acromyrmex hispidus* | EF589913 | Taerum et al. (2007) |
| *Escovopsis* cf. *weberi* CRC-2007a | *Escovopsis* sp. | AcocPaD | CC030106-15 Acro Darien Esc G fast | Panama | Fungus garden of *Acromyrmex octospinosus* | EF589942 | Taerum et al. (2007) |
| *Escovopsis* cf. *weberi* CRC-2007a | *Escovopsis* sp. | AtvoArC | SES030404-03 Atta col Esc E | Argentina | Fungus garden of Atta vollenweirderi | EF589921 | Taerum et al. (2007) |
| *Escovopsis* cf. *weberi* CRC-2007a | *Escovopsis* sp. | AcocPaP1 | Esc 4 #78 | Panama | Fungus garden of *Acromyrmex octospinosus* | EF589918 | Taerum et al. (2007) |
| *Escovopsis* cf. *weberi* CRC-2007a | *Escovopsis* sp. | AccoEcO | AGH030518-14 Acro Esc | Ecuador | Fungus garden of *Acromyrmex coronatus* | EF589915 | Taerum et al. (2007) |
| *Escovopsis* cf. *weberi* CRC-2007a | *Escovopsis* sp. | AtcePaP1 | CC031208-10 A ceph | Panama | Fungus garden of *Atta cephalotes* | EF589917 | Taerum et al. (2007) |
| *Escovopsis* cf. *weberi* CRC-2007a | *Escovopsis* sp. | AcspEc1 | AGH030518-14 Acro Esc | Ecuador | Fungus garden of *Acromyrmex coronatus* | EF589948 | Taerum et al. (2007) |
| *Escovopsis* cf. *weberi* CRC-2007a | *Escovopsis* sp. | AtcePaBdT | ST041021-01 CI | Panama | Fungus garden of *Atta cephalotes* | EF589914 | Taerum et al. (2007) |
| *Escovopsis* cf. *weberi* CRC-2007a | *Escovopsis* sp. | AcecPa1 | CC020610-02 Acro Esc 5 | Panama | Fungus garden of *Acromyrmex echinatior* | EF589949 | Taerum et al. (2007) |
| *Escovopsis* cf. *weberi* CRC-2007a | *Escovopsis* sp. | AcocPaP2 | CC020602-02 Acro Esc NMG | Panama | Fungus garden of *Acromyrmex octospinosus* | EF589937 | Taerum et al. (2007) |
| *Escovopsis* cf. *weberi* CRC-2007a | *Escovopsis* sp. | AtcoPaC2 | CC020529 A col fast | Panama | Fungus garden of *Atta colombica* | EF589936 | Taerum et al. (2007) |
| *Escovopsis* cf. *weberi* CRC-2007a | *Escovopsis* sp. | AcocPaC | ST041019-03 I | Panama | Fungus garden of *Acromyrmex octospinosus* | EF589933 | Taerum et al. (2007) |
| *Escovopsis* cf. *weberi* CRC-2007a | *Escovopsis* sp. | AcocGuBT | ASM031226-05 Acro octospinosus | Panama | Fungus garden of *Acromyrmex octospinosus* | EF589935 | Taerum et al. (2007) |
| *Escovopsis* cf. *weberi* Ae292 | *Escovopsis* sp. | Ae292 |  |  | Fungus garden of *Acromyrmex echinatior* | EU283879 | Unpublished |
| *Escovopsis* cf. *weberi* CRC-2007a | *Escovopsis* sp. | AtsePaC3 | ST041014-01 | Panama | Fungus garden of *Atta sexdens* | EF589939 | Taerum et al. (2007) |
| *Escovopsis* cf. *weberi* CRC-2007a | *Escovopsis* sp. | AcecPaC | ST041019-01 III | Panama | Fungus garden of *Acromyrmex echinatior* | EF589932 | Taerum et al. (2007) |
| *Escovopsis* cf. *weberi* CRC-2007a | *Escovopsis* sp. | AcecPaP2 | CC020610-02 Acro Esc 2 | Panama | Fungus garden of *Acromyrmex echinatior* | EF589943 | Taerum et al. (2007) |
| *Escovopsis* cf. *weberi* CC031212-1 | *Escovopsis* sp. | CC031212-1 |  |  | Fungus garden of *Acromyrmex echinatior* | EU283875 | Unpublished |
| *Escovopsis* cf. *weberi* UGM020518-5 | *Escovopsis* sp. | UGM020518-5 |  |  | Fungus garden of *Acromyrmex octospinosus* | EU283876 | Unpublished |
| *Escovopsis* cf. *weberi CC011010-4* | *Escovopsis* sp. | CC011010-4 |  |  | Fungus garden of *Acromyrmex octospinosus* CC011010-04 | EU283871 | Unpublished |
| *Escovopsis* cf. *weberi* CRC-2007a | *Escovopsis* sp. | AtmeMxV | SES030113-01 Esc 1 Atta Mexico | Mexico | Fungus garden of *Atta mexicana* | EF589938 | Taerum et al. (2007) |
| *Escovopsis* cf. *weberi* Ae295 | *Escovopsis* sp. | Ae295 |  |  | Fungus garden of *Acromyrmex echinatior* | EU283869 | Unpublished |
| *Escovopsis* cf. *weberi* CRC-2007a | *Escovopsis* sp. | AtsePaC2 | ST041013-01 I | Panama | Fungus garden of Atta sexdens | EF589931 | Taerum et al. (2007) |
| *Escovopsis* cf. *weberi UGM030330-4* | *Escovopsis* sp. | UGM030330-4 |  |  | Fungus garden of *Acromyrmex laticeps* | EU283873 | Unpublished |
| *Escovopsis* cf. *weberi* UGM030327-2 | *Escovopsis* sp. | UGM030327-2 |  |  | Fungus garden of *Acromyrmex hispidus fallax* UGM030327-02 | EU283877 | Unpublished |
| *Escovopsis* cf. *weberi* CRC-2007a | *Escovopsis* sp. | AcheArM | SP030401-01 Acro Esc B | Argentina | Fungus garden of *Acromyrmex heyeri* | EF589940 | Taerum et al. (2007) |
| *Escovopsis* cf. *weberi* CC030327-2 | *Escovopsis* sp. | CC030327-2 |  |  | *Acromyrmex niger*  CC030327-02 | EU283872 | Unpublished |
| *Escovopsis* cf. *weberi* CRC-2007a | *Escovopsis* sp. | AclaArM | UGM030330-05 Acro Esc B | Argentina | Fungus garden of *Acromyrmex laticeps* | EF589934 | Taerum et al. (2007) |
| *Escovopsis* cf. *weberi* CRC-2007a | *Escovopsis* sp. | AtseArM2 | CC030328-05 Atta Esc A | Argentina | Fungus garden of *Atta sexdens* | EF589941 | Taerum et al. (2007) |
| *Escovopsis* cf. *weberi* SP030327-1 | *Escovopsis* sp. | SP030327-1 |  |  | Fungus garden of *Acromyrmex hispidus fallax* SP030327-01 | EU283874 | Unpublished |
| *Escovopsis* cf. *weberi* CRC-2007a | *Escovopsis* sp. | AtcePaP2 | AL031210-29 A ceph | Panama | Fungus garden of *Atta cephalotes* | EF589923 | Taerum et al. (2007) |
| *Escovopsis* cf. *weberi* CRC-2007a | *Escovopsis* sp. | AtcePaP3 | AL031210-29 A ceph | Panama | Fungus garden of *Atta cephalotes* | EF589929 | Taerum et al. (2007) |
| *Escovopsis* cf. *weberi* CRC-2007a | *Escovopsis* sp. | AtcoEcO | NMG030611-01 Atta Esc | Ecuador | Fungus garden of *Atta colombica* | EF589926 | Taerum et al. (2007) |
| *Escovopsis* cf. *weberi* CRC-2007a | *Escovopsis* sp. | AtceEcO |  | Ecuador | Fungus garden of *Atta cephalotes* | EF589925 | Taerum et al. (2007) |
| *Escovopsis* cf. *weberi* CRC-2007a | *Escovopsis* sp. | AchyEcO2 | NMG030616-01 Atta Esc | Ecuador | Fungus garden of *Acromyrmex hystrix* | EF589927 | Taerum et al. (2007) |
| *Escovopsis* cf. *weberi* CRC-2007a | *Escovopsis* sp. | AchyEcO3 | CC030615-12 Acro Esc | Ecuador | Fungus garden of *Acromyrmex hystrix* | EF589928 | Taerum et al. (2007) |
| *Escovopsis* cf. *weberi* CRC-2007a | *Escovopsis* sp. | AchyEcO1 | AL030615-05 Acro Esc | Ecuador | Fungus garden of *Acromyrmex hystrix* | EF589924 | Taerum et al. (2007) |
| *Escovopsis* cf. *weberi* CRC-2007a | *Escovopsis* sp. | AchyEcO4 | AL030515-06 Acro Esc | Ecuador | Fungus garden of *Acromyrmex hystrix* | EF589930 | Taerum et al. (2007) |
| *Escovopsis* cf. weberi CRC-2007a | *Escovopsis* sp. | TrruTr | Esc 16 E. asperg. | Trinidad and Tobago | Fungus garden of *Trachymyrmex ruthae* | EF589946 | Taerum et al. (2007) |
| *Escovopsis* cf. weberi CRC-2007a | *Escovopsis* sp. | TrZePa | Esc 8 97T27 | Panama | Fungus garden of *Trachymyrmex zeteki* | EF589947 | Taerum et al. (2007) |
| *Escovopsis* cf. weberi CRC-2007a | *Escovopsis* sp. | TrcoEc | AGH030609-07 Trachy Esc | Ecuador | Fungus garden of *Trachymyrmex cornetzi* | EF589944 | Taerum et al. (2007) |
| *Escovopsis* cf. weberi CRC-2007a | *Escovopsis* sp. | TrdiEc | Esc 14 980218-06 | Ecuador | Fungus garden of *Trachymyrmex diversus* | EF589945 | Taerum et al. (2007) |
| *Escovopsis weberi* | *Escovopsis* sp. | AtcoN3C1I2 |  | Panama | Fungus garden of *Atta colombica* | GQ240733 | Taerum et al. (2010) |
| *Escovopsis weberi* | *Escovopsis* sp. | AtcoN4C3I5 |  | Panama | Fungus garden of *Atta colombica* | GQ240765 | Taerum et al. (2010) |
| *Escovopsis weberi* | *Escovopsis* sp. | AtcoN4C4I1 |  | Panama | Fungus garden of *Atta colombica* | GQ240767 | Taerum et al. (2010) |
| *Escovopsis weberi* | *Escovopsis* sp. | AtcoN3C1I4 |  | Panama | Fungus garden of *Atta colombica* | GQ240735 | Taerum et al. (2010) |
| *Escovopsis weberi* | *Escovopsis* sp. | AtcoN2C3I1 |  | Panama | Fungus garden of *Atta colombica* | GQ240728 | Taerum et al. (2010) |
| *Escovopsis weberi* | *Escovopsis* sp. | AtcoN4C4I2 |  | Panama | Fungus garden of *Atta colombica* | GQ240768 | Taerum et al. (2010) |
| *Escovopsis weberi* | *Escovopsis* sp. | AtcoN4C3I6 |  | Panama | Fungus garden of *Atta colombica* | GQ240766 | Taerum et al. (2010) |
| *Escovopsis weberi* | *Escovopsis* sp. | AtcoN4C3I1 |  | Panama | Fungus garden of *Atta colombica* | GQ240761 | Taerum et al. (2010) |
| *Escovopsis weberi* | *Escovopsis* sp. | AtcoN4C1I2 |  | Panama | Fungus garden of *Atta colombica* | GQ240753 | Taerum et al. (2010) |
| *Escovopsis weberi* | *Escovopsis* sp. | AtcoN2C3I2 |  | Panama | Fungus garden of *Atta colombica* | GQ240729 | Taerum et al. (2010) |
| *Escovopsis weberi* | *Escovopsis* sp. | AtcoN3C2I2 |  | Panama | Fungus garden of *Atta colombica* | GQ240739 | Taerum et al. (2010) |
| *Escovopsis weberi* | *Escovopsis* sp. | AtcoN3C1I3 |  | Panama | Fungus garden of *Atta colombica* | GQ240734 | Taerum et al. (2010) |
| *Escovopsis weberi* | *Escovopsis* sp. | AtcoN4C4I3 |  | Panama | Fungus garden of *Atta colombica* | GQ240769 | Taerum et al. (2010) |
| *Escovopsis weberi* | *Escovopsis* sp. | AtcoN2C2I3 |  | Panama | Fungus garden of *Atta colombica* | GQ240724 | Taerum et al. (2010) |
| *Escovopsis weberi* | *Escovopsis* sp. | AtcoN3C1I6 |  | Panama | Fungus garden of *Atta colombica* | GQ240737 | Taerum et al. (2010) |
| *Escovopsis weberi* | *Escovopsis* sp. | AtceN2S1Pa |  | Panama | Fungus garden of Atta cephalotes | GQ240705 | Taerum et al. (2010) |
| *Escovopsis weberi* | *Escovopsis* sp. | AtcoN2C2I5 |  | Panama | Fungus garden of *Atta colombica* | GQ240726 | Taerum et al. (2010) |
| *Escovopsis weberi* | *Escovopsis* sp. | AtcoN2C3I4 |  | Panama | Fungus garden of *Atta colombica* | GQ240731 | Taerum et al. (2010) |
| *Escovopsis weberi* | *Escovopsis* sp. | AtcoN3C4I3 |  | Panama | Fungus garden of *Atta colombica* | GQ240751 | Taerum et al. (2010) |
| *Escovopsis weberi* | *Escovopsis* sp. | AtcoN2C1I3 |  | Panama | Fungus garden of *Atta colombica* | GQ240715 | Taerum et al. (2010) |
| *Escovopsis weberi* | *Escovopsis* sp. | AtcoN4C2I4 |  | Panama | Fungus garden of *Atta colombica* | GQ240759 | Taerum et al. (2010) |
| *Escovopsis weberi* | *Escovopsis* sp. | AtcoN4C3I3 |  | Panama | Fungus garden of *Atta colombica* | GQ240763 | Taerum et al. (2010) |
| *Escovopsis weberi* | *Escovopsis* sp. | AtcoN3C2I4 |  | Panama | Fungus garden of *Atta colombica* | GQ240741 | Taerum et al. (2010) |
| *Escovopsis weberi* | *Escovopsis* sp. | AtcoN3C3I6 |  | Panama | Fungus garden of *Atta colombica* | GQ240748 | Taerum et al. (2010) |
| *Escovopsis weberi* | *Escovopsis* sp. | AtcoN4C1I4 |  | Panama | Fungus garden of *Atta colombica* | GQ240755 | Taerum et al. (2010) |
| *Escovopsis weberi* | *Escovopsis* sp. | AtcoN2C1I6 |  | Panama | Fungus garden of *Atta colombica* | GQ240718 | Taerum et al. (2010) |
| *Escovopsis weberi* | *Escovopsis* sp. | AtcoN3C3I2 |  | Panama | Fungus garden of *Atta colombica* | GQ240744 | Taerum et al. (2010) |
| *Escovopsis weberi* | *Escovopsis* sp. | AtcoN3C3I3 |  | Panama | Fungus garden of *Atta colombica* | GQ240745 | Taerum et al. (2010) |
| *Escovopsis weberi* | *Escovopsis* sp. | AtcoN2C1I5 |  | Panama | Fungus garden of *Atta colombica* | GQ240717 | Taerum et al. (2010) |
| *Escovopsis weberi* | *Escovopsis* sp. | AtcoN4C4I4 |  | Panama | Fungus garden of *Atta colombica* | GQ240770 | Taerum et al. (2010) |
| *Escovopsis weberi* | *Escovopsis* sp. | AtcoN4C2I3 |  | Panama | Fungus garden of *Atta colombica* | GQ240758 | Taerum et al. (2010) |
| *Escovopsis weberi* | *Escovopsis* sp. | AtcoN4C1I3 |  | Panama | Fungus garden of *Atta colombica* | GQ240754 | Taerum et al. (2010) |
| *Escovopsis weberi* | *Escovopsis* sp. | AtcoN3C2I3 |  | Panama | Fungus garden of *Atta colombica* | GQ240740 | Taerum et al. (2010) |
| *Escovopsis weberi* | *Escovopsis* sp. | AtcoN4C3I2 |  | Panama | Fungus garden of *Atta colombica* | GQ240762 | Taerum et al. (2010) |
| *Escovopsis weberi* | *Escovopsis* sp. | AtcoN2C1I2 |  | Panama | Fungus garden of *Atta colombica* | GQ240714 | Taerum et al. (2010) |
| *Escovopsis weberi* | *Escovopsis* sp. | AtcoN2C3I3 |  | Panama | Fungus garden of *Atta colombica* | GQ240730 | Taerum et al. (2010) |
| *Escovopsis weberi* | *Escovopsis* sp. | AtcoN1C1I1 |  | Panama | Fungus garden of *Atta colombica* | GQ240707 | Taerum et al. (2010) |
| *Escovopsis weberi* | *Escovopsis* sp. | AtcoN2C2I2 |  | Panama | Fungus garden of *Atta colombica* | GQ240723 | Taerum et al. (2010) |
| *Escovopsis weberi* | *Escovopsis* sp. | LESF 038 | RS004 | Brazil | Fungus garden of *Acromyrmex coronatus* | EU082798 | Meirelles et al. (2015b) |
| *Escovopsis weberi* | *Escovopsis* sp. | LESF 575 | RS087 | Brazil | Fungus garden of *Acromyrmex disciger* | EU082800 | Meirelles et al. (2015b) |
| *Escovopsis weberi* | *Escovopsis* sp. | AtceN1S1Pa |  | Panama | Fungus garden of *Atta cephalote* | GQ240702 | Taerum et al. (2010) |
| *Escovopsis weberi* | *Escovopsis* sp. | AtcoN2C1I1 |  | Panama | Fungus garden of *Atta colombica* | GQ240713 | Taerum et al. (2010) |
| *Escovopsis weberi* | *Escovopsis* sp. | AtcoN3C4I1 |  | Panama | Fungus garden of *Atta colombica* | GQ240749 | Taerum et al. (2010) |
| *Escovopsis weberi* | *Escovopsis* sp. | AcocN1S5Pa |  | Panama | Fungus garden of *Acromyrmex octospinosus* | GQ240697 | Taerum et al. (2010) |
| *Escovopsis weberi* | *Escovopsis* sp. | AtcoN3C3I4 |  | Panama | Fungus garden of *Atta colombica* | GQ240746 | Taerum et al. (2010) |
| *Escovopsis weberi* | *Escovopsis* sp. | AcocN1S4Pa |  | Panama | Fungus garden of *Acromyrmex octospinosus* | GQ240696 | Taerum et al. (2010) |
| *Escovopsis weberi* | *Escovopsis* sp. | AtcoN2C1I7 |  | Panama | Fungus garden of *Atta colombica* | GQ240719 | Taerum et al. (2010) |
| *Escovopsis weberi* | *Escovopsis* sp. | AcocN1S3Pa |  | Panama | Fungus garden of *Acromyrmex octospinosus* | GQ240695 | Taerum et al. (2010) |
| *Escovopsis weberi* | *Escovopsis* sp. | AtcoN2C2I1 |  | Panama | Fungus garden of *Atta colombica* | GQ240722 | Taerum et al. (2010) |
| *Escovopsis weberi* | *Escovopsis* sp. | AtcoN3C1I1 |  | Panama | Fungus garden of *Atta colombica* | GQ240732 | Taerum et al. (2010) |
| *Escovopsis weberi* | *Escovopsis* sp. | AtcoN4C2I5 |  | Panama | Fungus garden of *Atta colombica* | GQ240760 | Taerum et al. (2010) |
| *Escovopsis weberi* | *Escovopsis* sp. | AtcoN4C3I4 |  | Panama | Fungus garden of *Atta colombica* | GQ240764 | Taerum et al. (2010) |
| *Escovopsis weberi* | *Escovopsis* sp. | AtcoN4C1I1 |  | Panama | Fungus garden of *Atta colombica* | GQ240752 | Taerum et al. (2010) |
| *Escovopsis weberi* | *Escovopsis* sp. | AtcoN4C2I2 |  | Panama | Fungus garden of *Atta colombica* | GQ240757 | Taerum et al. (2010) |
| *Escovopsis weberi* | *Escovopsis* sp. | AcocN1S6Pa |  | Panama | Fungus garden of *Atta colombica* | GQ240698 | Taerum et al. (2010) |
| *Escovopsis weberi* | *Escovopsis* sp. | AtcoN3C3I5 |  | Panama | Fungus garden of *Atta colombica* | GQ240747 | Taerum et al. (2010) |
| *Escovopsis weberi* | *Escovopsis* sp. | AcocN1S1Pa |  | Panama | Fungus garden of *Atta octospinosus* | GQ240693 | Taerum et al. (2010) |
| *Escovopsis weberi* | *Escovopsis* sp. | AtcoN2C2I4 |  | Panama | Fungus garden of *Atta colombica* | GQ240725 | Taerum et al. (2010) |
| *Escovopsis weberi* | *Escovopsis* sp. | AtcoN3C4I2 |  | Panama | Fungus garden of *Atta colombica* | GQ240750 | Taerum et al. (2010) |
| *Escovopsis weberi* | *Escovopsis* sp. | AtcoN3C1I5 |  | Panama | Fungus garden of *Atta colombica* | GQ240736 | Taerum et al. (2010) |
| *Escovopsis weberi* | *Escovopsis* sp. | AtcoN2C1I8 |  | Panama | Fungus garden of *Atta colombica* | GQ240720 | Taerum et al. (2010) |
| *Escovopsis weberi* | *Escovopsis* sp. | AtcoN2C1I9 |  | Panama | Fungus garden of *Atta colombica* | GQ240721 | Taerum et al. (2010) |
| *Escovopsis weberi* | *Escovopsis* sp. | AtcoN3C2I5 |  | Panama | Fungus garden of *Atta colombica* | GQ240742 | Taerum et al. (2010) |
| *Escovopsis weberi* | *Escovopsis* sp. | AtcoN4C2I1 |  | Panama | Fungus garden of *Atta colombica* | GQ240756 | Taerum et al. (2010) |
| *Escovopsis weberi* | *Escovopsis* sp. | AtcoN1C1I3 |  | Panama | Fungus garden of *Atta colombica* | GQ240709 | Taerum et al. (2010) |
| *Escovopsis weberi* | *Escovopsis* sp. | AtcoN3C3I1 |  | Panama | Fungus garden of *Atta colombica* | GQ240743 | Taerum et al. (2010) |
| *Escovopsis weberi* | *Escovopsis* sp. | AtcoN2C1I4 |  | Panama | Fungus garden of *Atta colombica* | GQ240716 | Taerum et al. (2010) |
| *Escovopsis weberi* | *Escovopsis* sp. | AtcoN3C2I |  | Panama | Fungus garden of *Atta colombica* | GQ240738 | Taerum et al. (2010) |
| *Escovopsis weberi* | *Escovopsis* sp. | AcecN1S1Pa |  | Panama | Fungus garden of *Acromyrmex echinatior* | GQ240691 | Taerum et al. (2010) |
| *Escovopsis weberi* | *Escovopsis* sp. | AcspN1S1Ar |  | Argentina | Fungus garden of *Acromyrmex* sp. | GQ240699 | Taerum et al. (2010) |
| *Escovopsis weberi* | *Escovopsis* sp. | AcocN1S2Pa |  | Panama | Fungus garden of *Acromyrmex octospinosus* | GQ240694 | Taerum et al. (2010) |
| *Escovopsis weberi* | *Escovopsis* sp. | AtcoN2C2I6 |  | Panama | Fungus garden of *Atta colombica* | GQ240727 | Taerum et al. (2010) |
| *Escovopsis* sp. | *Escovopsis* sp*.* | LESF 052 | SES010 | Manaus, Amazonas, Brazil | Fungus garden of *Trachymyrmex diversus* | KM817154 | Meirelles et al. (2015b) |
| *Escovopsis* sp. | *Escovopsis* sp*.* | LESF 325 | BA004 | Camacan, Bahia, Brazil | Fungus garden of *Atta cephalotes* | KM817119 | Meirelles et al. (2015b) |
| *Escovopsis* sp. | *Escovopsis* sp. | LESF 962 | QVM49 | Novo Airão, Amazonas, Brazil | Fungus garden of *Acromyrmex* sp. | MT305384 | This study |
| *Escovopsis* sp. | *Escovopsis* sp*.* | LESF 969 | QVM56 | Novo Airão, Amazonas, Brazil | Fungus garden of *Apterostigma* sp. | MT305385 | This study |
| *Escovopsis* sp. | *Escovopsis* sp. | LESF 975 | QVM62 | Novo Airão, Amazonas, Brazil | Fungus garden of *Trachymyrmex* sp. | MT305386 | This study |
| *Escovopsis* sp. | *Escovopsis* sp. | LESF 979 | QVM66 | Novo Airão, Amazonas, Brazil | Fungus garden of *Trachymyrmex* sp | MT305387 | This study |
| *Escovopsis* sp. | *Escovopsis* sp. | LESF 996 | QVM83 | Novo Airão, Amazonas, Brazil | Fungus garden of *Apterostigma* sp. | MT305389 | This study |
| *Escovopsis* sp. | *Escovopsis* sp. | LESF 997 | QVM84 | Novo Airão, Amazonas, Brazil | Fungus garden of *Trachymyrmex* sp | MT305390 | This study |
| *Escovopsis* sp. | *Escovopsis* sp. | LESF 1003 | QVM90 | Novo Airão, Amazonas, Brazil | Fungus garden of *Trachymyrmex* sp. | MT305391 | This study |
| *Escovopsis* sp. | *Escovopsis* sp. | Acol2 |  | Panama |  | AY172622 | Currie et al. (2003) |
| *Escovopsis* sp. | *Escovopsis* sp. | Esc4 |  | Panama | Fungus garden of *Acromyrmex octospinosus* | AY172617 | Currie et al. (2003) |
| *Escovopsis* sp. | *Escovopsis* sp. | LESF 040 | RS020 | Nova Petrópolis, Rio Grande do Sul, Brazil | Fungus garden of *Acromyrmex laticeps* AOMB060904-05 | EU082803 | Meirelles et al. (2015b) |
| *Escovopsis* sp. | *Escovopsis* sp. | LESF 041 | RS030 | Brazil | Fungus garden of *Acromyrmex lundi* | EU082795 | Meirelles et al. (2015b) |
| *Escovopsis* sp. | *Escovopsis* sp. | LESF 045 | RS076 | Brazil | Fungus garden of *Acromyrmex coronatus* | EU082801 | Meirelles et al. (2015b) |
| *Escovopsis* sp. | *Escovopsis* sp. | LESF 039 | RS019 | Brazil | Fungus garden of *Acromyrmex ambiguus* | EU082802 | Meirelles et al. (2015b) |
| *Escovopsis* sp. | *Escovopsis* sp. | LESF 044 | RS061 | Brazil | Fungus garden of *Acromyrmex heyeri* | EU082799 | Meirelles et al. (2015b) |
| *Escovopsis* sp. | *Escovopsis* sp. | Esc90 |  | Panama | Fungus garden of *Acromyrmex echinatior* | AY172616 | Currie et al. (2003) |
| *Escovopsis* sp. Esc612 | *Escovopsis* sp. | Esc612 |  | Ecuador | Fungus garden of *Atta* cf. *cephalotes* | AY172621 | Taerum et al. (2007) |
| *Escovopsis* sp. Esc8 | *Escovopsis* sp. | Esc8 |  | Panama | Fungus garden of *Trachymyrmex* cf. *zeteki* | AY172630 | Currie et al. (2003) |
| *Escovopsis* sp. Esc14 | *Escovopsis* sp. | Esc14 |  | Ecuador | Fungus garden of *Trachymyrmex diversus* | AY172631 | Currie et al. (2003) |
| *Escovopsis* sp. | *Escovopsis* sp. | LESF 021 | ES002 | Rio Claro, São Paulo, Brazil | Fungus garden of *Atta sexdens* | KM817123 | Meirelles et al. (2015b) |
| *Escovopsis* sp. | *Escovopsis* sp. | LESF 028 | ES011 | Corumbataí, São Paulo, Brazil | Fungus garden of *Atta sexdens* | KM817132 | Meirelles et al. (2015b) |
| *Escovopsis* sp. | *Escovopsis* sp. | LESF 022 | ES003 | Frei Caneca, Pernambuco, Brazil | Fungus garden of *Atta cephalotes* | KM817124 | Meirelles et al. (2015b) |
| *Escovopsis* sp. | *Escovopsis* sp. | LESF 026 | ES009 | Carreiro da Várzea, Amazonas Brazil | Fungus garden of *Atta cephalotes* | KM817130 | Meirelles et al. (2015b) |
| *Escovopsis* sp. | *Escovopsis* sp. | LESF 037 | ES033 | Paraupebas, Pará, Brazil | Fungus garden of *Atta cephalotes* | KM817141 | Meirelles et al. (2015b) |
| *Escovopsis* sp. | *Escovopsis* sp. | LESF 843 | BA003 | Camacan, Bahia, Brazil | Fungus garden of *Atta cephalotes* | KM817118 | Meirelles et al. (2015b) |
| *Escovopsis* sp. | *Escovopsis* sp. | LESF 844 | BA005 | Camacan, Bahia, Brazil | Fungus garden of *Atta cephalotes* | KM817120 | Meirelles et al. (2015b) |
| *Escovopsis* sp. | *Escovopsis* sp. | LESF 326 | BA006 | Camacan, Bahia, Brazil | Fungus garden of *Atta cephalotes* | KM817121 | Meirelles et al. (2015b) |
| *Escovopsis* sp. | *Escovopsis* sp. | LESF 032 | ES008 | Santarém, Pará, Brazil | Fungus garden of *Acromyrmex sp.* | KM817129 | Meirelles et al. (2015b) |
| *Escovopsis* sp. | *Escovopsis* sp. | LESF 035 | ES025 | Botucatu, São Paulo, Brazil | Fungus garden of *Acromyrmex balzani* | KM817136 | Meirelles et al. (2015b) |
| *Escovopsis* sp. | *Escovopsis* sp. | LESF 036 | ES027 | Rio Claro, São Paulo, Brazil | Fungus garden of *Acromyrmex rugosus rugosus* | KM817138 | Meirelles et al. (2015b) |
| *Escovopsis* sp. | *Escovopsis* sp. | LESF 050 | SES008 | Rondônia, Brazil | Fungus garden of *Acromyrmex* sp. | KM817152 | Meirelles et al. (2015b) |
| *Escovopsis* sp. | *Escovopsis* sp. | LESF 055 | AR022 | Camacan, Bahia, Brazil | Fungus garden of *Acromyrmex* sp. | KM817114 | Meirelles et al. (2015b) |
| *Escovopsis* sp. | *Escovopsis* sp. | LESF 316 | ES001 | Rio Claro, São Paulo, Brazil | Fungus garden of *Trachymyrmex sp.* | KM817122 | Meirelles et al. (2015b) |
| *Escovopsis* sp. | *Escovopsis* sp. | LESF 318 | ES029 | Palmas, Tocantins, Brazil | Fungus garden of *Acromyrmex* sp. | KM817139 | Meirelles et al. (2015b) |
| *Escovopsis* sp. | *Escovopsis* sp. | LESF 047 | SES002 | Brasil, Goias, Brazil | Fungus garden of *Trachymyrmex* sp. | KM817147 | Meirelles et al. (2015b) |
| *Escovopsis* sp. | *Escovopsis* sp. | LESF 135 | SES003 | Uberlândia, Minas Gerais, Brazil | Fungus garden of *Trachymyrmex* sp. | KM817148 | Meirelles et al. (2015b) |
| *Escovopsis* sp. | *Escovopsis* sp. | LESF 048 | SES005 | Uberlândia, Minas Gerais, Brazil | Fungus garden of *Trachymyrmex* sp. | KF240731 | Meirelles et al. (2015b) |
| *Escovopsis* sp. | *Escovopsis* sp. | LESF 051 | SES009 | Palmeiras, Bahia, Brazil | Fungus garden of *Trachymyrmex* sp. | KM817153 | Meirelles et al. (2015b) |
| *Escovopsis* sp. | *Escovopsis* sp. | LESF 105 | SES004 | Uberlândia, Minas Gerais, Brazil | Fungus garden of *Sericomyrmex luederwaldti* | KM817149 | Meirelles et al. (2015b) |
| *Escovopsis* sp. | *Escovopsis* sp. | UT001 |  | Guadeloupe | Fungus garden of *Acromyrmex octospinosus* | KM817155 | Meirelles et al. (2015b) |
| *Escovopsis* sp. | *Escovopsis* sp. | UT002 |  | Guadeloupe | Fungus garden of *Acromyrmex* sp. | KM817156 | Meirelles et al. (2015b) |
| *Escovopsis* sp. | *Escovopsis* sp. | UT003 |  | Panama | Fungus garden of *Acromyrmex* sp. | KM817157 | Meirelles et al. (2015b) |
| *Escovopsis* sp. | *Escovopsis* sp. | UT004 |  | Panama | Fungus garden of *Acromyrmex colombica* | KM817158 | Meirelles et al. (2015b) |
| *Escovopsis* sp. | *Escovopsis* sp. | UT005 |  | Argentina | Fungus garden of *Acromyrmex* sp. | KM817159 | Meirelles et al. (2015b) |
| *Escovopsis* sp. | *Escovopsis* sp. | UT006 |  | Panama | Fungus garden of *Atta cephalotes* | KM817160 | Meirelles et al. (2015b) |
| *Escovopsis* sp. | *Escovopsis* sp. | UT007 |  | Panama | Fungus garden of *Acromyrmex colombica* | KM817161 | Meirelles et al. (2015b) |
| *Escovopsis* sp. | *Escovopsis* sp. | UT008 |  | Panama | Fungus garden of *Acromyrmex colombica* | KM817162 | Meirelles et al. (2015b) |
| *Escovopsis* sp. | *Escovopsis* sp. | UT009 |  | Panama | Fungus garden of *Acromyrmex colombica* | KM817163 | Meirelles et al. (2015b) |
| *Escovopsis* sp. | *Escovopsis* sp. | UT010 |  | Panama | Fungus garden of *Atta sexdens* | KM817164 | Meirelles et al. (2015b) |
| *Escovopsis* sp. | *Escovopsis* sp. | UT011 |  | Panama | Fungus garden of *Trachymyrmex* sp. | KM817165 | Meirelles et al. (2015b) |
| *Escovopsis* sp. | *Escovopsis* sp. | UT012 |  | Panama | Fungus garden of *Trachymyrmex* sp. | KM817166 | Meirelles et al. (2015b) |
| *Escovopsis* sp. | *Escovopsis* sp. | UT014 |  | Panama | Fungus garden of *Acromyrmex colombica* | KM817167 | Meirelles et al. (2015b) |
| *Escovopsis* sp. | *Escovopsis* sp. | UT015 |  | Panama | Fungus garden of *Acromyrmex colombica* | KM817168 | Meirelles et al. (2015b) |
| *Escovopsis* sp. | *Escovopsis* sp. | UT016 |  | Mexico | Fungus garden of *Trachymyrmex* sp. | KM817169 | Meirelles et al. (2015b) |
| *Escovopsis* sp. | *Escovopsis* sp. | UT017 |  | Panama | Fungus garden of *Acromyrmex colombica* | KM817170 | Meirelles et al. (2015b) |
| *Escovopsis* sp. | *Escovopsis* sp. | UT018 |  | Panama | Fungus garden of *Trachymyrmex* sp. | KM817171 | Meirelles et al. (2015b) |
| *Escovopsis* sp. | *Escovopsis* sp. | UT019 |  | Mexico | Fungus garden of *Atta cephalotes* | KM817172 | Meirelles et al. (2015b) |
| *Escovopsis* sp. | *Escovopsis* sp. | LESF892 | UT020 | Mexico | Fungus garden of *Trachymyrmex* sp. | KM817173 | Meirelles et al. (2015b) |
| *Escovopsis* sp. (as "Brown *Escovopsis*") | *Escovopsis* sp. | nmg011027-02 | g101_den_plr_brown | Panama | Fungus garden of *Apterostigma dentigerum* | AY629396 | Gerardo et al. (2004) |
| *Escovopsis* sp. (as "Brown *Escovopsis*") | *Escovopsis* sp. | sp011112-01 | g106_den_plr_brown | Panama | Fungus garden of *Apterostigma dentigerum* | AY629397 | Gerardo et al. (2004) |
| *Escovopsis* sp. cc020605-04 esc4 (as "Brown *Escovopsis*") | *Escovopsis* sp. | CC020605-04 esc4 |  | Panama | Fungus garden of *Apterostigma* sp. | DQ848163 | Gerardo et al. (2006) |
| *Escovopsis* sp. AGH020629-02 esc4 (as "Brown *Escovopsis*") | *Escovopsis* sp. | AGH020629-02 esc4 |  | Costa Rica | Fungus garden of *Apterostigma dentigerum* | DQ848161 | Gerardo et al. (2006) |
| *Escovopsis* sp. UGM020531-01 esc2 (as "Brown *Escovopsis*") | *Escovopsis* sp. | UGM020531-01 esc2 |  | Panama | Fungus garden of *Apterostigma dentigerum* | DQ848183 | Gerardo et al. (2006) |
| *Escovopsis* sp. AGH030627-08 esc1 (as "Brown *Escovopsis*") | *Escovopsis* sp. | AGH030627-08 esc1 |  | Ecuador | Fungus garden of *Apterostigma* sp. | DQ848182 | Gerardo et al. (2006) |
| *Escovopsis* sp. AGH030609-03 esc1 (as "Brown *Escovopsis*") | *Escovopsis* sp. | AGH030609-03 esc1 |  | Ecuador | Fungus garden of *Apterostigma* sp. | DQ848194 | Gerardo et al. (2006) |
| *Escovopsis* sp. AGH020709-10 esc11 (as "Brown *Escovopsis*") | *Escovopsis* sp. | AGH020709-10 esc11 |  | Costa Rica | Fungus garden of *Apterostigma dentigerum* | DQ848170 | Gerardo et al. (2006) |
| *Escovopsis* sp. sp011112-01 esc11 (as "Brown *Escovopsis*") | *Escovopsis* sp. | SP011112-01 esc11 |  | Panama | Fungus garden of *Apterostigma dentigerum* | DQ848180 | Gerardo et al. (2006) |
| *Escovopsis* sp. nmg010816-05 esc1 (as "Brown *Escovopsis*") | *Escovopsis* sp. | NMG010816-05 esc1 |  | Panama | Fungus garden of *Apterostigma dentigerum* | DQ848157 | Gerardo et al. (2006) |
| *Escovopsis* sp. cc011018-04 esc1 (as "Brown *Escovopsis*") | *Escovopsis* sp. | CC011018-04 esc1 |  | Panama | Fungus garden of *Apterostigma* sp. | DQ848177 | Gerardo et al. (2006) |
| *Escovopsis* sp. sv030614-02 esc1 (as "Brown *Escovopsis*") | *Escovopsis* sp. | SV030614-02 esc1 |  | Ecuador | Fungus garden of *Apterostigma* cf. *pilosum* | DQ848186 | Gerardo et al. (2006) |
| *Escovopsis* sp. AGH030627-01 esc1 (as "Brown *Escovopsis*") | *Escovopsis* sp. | AGH030627-01 esc1 |  | Ecuador | Fungus garden of *Apterostigma* cf. *dentigerum* | DQ848196 | Gerardo et al. (2006) |
| *Escovopsis* sp. AGH030627-03 esc2 (as "Brown *Escovopsis*") | *Escovopsis* sp. | AGH030627-03 esc2 |  | Ecuador | Fungus garden of *Apterostigma* sp. | DQ848197 | Gerardo et al. (2006) |
| *Escovopsis* sp. nmg020611-02 esc2 (as "Brown *Escovopsis*") | *Escovopsis* sp. | nmg020611-02 esc2 |  | Panama | Fungus garden of *Apterostigma dentigerum* | DQ848164 | Gerardo et al. (2006) |
| *Escovopsis* sp. UGM030106-02 escc (as "Brown *Escovopsis*") | *Escovopsis* sp. | UGM030106-02 escc |  | Panama | Fungus garden of *Apterostigma* sp. | DQ848188 | Gerardo et al. (2006) |
| *Escovopsis* sp. cc030106-02 escb (as "Brown *Escovopsis*") | *Escovopsis* sp. | cc030106-02 escb |  | Panama | Fungus garden of *Apterostigma* sp. | DQ848207 | Gerardo et al. (2006) |
| *Escovopsis* sp. abs020621-02 esc1 (as "Brown *Escovopsis*") | *Escovopsis* sp. | abs020621-02 esc1 |  | Costa Rica | Fungus garden of *Apterostigma dentigerum* | DQ848166 | Gerardo et al. (2006) |
| *Escovopsis* sp. nmg031215-04 (as "Brown *Escovopsis*") | *Escovopsis* sp. | nmg031215-04 |  | Panama | Fungus garden of *Apterostigma* sp. | DQ848204 | Gerardo et al. (2006) |
| *Escovopsis* sp. AGH030222-12 (as "Brown *Escovopsis*") | *Escovopsis* sp. | AGH030222-12 |  | Costa Rica | Fungus garden of *Apterostigma dentigerum* | DQ848187 | Gerardo et al. (2006) |
| *Escovopsis* sp. AGH020709-10 esc1 (as "Brown *Escovopsis*") | *Escovopsis* sp. | AGH020709-10 esc1 |  | Costa Rica | Fungus garden of *Apterostigma dentigerum* | DQ848169 | Gerardo et al. (2006) |
| *Escovopsis* sp. AGH020706-01 (as "Brown *Escovopsis*") | *Escovopsis* sp. | AGH020706-01 |  | Costa Rica | Fungus garden of *Apterostigma dentigerum* | DQ848168 | Gerardo et al. (2006) |
| *Escovopsis* sp. AGH020712-04 esc1 (as "Brown *Escovopsis*") | *Escovopsis* sp. | AGH020712-04 esc1 |  | Costa Rica | Fungus garden of *Apterostigma dentigerum* | DQ848173 | Gerardo et al. (2006) |
| *Escovopsis* sp. AGH020629-02 esc6 (as "Brown *Escovopsis*") | *Escovopsis* sp. | AGH020629-02 esc6 |  | Costa Rica | Fungus garden of *Apterostigma dentigerum* | DQ848167 | Gerardo et al. (2006) |
| *Escovopsis* sp. UGM020531-04 esc1 (as "Brown *Escovopsis*") | *Escovopsis* sp. | UGM020531-04 esc1 |  | Panama | Fungus garden of *Apterostigma* sp. | DQ848192 | Gerardo et al. (2006) |
| *Escovopsis* sp. AGH031215-02 (as "Brown *Escovopsis*") | *Escovopsis* sp. | AGH031215-02 |  | Panama | Fungus garden of *Apterostigma* sp. | DQ848203 | Gerardo et al. (2006) |
| *Escovopsis* sp. AGH020709-10 esc3 (as "Brown *Escovopsis*") | *Escovopsis* sp. | AGH020709-10 esc3 |  | Costa Rica | Fungus garden of *Apterostigma dentigerum* | DQ848171 | Gerardo et al. (2006) |
| *Escovopsis* sp. AGH020709-10 esc8 (as "Brown *Escovopsis*") | *Escovopsis* sp. | AGH020709-10 esc8 |  | Costa Rica | Fungus garden of *Apterostigma dentigerum* | DQ848172 | Gerardo et al. (2006) |
| *Escovopsis* sp. cc030327-01 esc4 (as "Brown *Escovopsis*") | *Escovopsis* sp. | cc030327-01 esc4 |  | Argentina | Fungus garden of *Apterostigma* sp. | DQ848201 | Gerardo et al. (2006) |
| *Escovopsis* sp. AGH020630-01 esc1 (as "Brown *Escovopsis*") | *Escovopsis* sp. | AGH020630-01 esc1 |  | Costa Rica | Fungus garden of *Apterostigma dentigerum* | DQ848176 | Gerardo et al. (2006) |
| *Escovopsis trichodermoides* | *Luteomyces trichodermoides* | LESF 310 | AR14022604A1 | Florianópolis, Santa Catarina, Brazil | Fungus garden of *Mycetophylax morschi* | MH724262 | Montoya et al. (2019) |
| *Escovopsis trichodermoides* | *Luteomyces trichodermoides* | LESF 311 | AR14022604A2 | Florianópolis, Santa Catarina, Brazil | Fungus garden of *Mycetophylax morschi* | MH724263 | Montoya et al. (2019) |
| *Escovopsis trichodermoides* | *Luteomyces trichodermoides* | LESF 312 | AR14022604ALA | Florianópolis, Santa Catarina, Brazil | Fungus garden of *Mycetophylax morschi* | MH724264 | Montoya et al. (2019) |
| *Escovopsis trichodermoides* | *Luteomyces trichodermoides* | LESF 832 | 13I3 | Rio Claro, São Paulo, Brazil | Fungus garden of *Mycocepurus smithii* | MT305366 | This study |
| *Escovopsis trichodermoides* | *Luteomyces trichodermoides* | LESF 833 | 13I1 | Rio Claro, São Paulo, Brazil | Fungus garden of *Mycocepurus smithii* | MT305367 | This study |
| *Escovopsis trichodermoides* | *Luteomyces trichodermoides* | LESF 834 | 13I2 | Rio Claro, São Paulo, Brazil | Fungus garden of *Mycocepurus smithii* | MT305368 | This study |
| *Escovopsis trichodermoides* | *Luteomyces trichodermoides* | LESF 835 | 3I1 | Rio Claro, São Paulo, Brazil | Midden of *Mycocepurus smithii* | MT305369 | This study |
| *Escovopsis trichodermoides* | *Luteomyces trichodermoides* | LESF 837 | 2I2 | Rio Claro, São Paulo, Brazil | Fungus garden of *Mycocepurus smithii* | MT305370 | This study |
| *Escovopsis trichodermoides* | *Luteomyces trichodermoides* | LESF 838 | 2I3 | Rio Claro, São Paulo, Brazil | Fungus garden of *Mycocepurus smithii* | MT305371 | This study |
| *Escovopsis trichodermoides* | *Luteomyces trichodermoides* | LESF 895 | Q03I | Botucatu, São Paulo, Brazil | Fungus garden of *Mycocepurus goeldii* | MT305380 | This study |
| *Escovopsis trichodermoides* | *Luteomyces trichodermoides* | LESF 897 | Q03III | Botucatu, São Paulo, Brazil | Fungus garden of *Mycocepurus goeldii* | MT305381 | This study |
| *Escovopsis trichodermoides* | *Luteomyces trichodermoides* | LESF 927 | Q23III | Botucatu, São Paulo, Brazil | Fungus garden of *Mycocepurus goeldii* | MT305383 | This study |
| *Escovopsis trichodermoides* | *Luteomyces trichodermoides* | LESF 1049 | QVM177 | Botucatu, São Paulo, Brazil | Fungus garden of *Mycocepurus goeldii* | MT305401 | This study |
| *Escovopsis trichodermoides* | *Luteomyces trichodermoides* | LESF 1051 | QVM179 | Botucatu, São Paulo, Brazil | Fungus garden of *Mycocepurus goeldii* | MT305402 | This study |
| *Escovopsis trichodermoides* | *Luteomyces trichodermoides* | LESF 1052 | QVM180 | Botucatu, São Paulo, Brazil | Fungus garden of *Mycocepurus goeldii* | MT305403 | This study |
| *Escovopsis trichodermoides* | *Luteomyces trichodermoides* | LESF 1055 | QVM183 | Botucatu, São Paulo, Brazil | Fungus garden of *Mycocepurus goeldii* | MT305404 | This study |
| *Escovopsis trichodermoides* | *Luteomyces trichodermoides* | LESF 1057 | QVM185 | Botucatu, São Paulo, Brazil | Fungus garden of *Mycocepurus goeldii* | MT305405 | This study |
| *Escovopsis trichodermoides* | *Luteomyces trichodermoides* | LESF 1061 | QVM189 | Botucatu, São Paulo, Brazil | Fungus garden of *Mycocepurus goeldii* | MT305406 | This study |
| *Escovopsis trichodermoides* | *Luteomyces trichodermoides* | LESF 1077 | QVM205 | Botucatu, São Paulo, Brazil | Fungus garden of *Mycocepurus goeldii* | MT305407 | This study |
| *Escovopsis trichodermoides* | *Luteomyces trichodermoides* | LESF 1078 | QVM206 | Botucatu, São Paulo, Brazil | Fungus garden of *Mycocepurus goeldii* | MT305408 | This study |
| *Escovopsis trichodermoides* | *Luteomyces trichodermoides* | LESF 1082 | QVM210 | Botucatu, São Paulo, Brazil | Fungus garden of *Mycocepurus goeldii* | MT305409 | This study |
| *Escovopsis trichodermoides* | *Luteomyces trichodermoides* | LESF 1090 | QVM218 | Botucatu, São Paulo, Brazil | Fungus garden of *Mycocepurus goeldii* | MT305410 | This study |
| *Escovopsis trichodermoides* | *Luteomyces trichodermoides* | LESF 1109 | QVM237 | Botucatu, São Paulo, Brazil | Fungus garden of *Mycocepurus goeldii* | MT305411 | This study |
| *Escovopsis trichodermoides* | *Luteomyces trichodermoides* | CBS 137343 ^ET^ | VEM001 | Rio Claro, Sao Paulo, Brazil | Fungus garden of *Mycocepurus goeldii* | KF033128 | Masiulionis et al. (2015),  Osti and Rodrigues (2018) |
| *Escovopsioides nivea* | *Escovopsioides nivea* | CBS 135749^ET^ | AUJ6 | Viçosa, Minas Gerais, Brazil | Fungus garden of *Acromyrmex subterraneus subterraneus* | JQ855713 | Augustin et al. (2013) |
| *Escovopsioides* sp. | *Escovopsioides nivea* | LESF 159 | J08 | Corumbataí, São Paulo, Brazil | Fungus garden of *Atta sexdens rubropilosa* | MF140949 | Osti and Rodrigues (2018) |
| *Escovopsioides* sp. | *Escovopsioides nivea* | LESF 601 | J09 | Rio Claro, São Paulo, Brazil | Fungus garden of *Trachymyrmex* sp. | MF140964 | Osti and Rodrigues (2018) |
| *Escovopsioides* sp. | *Escovopsioides nivea* | LESF 151 | J02 | Corumbataí, São Paulo, Brazil | Fungus garden of *Atta sexdens rubropilosa* | MF140948 | Osti and Rodrigues (2018) |
| *Escovopsioides* sp. | *Escovopsioides nivea* | LESF 510 | J10 | Botucatu, São Paulo, Brazil | Fungus garden of *Atta sexdens rubropilosa* | MF140950 | Osti and Rodrigues (2018) |
| *Escovopsioides* sp. | *Escovopsioides nivea* | LESF 587 | J01 | Camacan, Bahia, Brazil | Fungus garden of *Atta cephalotes* | MF140951 | Osti and Rodrigues (2018) |
| *Escovopsioides* sp. | *Escovopsioides nivea* | LESF 588 | J03 | Camacan, Bahia, Brazil | Fungus garden of *Atta cephalotes* | MF140952 | Osti and Rodrigues (2018) |
| *Escovopsioides* sp. | *Escovopsioides nivea* | LESF 589 | J05 | Camacan, Bahia, Brazil | Fungus garden of *Atta cephalotes* | MF140953 | Osti and Rodrigues (2018) |
| *Escovopsioides* sp. | *Escovopsioides nivea* | LESF 590 | J06 | Camacan, Bahia, Brazil | Fungus garden of *Atta cephalotes* | MF140954 | Osti and Rodrigues (2018) |
| *Escovopsioides* sp. | *Escovopsioides nivea* | LESF 591 | J04 | Botucatu, São Paulo, Brazil | Fungus garden of *Atta capiguara* | MF140955 | Osti and Rodrigues (2018) |
| *Escovopsioides* sp. | *Escovopsioides nivea* | LESF 592 | J12 | Camacan, Bahia, Brazil | Fungus garden of *Acromyrmex* sp. | MF140956 | Osti and Rodrigues (2018) |
| *Escovopsioides* sp. | *Escovopsioides nivea* | LESF 596 | J16 | Chuvisca, Rio Grande do Sul, Brazil | Fungus garden of *Acromyrmex* sp. | MF140960 | Osti and Rodrigues (2018) |
| *Escovopsioides* sp. | *Escovopsioides nivea* | LESF 597 | J17 | Camacan, Bahia, Brazil | Fungus garden of *Atta cephalotes* | MF140961 | Osti and Rodrigues (2018) |
| *Escovopsioides* sp. | *Escovopsioides nivea* | LESF 598 | J18 | Camacan, Bahia, Brazil | Fungus garden of *Atta cephalotes* | MF140962 | Osti and Rodrigues (2018) |
| *Escovopsioides* sp. | *Escovopsioides nivea* | LESF 599 | J19 | Sentinela do Sul, Rio Grande do Sul, Brazil | Fungus garden of *Acromyrmex heyeri* | MF140963 | Osti and Rodrigues (2018) |
| *Escovopsioides* sp. | *Escovopsioides nivea* | LESF 1009 | QVM137 | Manaus, Amazonas, Brazil | Fungus garden of *Apterostigma* sp. | MT305392 | This study |
| *Escovopsioides* sp. | *Escovopsioides nivea* | LESF 1023 | QVM151 | Manaus, Amazonas, Brazil | Fungus garden of *Trachymyrmex* sp. | MT305394 | This study |
| *Escovopsioides* sp. | *Escovopsioides nivea* | LESF 1025 | QVM153 | Manaus, Amazonas, Brazil | Fungus garden of *Trachymyrmex* sp. | MT305395 | This study |
| *Escovopsioides* sp. | *Escovopsioides nivea* | LESF 1028 | QVM156 | Manaus, Amazonas, Brazil | Fungus garden of *Trachymyrmex* sp. | MT305396 | This study |
| *Escovopsioides* sp. | *Escovopsioides nivea* | LESF 1031 | QVM159 | Manaus, Amazonas, Brazil | Fungus garden of *Trachymyrmex* sp. | MT305397 | This study |
| *Escovopsioides* sp. | *Escovopsioides nivea* | LESF 1039 | QVM167 | Manaus, Amazonas, Brazil | Fungus garden of *Cyphomyrmex* sp*.* | MT305398 | This study |
| *Escovopsioides* sp. | *Escovopsioides nivea* | LESF 1040 | QVM168 | Manaus, Amazonas, Brazil | Fungus garden of *Apterostigma* sp. | MT305399 | This study |
| *Escovopsioides* sp. | *Escovopsioides nivea* | LESF 1041 | QVM169 | Manaus, Amazonas, Brazil | Fungus garden of *Apterostigma* sp. | MT305400 | This study |
| *Escovopsis kreiselii* | *Sympodiorosea kreiselii* | CBS 139320 ^ET^ | LESF 053 | Florianópolis, Santa Catarina, Brazil | Fungus garden of *Mycetophylax morschi* | KJ 808766 | Meirelles et al. (2015a) |
| *Escovopsis kreiselii* | *Sympodiorosea kreiselii* | LESF 302 | AR14022705 | Florianópolis, Santa Catarina, Brazil | Fungus garden of *Mycetophylax morschi* | MH724259 | Montoya et al. (2019) |
| *Escovopsis kreiselii* | *Sympodiorosea kreiselii* | LESF 303 | AR14022705B | Florianópolis, Santa Catarina, Brazil | Fungus garden of *Mycetophylax morschi* | MH724260 | Montoya et al. (2019) |
| *Escovopsis kreiselii* | *Sympodiorosea kreiselii* | LESF 304 | AR14022705T2D | Florianópolis, Santa Catarina, Brazil | Fungus garden of *Mycetophylax morschi* | MH724261 | Montoya et al. (2019) |
| *Escovopsis kreiselii* | *Sympodiorosea kreiselii* | LESF 305 | AR14022601 | Florianópolis, Santa Catarina, Brazil | Fungus garden of *Mycetophylax morschi* | MT305353 | This study |
| *Escovopsis kreiselii* | *Sympodiorosea kreiselii* | LESF 306 | AR14022705A | Florianópolis, Santa Catarina, Brazil | Fungus garden of *Mycetophylax morschi* | MT305354 | This study |
| *Escovopsis kreiselii* | *Sympodiorosea kreiselii* | LESF 307 | AR14022705T2 | Florianópolis, Santa Catarina, Brazil | Fungus garden of *Mycetophylax morschi* | MT305355 | This study |
| *Escovopsis kreiselii* | *Sympodiorosea kreiselii* | LESF 308 | AR14022604AL | Florianópolis, Santa Catarina, Brazil | Fungus garden of *Mycetophylax morschi* | MT305356 | This study |
| *Escovopsis kreiselii* | *Sympodiorosea kreiselii* | LESF 309 | AR14022605T2 | Florianópolis, Santa Catarina, Brazil | Fungus garden of *Mycetophylax morschi* | MT305357 | This study |
| *Escovopsis* sp. | *Sympodiorosea* sp. | LESF 864 | SES030331-05 |  |  | MT305375 | This study |
| *Escovopsis* sp. | *Sympodiorosea* sp. | LESF 886 | UGM23 |  |  | MT305378 | This study |
| *Escovopsis* sp. | *Sympodiorosea* sp. | LESF 887 | UGM26(C) |  |  | MT305379 | This study |
| *Escovopsis* sp. | *Sympodiorosea* sp. | LESF 899 | Q03V | Botucatu, São Paulo, Brazil | Fungus garden of *Mycocepurus goeldii* | MT305382 | This study |
| *Escovopsis* sp. | *Sympodiorosea* sp. | LESF 1010 | QVM138 | Manaus, Amazonas, Brazil | Fungus garden of *Apterostigma* sp. | MT305393 | This study |
| *Escovopsis* sp. MT1 | *Sympodiorosea* sp. | MT1 |  | Panama | Fungus garden of *Mycocepurus tardus* | AY172627 | Currie et al. (2003) |
| *Escovopsis* sp. CC1 | *Sympodiorosea* sp. | CC1 |  | Panama | Fungus garden of *Cyphomyrmex costatus* | AY172624 | Currie et al. (2003) |
| *Escovopsis* sp. CC4 | *Sympodiorosea* sp. | CC4 |  | Panama | Fungus garden of *Cyphomyrmex costatus* | AY172625 | Currie et al. (2003) |
| *Escovopsis* sp. Esc10 | *Sympodiorosea* sp. | Esc10 |  | Panama | Fungus garden of *Myrmicocrypta ednaella* | AY172628 | Currie et al. (2003) |
| *Escovopsis* sp. Esc19 | *Sympodiorosea* sp. | Esc19 |  | Ecuador | Fungus garden of *Cyphomyrmex faunulus* | AY172626 | Currie et al. (2003) |
| *Escovopsis* sp. 10-20 | *Sympodiorosea* sp. | 10-20 |  | Guyana | Fungus garden of *Myrmicocrypta* sp. | AY172629 | Currie et al. (2003) |
| *Escovopsis* sp. (as "Pink *Escovopsis*") | *Sympodiorosea* sp. | nmg011101-04 | e48_lon_plr_un | Panama | Fungus garden of *Cyphomyrmex longiscapus* | AY629361 | Gerardo et al. (2004) |
| *Escovopsis* sp. (as "Pink *Escovopsis*") | *Sympodiorosea* sp. | nmg011101-12 | h4_lon_plr_un | Panama | Fungus garden of *Cyphomyrmex longiscapus* | AY629362 | Gerardo et al. (2004) |
| *Escovopsis* sp. (as "Pink *Escovopsis*") | *Sympodiorosea* sp. | rmma010321-19 | g021_lon_plr_pink | Panama | Fungus garden of *Cyphomyrmex longiscapus* | AY629363 | Gerardo et al. (2004) |
| *Escovopsis* sp. (as "Pink *Escovopsis*") | *Sympodiorosea* sp. | nmg011102-03 | g074_lon_plr_pink | Panama | Fungus garden of *Cyphomyrmex longiscapus* | AY629364 | Gerardo et al. (2004) |
| *Escovopsis* sp. (as "Pink *Escovopsis*") | *Sympodiorosea* sp. | nmg010808-03 | g144_lon_plr_pink | Panama | Fungus garden of *Cyphomyrmex longiscapus* | AY629365 | Gerardo et al. (2004) |
| *Escovopsis* sp. (as "Pink *Escovopsis*") | *Sympodiorosea* sp. | nmg010816-01 | g038_lon_plr_pink | Panama | Fungus garden of *Cyphomyrmex longiscapus* | AY629366 | Gerardo et al. (2004) |
| *Escovopsis* sp. (as "Pink *Escovopsis*") | *Sympodiorosea* sp. | nmg011114-03 | g043_lon_el_pink | Panama | Fungus garden of *Cyphomyrmex longiscapus* | AY629367 | Gerardo et al. (2004) |
| *Escovopsis* sp. (as "Pink *Escovopsis*") | *Sympodiorosea* sp. | cc011114-02 | g071_lon_el_pink | Panama | Fungus garden of *Cyphomyrmex longiscapus* | AY629368 | Gerardo et al. (2004) |
| *Escovopsis* sp. (as "Pink *Escovopsis*") | *Sympodiorosea* sp. | cc011120-03 | e44_cos_ga_pink | Panama | Fungus garden of *Cyphomyrmex costatus* | AY629369 | Gerardo et al. (2004) |
| *Escovopsis* sp. (as "Pink *Escovopsis*") | *Sympodiorosea* sp. | sp011105-01 | g042_cos_bci_pink | Panama | Fungus garden of *Cyphomyrmex costatus* | AY629370 | Gerardo et al. (2004) |
| *Escovopsis* sp. (as "Pink *Escovopsis*") | *Sympodiorosea* sp. | ugm010407-27 | i10_mue_plr_pink | Panama | Fungus garden of *Cyphomyrmex muelleri* | AY629371 | Gerardo et al. (2004) |
| *Escovopsis* sp. (as "Pink *Escovopsis*") | *Sympodiorosea* sp. | ugm010323-01 | e54_cos_un_pink | Panama | Fungus garden of *Cyphomyrmex costatus* | AY629372 | Gerardo et al. (2004) |
| *Escovopsis* sp. (as "Pink *Escovopsis*") | *Sympodiorosea* sp. | cc011124-03 | e45_cos_plr_pink | Panama | Fungus garden of *Cyphomyrmex costatus* | AY629373 | Gerardo et al. (2004) |
| *Escovopsis* sp. (as "Pink *Escovopsis*") | *Sympodiorosea* sp. | cc011211-02 | i12_cos_plr_pin | Panama | Fungus garden of *Cyphomyrmex costatus* | AY629374 | Gerardo et al. (2004) |
| *Escovopsis* sp. (as "Pink *Escovopsis*") | *Sympodiorosea* sp. | cc011205-06 | e46_cos_plr_pin | Panama | Fungus garden of *Cyphomyrmex costatus* | AY629375 | Gerardo et al. (2004) |
| *Escovopsis* sp. (as "Pink *Escovopsis*") | *Sympodiorosea* sp. | sp011108-03 | g089_mue_ft_pin | Panama | Fungus garden of *Cyphomyrmex muelleri* | AY629376 | Gerardo et al. (2004) |
| *Escovopsis* sp. (as "Pink *Escovopsis*") | *Sympodiorosea* sp. | cc011211-03 | e47_cos_plr_pink | Panama | Fungus garden of *Cyphomyrmex costatus* | AY629377 | Gerardo et al. (2004) |
| *Escovopsis* sp. (as "Pink *Escovopsis*") | *Sympodiorosea* sp. | cc011209-03 | h6_cos_plr_pink | Panama | Fungus garden of *Cyphomyrmex costatus* | AY629378 | Gerardo et al. (2004) |
| *Escovopsis* sp. (as "Pink *Escovopsis*") | *Sympodiorosea* sp. | 010614cc1 | e59_cos_un_pink | Panama | Fungus garden of *Cyphomyrmex costatus* | AY629379 | Gerardo et al. (2004) |
| *Escovopsis* sp. (as "Pink *Escovopsis*") | *Sympodiorosea* sp. | sp011108-02 | g086_mue_ft_pink | Panama | Fungus garden of *Cyphomyrmex muelleri* | AY629380 | Gerardo et al. (2004) |
| *Escovopsis* sp. (as "Pink *Escovopsis*") | *Sympodiorosea* sp. | nmg011110-07 | g082_cos_plr_pink | Panama | Fungus garden of *Cyphomyrmex costatus* | AY629381 | Gerardo et al. (2004) |
| *Escovopsis* sp. (as "Pink *Escovopsis*") | *Sympodiorosea* sp. | nmg011105-06 | g080_mue_bci_pink | Panama | Fungus garden of *Cyphomyrmex muelleri* | AY629382 | Gerardo et al. (2004) |
| *Escovopsis* sp. (as "Pink *Escovopsis*") | *Sympodiorosea* sp. | ugm010407-1 | i19_mue_plr_pink | Panama | Fungus garden of *Cyphomyrmex muelleri* | AY629383 | Gerardo et al. (2004) |
| *Escovopsis* sp. (as "Pink *Escovopsis*") | *Sympodiorosea* sp. | ugm010407-15 | i19_mue_plr_pink | Panama | Fungus garden of *Cyphomyrmex muelleri* | AY629384 | Gerardo et al. (2004) |
| *Escovopsis* sp. (as "Pink *Escovopsis*") | *Sympodiorosea* sp. | nmg011110-05 | e53_mue_plr_pink | Panama | Fungus garden of *Cyphomyrmex muelleri* | AY629385 | Gerardo et al. (2004) |
| *Escovopsis* sp. (as "Pink *Escovopsis*") | *Sympodiorosea* sp. | cc011213-10 | i7_mue_plr_pink | Panama | Fungus garden of *Cyphomyrmex muelleri* | AY629386 | Gerardo et al. (2004) |
| *Escovopsis* sp. (as "Pink *Escovopsis*") | *Sympodiorosea* sp. | nmg011101-11 | h10_mue_plr_pink | Panama | Fungus garden of *Cyphomyrmex muelleri* | AY629387 | Gerardo et al. (2004) |
| *Escovopsis* sp. (as "Pink *Escovopsis*") | *Sympodiorosea* sp. | ugm010407-11 | h8_mue_plr_pink | Panama | Fungus garden of *Cyphomyrmex muelleri* | AY629388 | Gerardo et al. (2004) |
| *Escovopsis* sp. (as "Pink *Escovopsis*") | *Sympodiorosea* sp. | ugm010407-12 | i11_mue_plr_pink | Panama | Fungus garden of *Cyphomyrmex muelleri* | AY629389 | Gerardo et al. (2004) |
| *Escovopsis* sp. (as "Pink *Escovopsis*") | *Sympodiorosea* sp. | nmg011101-06 | e52_mue_plr_pink | Panama | Fungus garden of *Cyphomyrmex muelleri* | AY629390 | Gerardo et al. (2004) |
| *Escovopsis* sp. (as "Pink *Escovopsis*") | *Sympodiorosea* sp. | cc011110-03 | g069_mue_plr_pink | Panama | Fungus garden of *Cyphomyrmex muelleri* | AY629391 | Gerardo et al. (2004) |
| *Escovopsis* sp. (as "Pink *Escovopsis*") | *Sympodiorosea* sp. | ugm010407-01 | e55_mue_plr_pink | Panama | Fungus garden of *Cyphomyrmex muelleri* | AY629392 | Gerardo et al. (2004) |
| *Escovopsis* sp. (as "Pink *Escovopsis*") | *Sympodiorosea* sp. | rmma010311-02 | g063_mue_plr_org | Panama | Fungus garden of *Cyphomyrmex muelleri* | AY629393 | Gerardo et al. (2004) |
| *Escovopsis* sp. (as "Pink *Escovopsis*") | *Sympodiorosea* sp. | nmg011105-03 | g078_mue_bci_pink | Panama | Fungus garden of *Cyphomyrmex muelleri* | AY629394 | Gerardo et al. (2004) |
| *Escovopsis* sp. CC010325-06 esc2 (as "Pink *Escovopsis*") | *Sympodiorosea* sp. | CC010325-06 esc2 |  | Panama | Fungus garden of *Apterostigma auriculatum* | DQ848181 | Gerardo et al. (2006) |
| *Escovopsis* sp. NMG031218-01 esc2 (as "Pink *Escovopsis*") | *Sympodiorosea* sp. | NMG031218-01 esc2 |  | Panama | Fungus garden of *Apterostigma auriculatum* | DQ848206 | Gerardo et al. (2006) |
| *Escovopsis* sp. CC011029-02 esc1 (as "Pink *Escovopsis*") | *Sympodiorosea* sp. | CC011029-02 esc1 |  | Panama | Fungus garden of *Apterostigma auriculatum* | DQ848178 | Gerardo et al. (2006) |
| *Escovopsis* sp. (as "yellow *Escovopsis*") | ? | cc011213-31 esc1 | --- | Panama | Fungus garden of *Apterostigma auriculatum* | DQ848179 | Gerardo et al. (2006) |
| *Escovopsis* sp. (as "yellow *Escovopsis*") | ? | nmg011101-03 | --- | Panama | Fungus garden of *Cyphomyrmex longiscapus* | DQ848209 | Gerardo et al. (2006) |
| *Escovopsis* sp. (as "white *Escovopsis*") | ? | nmg011027-02 | g109_den_plr_brown | Panama | Fungus garden of *Apterostigma dentigerum* | AY629395 | Gerardo et al. (2004) |
| *Escovopsis* sp. (as "white *Escovopsis*") | ? | sv030615-04 esc1 | --- | Ecuador | Fungus garden of *Apterostigma* sp. | DQ848199 | Gerardo et al. (2006) |
| *Escovopsis* sp. (as "white *Escovopsis*") | ? | sv030615-05 esc1 | --- | Ecuador | Fungus garden of *Apterostigma* sp. | DQ848200 | Gerardo et al. (2006) |
| *Escovopsis* sp. (as "white *Escovopsis*") | ? | nmg030614-01 esc1 | --- | Ecuador | Fungus garden of *Apterostigma* cf. *pilosum* | DQ848202 | Gerardo et al. (2006) |
| *Escovopsis* sp. (as "white *Escovopsis*") | ? | al030618-10 esc1 | --- | Ecuador | Fungus garden of *Apterostigma* sp. | DQ848189 | Gerardo et al. (2006) |
| *Escovopsis* sp. (as "white *Escovopsis*") | ? | al030609-03 esc1 | --- | Ecuador | Fungus garden of *Apterostigma* sp. | DQ848191 | Gerardo et al. (2006) |
| *Escovopsis* sp. (as "white *Escovopsis*") | ? | nmg020519-02 esc2 | --- | Panama | Fungus garden of *Apterostigma* cf. *pilosum* | DQ848185 | Gerardo et al. (2006) |
| *Escovopsis* sp. (as "white *Escovopsis*") | ? | nmg011029-03 esc1 | --- | Panama | Fungus garden of *Apterostigma* sp. | DQ848193 | Gerardo et al. (2006) |
| *Escovopsis* sp. (as "white *Escovopsis*") | ? | nmg010802-02 esc1 | --- | Panama | Fungus garden of *Apterostigma dentigerum* | DQ848156 | Gerardo et al. (2006) |
| *Escovopsis* sp. (as "white *Escovopsis*") | ? | sp011112-01 esc1 | --- | Panama | Fungus garden of *Apterostigma dentigerum* | DQ848162 | Gerardo et al. (2006) |
| *Escovopsis* sp. (as "white *Escovopsis*") | ? | nmg010816-05 esc19 | --- | Panama | Fungus garden of *Apterostigma dentigerum* | DQ848158 | Gerardo et al. (2006) |
| *Escovopsis* sp. (as "white *Escovopsis*") | ? | nmg020611-02 esc7 | --- | Panama | Fungus garden of *Apterostigma dentigerum* | DQ848175 | Gerardo et al. (2006) |
| *Escovopsis* sp. (as "white *Escovopsis*") | ? | ugm030327-05 esc4 | --- | Argentina | Fungus garden of *Apterostigma* sp. | DQ848208 | Gerardo et al. (2006) |
| *Escovopsis* sp. (as "white *Escovopsis*") | ? | nmg010318-21 esc2 | --- | Panama | Fungus garden of *Apterostigma dentigerum* | DQ848159 | Gerardo et al. (2006) |
| *Escovopsis* sp. (as "white *Escovopsis*") | ? | agh030618-02 esc1 | --- | Ecuador | Fungus garden of *Apterostigma* sp. | DQ848195 | Gerardo et al. (2006) |
| *Escovopsis* sp. (as "white *Escovopsis*") | ? | agh020621-05 esc2 | --- | Costa Rica | Fungus garden of *Apterostigma dentigerum* | DQ848160 | Gerardo et al. (2006) |
| *Escovopsis* sp. (as "white *Escovopsis*") | ? | ugm020602-07 esc1 | --- | Panama | Fungus garden of *Apterostigma* sp. | DQ848165 | Gerardo et al. (2006) |
| *Escovopsis* sp. (as "white *Escovopsis*") | ? | nmg020611-02 esc6 | --- | Panama | Fungus garden of *Apterostigma dentigerum* | DQ848174 | Gerardo et al. (2006) |
| *Escovopsis* sp. (as "white *Escovopsis*") | ? | nmg020521-04 esc1 | --- | Panama | Fungus garden of *Apterostigma dentigerum* | DQ848190 | Gerardo et al. (2006) |
| *Escovopsis* sp. (as "white *Escovopsis*") | ? | nmg030618-01 esc1 | --- | Ecuador | Fungus garden of *Apterostigma* cf. *pilosum* | DQ848184 | Gerardo et al. (2006) |
| *Escovopsis* sp. | ? | Esc26 | --- | Guyana | Fungus garden of *Apterostigma dorothea* | AY172619 | Currie et al. (2003) |
| *Escovopsis* sp. | ? | Esc20 | --- | Ecuador | Fungus garden of *Apterostigma* cf. *pilosum* | AY172618 | Currie et al. (2003) |
| *Cladobotryum asterophorum* | *Cladobotryum asterophorum* | CBS 676.77 |  |  |  | FN868712 | Poldmaa (2011) |
| *Cladobotryum cubitense* | *Cladobotryum cubitense* | CBS 416.85 |  |  |  | FN868713 | Poldmaa (2011) |
| *Cladobotryum heterosporum* | *Cladobotryum heterosporum* | CBS 719.88 |  |  |  | FN868716 | Poldmaa (2011) |
| *Cladobotryum multiseptatum* | *Cladobotryum multiseptatum* | CBS 472.71 |  |  |  | FN868723 | Poldmaa (2011) |
| *Cladobotryum paravirescens* | *Cladobotryum paravirescens* | TFC 97-23 |  |  |  | FN868724 | Poldmaa (2011) |
| *Cladobotryum protrusum* | *Cladobotryum protrusum* | CBS 118999 |  |  |  | FN868726 | Poldmaa (2011) |
| *Cladobotryum purpureum* | *Cladobotryum purpureum* | CBS 154.78 |  |  |  | FN868733 | Poldmaa (2011) |
| *Cladobotryum rubrobrunnescens* | *Cladobotryum rubrobrunnescens* | CBS 176.92 |  |  |  | FN868734 | Poldmaa (2011) |
| *Cladobotryum indoafricum* | *Cladobotryum indoafricum* | TFC 201295 |  |  |  | FN868721 | Poldmaa (2011) |
| *Cladobotryum tchimbelense* | *Cladobotryum tchimbelense* | TFC 201146 |  |  |  | FN868737 | Poldmaa (2011) |
| *Cladobotryum tenue* | *Cladobotryum tenue* | G.A. 05/54.K | TFC:05-91 | Germany | *Russula* sp. | HF911717 | Poldmaa (2011) |
| *Cladobotryum protrusum* | *Cladobotryum protrusum* | TFC 201316 |  | Madagascar | Eucalyptus forest | FN868732 | Põldmaa (2011) |
| *Hypomyces armeniacus* | *Hypomyces armeniacus* | TFC 02-86/2 |  |  |  | FN868742 | Põldmaa (2011) |
| *Hypomyces australasiaticus* | *Hypomyces australasiaticus* | TFC 99-95 |  |  |  | FN868745 | Põldmaa (2011) |
| *Hypomyces dactylarioide* | *Hypomyces dactylarioide* | CBS 141.78 |  |  |  | FN868748 | Põldmaa (2011) |
| *Hypomyces gabonensis* | *Hypomyces gabonensis* | TFC 201156 |  |  |  | FN868749 | Põldmaa (2011) |
| *Hypomyces khaoyaiensis* | *Hypomyces khaoyaiensis* | G.J.S. 01-304 |  |  |  | FN868750 | Põldmaa (2011) |
| *Hypomyces odoratus* | *Hypomyces odoratus* | Grogan 192B1 | IMI:372795 | United Kingdom | *Agaricus bisporus* | HF911639 | Tamm and Poldmaa (2013) |
| *Hypomyces odoratus* | *Hypomyces odoratus* | C.T.R. 72-23 |  |  |  | FN868752 | Põldmaa (2011) |
| *Hypomyces rosellus* | *Hypomyces rosellus* | TFC 01-25 |  |  |  | FN868760 | Põldmaa (2011) |
| *Hypomyces samuelsii* | *Hypomyces samuelsii* | TFC 2007-23 |  | Peru | on  basidioma of an agaricoid | FN868769 | Põldmaa (2011) |
| *Hypomyces semicirculare* | *Hypomyces semicirculare* | CBS 705. 88 |  | Cuba | On old polypore | FN868735 | Põldmaa (2011) |
| *Sphaerostilbella aureonitens* | *Sphaerostilbella aureonitens* | GJS 74-87 |  |  |  | FJ467644 | unpublished |
| *Sphaerostilbella berkeleyana* | *Sphaerostilbella berkeleyana* | CBS 102308  GJS 82-274 |  | New Zealand | polypore (Hymenomycetes) | AF543783 | Currie et al. (2003) |
| *Sphaerostilbella* cf. *aureonitens* | *Sphaerostilbella* cf. *aureonitens* | G.J.S. 74-87 |  | New Zealand |  | DQ834452 | Overton et al. (2006) |
| *Sphaerostilbella* cf. *aureonitens* | *Sphaerostilbella* cf. *aureonitens* | G.J.S. 82-40 |  | New Zealand |  | DQ834453 | Overton et al. (2006) |
| *Trichoderma atlanticum* | *Trichoderma atlanticum* | C.P.K. 1896 |  |  |  | FJ860648 | Jaklitsch et al. (2011) |
| *Trichoderma alni* | *Trichoderma alni* | CPK2854 |  |  |  | EU498314 | Jaklitsch et al. (2008b) |
| *Trichoderma americanum* | *Trichoderma americanum* | G.J.S. 94-79 |  |  |  | DQ835491 |  |
| *Trichoderma brunneoviride* | *Trichoderma brunneoviride* | CBS 120928 |  |  |  | EU498318 | Jaklitsch et al. (2008b) |
| *Trichoderma epimyces* | *Trichoderma epimyces* | CPK2487 |  |  |  | EU498322 | Jaklitsch et al. (2008b) |
| *Trichoderma harzianum* | *Trichoderma harzianum* | CBS 226.95 |  | England |  | AF534621 | Chaverri et al. (2003) |
| *Trichoderma hispanicum* | *Trichoderma hispanicum* | S419 |  |  |  | JN715657 | Jaklitsch et al. (2012) |
| *Trichoderma luteocrystallinum* | *Trichoderma luteocrystallinum* | CBS 123828 |  |  |  | FJ860646 | Jaklitsch et al. (2011) |
| *Trichoderma stercorarium* | *Trichoderma stercorarium* | CBS 148.85 |  |  |  | FJ860607 | Jaklitsch et al. (2011) |
| *Trichoderma stercorarium* | *Trichoderma stercorarium* | ATCC 62321 |  |  |  | AF543782 | Currie et al. (2003) |
| *Protocrea pallida* | *Protocrea pallida* | TFC 99-209 |  | New York, Cleaveland, USA |  | EU703903 | Jaklitsch et al. (2008a) |
| *Lecanicillium antillanum* | *Lecanicillium antillanum* | CBS 350.85 |  | Cuba | On basidioma of an agaricoid | DQ522350 | Spatafora et al. (2007) |

^?^ Strains treated as yellow and white *Escovopsis* in Currie et al. (2003) and Gerardo et al. (2006). No taxonomic studies were carried out with these strains and most likely represent new genera. LESF: Laboratory of Fungal Ecology and Systematics (UNESP, Rio Claro, Brazil).

Table S4. Strains and their associated metadata used to show the phylogenetic placement of *Escovopsis* species described by Marfetán et al. (2018) (Fig. S1).

| **Current fungal species name** | **Strain ID** | **Specimen voucher** | **City, State, Country** | **Coordinate** | **Habitat** | **GenBank accessions LSU** | **References** |
| --- | --- | --- | --- | --- | --- | --- | --- |
| *Escovopsioides nivea* | CBS 135749^ET^ | AUJ6 | Viçosa, Minas Gerais, Brazil | 20°44'31.71''S; 42°52 '43.83''W | Fungus garden of *Acromyrmex subterraneus subterraneus* | JQ855716 | Augustin et al. (2013) |
| *Escovopsioides nivea* | LESF 159 | J08 | Corumbataí, São Paulo, Brazil | 22°17'21.7''S; 47°39'22.8''W | Fungus garden of *Atta sexdens rubropilosa* | MF116034 | Osti and Rodrigues (2018) |
| *Escovopsioides nivea* | LESF 601 | J09 | Rio Claro, São Paulo, Brazil |  | Fungus garden of *Trachymyrmex* sp. | MF116049 | Osti and Rodrigues (2018) |
| *Escovopsioides nivea* | LESF 151 | J02 | Corumbataí, São Paulo, Brazil | 22°17'21.7''S; 47°39'22.8''W | Fungus garden of *Atta sexdens rubropilosa* | MF116033 | Osti and Rodrigues (2018) |
| *Escovopsioides nivea* | LESF 510 | J10 | Botucatu, São Paulo, Brazil | 22°54'28.4"S; 48°18'55.7"W | Fungus garden of *Atta sexdens rubropilosa* | MF116035 | Osti and Rodrigues (2018) |
| *Escovopsioides nivea* | LESF 587 | J01 | Camacan, Bahia, Brazil | 15°23'18.2"S; 39°33'30.5"W | Fungus garden of *Atta cephalotes* | MF116036 | Osti and Rodrigues (2018) |
| *Escovopsioides nivea* | LESF 588 | J03 | Camacan, Bahia, Brazil | 15°25'32.3"S 39°32'48.1"W | Fungus garden of *Atta cephalotes* | MF116037 | Osti and Rodrigues (2018) |
| *Escovopsioides nivea* | LESF 589 | J05 | Camacan, Bahia, Brazil | 15°23'14.8"S; 39°33'28.4"W | Fungus garden of *Atta cephalotes* | MF116038 | Osti and Rodrigues (2018) |
| *Escovopsioides nivea* | LESF 590 | J06 | Camacan, Bahia, Brazil | 15°23'15.2S; 39°33'28.0"W | Fungus garden of *Atta cephalotes* | MF116039 | Osti and Rodrigues (2018) |
| *Escovopsioides nivea* | LESF 591 | J04 | Botucatu, São Paulo, Brazil | 22°54'26.6"S; 48°18'29.2"W | Fungus garden of *Atta capiguara* | MF116040 | Osti and Rodrigues (2018) |
| *Escovopsioides nivea* | LESF 592 | J12 | Camacan, Bahia, Brazil | 15°22'50.3''S; 39°34'03.5''W | Fungus garden of *Acromyrmex* sp. | MF116041 | Osti and Rodrigues (2018) |
| *Escovopsioides nivea* | LESF 596 | J16 | Chuvisca, Rio Grande do Sul, Brazil | 30°50'10.2"S; 51°55'10.4"W | Fungus garden of *Acromyrmex* sp. | MF116045 | Osti and Rodrigues (2018) |
| *Escovopsioides nivea* | LESF 597 | J17 | Camacan, Bahia, Brazil | 15°23'29.7"S; 39°33'31.3"W | Fungus garden of *Atta cephalotes* | MF116046 | Osti and Rodrigues (2018) |
| *Escovopsioides nivea* | LESF 598 | J18 | Camacan, Bahia, Brazil | 15°23'17.8"S; 39 33'22.3"W | Fungus garden of *Atta cephalotes* | MF116047 | Osti and Rodrigues (2018) |
| *Escovopsioides nivea* | LESF 599 | J19 | Sentinela do Sul, Rio Grande do Sul, Brazil |  | Fungus garden of *Acromyrmex heyeri* | MF116048 | Osti and Rodrigues (2018) |
| *Escovopsioides nivea* | LESF 1009 | QVM137 | Manaus, Amazonas, Brazil | 2°26'55.3''S; 59°46'10.9''W | Fungus garden of *Apterostigma* sp. | MT273572 | This study |
| *Escovopsioides nivea* | LESF 1023 | QVM151 | Manaus, Amazonas, Brazil | 2°26'52.5''S; 59°45'53.4''W | Fungus garden of *Trachymyrmex* sp. | MT273574 | This study |
| *Escovopsioides nivea* | LESF 1025 | QVM153 | Manaus, Amazonas, Brazil | 2°26'52.5''S; 59°45'53.4''W | Fungus garden of *Trachymyrmex* sp. | MT273575 | This study |
| *Escovopsioides nivea* | LESF 1028 | QVM156 | Manaus, Amazonas, Brazil | 2°26'52.6''S; 59°45'52.4''W | Fungus garden of *Trachymyrmex* sp. | MT273576 | This study |
| *Escovopsioides nivea* | LESF 1031 | QVM159 | Manaus, Amazonas, Brazil | 2°26'55.5''S; 59°45'54.2''W | Fungus garden of *Trachymyrmex* sp. | MT273577 | This study |
| *Escovopsioides nivea* | LESF 1039 | QVM167 | Manaus, Amazonas, Brazil | 2°26'52.3''S; 59° 45'51.9''W | Fungus garden of *Cyphomyrmex* sp*.* | MT273578 | This study |
| *Escovopsioides nivea* | LESF 1040 | QVM168 | Manaus, Amazonas, Brazil | 2°26'52.6''S; 59°45'52.4''W | Fungus garden of *Apterostigma* sp. | MT273579 | This study |
| *Escovopsioides nivea* | LESF 1041 | QVM169 | Manaus, Amazonas, Brazil | 2°26'55.5''S; 59°45'54.2''W | Fungus garden of *Apterostigma* sp. | MT273580 | This study |
| *Escovopsis* *aspergilloides* | CBS 423.93 ^ET^ | DAOM:216382 | Trinidad and Tobago: Trinidad |  | Fungus garden of *Trachymyrmex ruthae* | KF293283 | Augustin et al. (2013) |
| *Escovopsis atlas* | UNQ E28 | E28 | Salta, Argentina | 25°13′59′′S; 64°42′58′′W | Fungus garden of *Acromyrmex lundii* | KU298288 | Marfetán et al. (2018) |
| *Escovopsis atlas* | UNQ E35 | E35 | Tucumán, Argentina | 7°13′51′′S; 65°54′47′′W | Fungus garden of *Acromyrmex aspersus* | KU298289 | Marfetán et al. (2018) |
| *Escovopsis catenulata* | UNQ E17 | E17 | Corrientes, Argentina | 29°11′53′′S; 58°02′28′′W | Fungus gardens of *Acromyrmex lobcornis* | KU298285 | Marfetán et al. (2018) |
| *Escovopsis catenulata* | UNQ E18 | E18 | Santa Fé, Argentina | 30°22′55′′S; 61°14′20′′W | Fungus garden of *Atta vollenweideri* | KU298295 | Marfetán et al. (2018) |
| *Escovopsis catenulata* | UNQ E19 | E19 | Santa Fé, Argentina | 30°22′57′′S; 61°14′16′′W | Fungus garden of *Acromyrmex heyeri* | KU298286 | Marfetán et al. (2018) |
| *Escovopsis catenulata* | UNQ E34 | E34 | Tucumán, Argentina | 7°13′48′′S; 65°54′51′′W | Fungus garden of *Acromyrmex aspersus* | KU298287 | Marfetán et al. (2018) |
| *Escovopsis clavata* | LESF 854 | 1704A | Florianópolis, Santa Catarina, Brazil | 27°44'38.94''S 48°31'9.3''W | Fungus garden of *Apterostigma* sp. | MH715111 | Montoya et al. (2019) |
| *Escovopsis clavata* | LESF 855 | 1705B | Florianópolis, Santa Catarina, Brazil | 27°44'39.49''S 48°31'9.72''W | Fungus garden of *Apterostigma* sp. | MH715112 | Montoya et al. (2019) |
| *Escovopsis clavata* | CBS 145326 ^ET^ | 1707 | Florianópolis, Santa Catarina, Brazil | 27°44'39.6''S 48°31'10.14''W | Fungus garden of *Apterostigma* sp. | MH715110 | Montoya et al. (2019) |
| *Sympodiorosea kreiselii* | LESF 302 | AR14022705 | Florianópolis, Santa Catarina, Brazil | 27°31'24.96''S  48°25'3.78''W | Fungus garden of *Mycetophylax morschi* | MH715099 | Montoya et al. (2019) |
| *Sympodiorosea kreiselii* | LESF 303 | AR14022705B | Florianópolis, Santa Catarina, Brazil | 27°31'24.96''S  48°25'3.78''W | Fungus garden of *Mycetophylax morschi* | MH715100 | Montoya et al. (2019) |
| *Sympodiorosea kreiselii* | LESF 304 | AR14022705T2D | Florianópolis, Santa Catarina, Brazil | 27°31'24.96''S  48°25'3.78''W | Fungus garden of *Mycetophylax morschi* | MH715101 | Montoya et al. (2019) |
| *Sympodiorosea kreiselii* | LESF 305 | AR14022601 | Florianópolis, Santa Catarina, Brazil | 27°37'49.6"S; 48°27'03.6"W | Fungus garden of *Mycetophylax morschi* | MT273524 | This study |
| *Sympodiorosea kreiselii* | LESF 306 | AR14022705A | Florianópolis, Santa Catarina, Brazil | 27°31'25.0"S; 48°25'03.8"W | Fungus garden of *Mycetophylax morschi* | MT273525 | This study |
| *Sympodiorosea kreiselii* | LESF 307 | AR14022705T2 | Florianópolis, Santa Catarina, Brazil | 27°31'25.0"S; 48°25'03.8"W | Fungus garden of *Mycetophylax morschi* | MT273526 | This study |
| *Sympodiorosea kreiselii* | LESF 308 | AR14022604AL | Florianópolis, Santa Catarina, Brazil | 27°37'49.6"S; 48°27'03.6"W | Fungus garden of *Mycetophylax morschi* | MT273527 | This study |
| *Sympodiorosea kreiselii* | LESF 309 | AR14022605T2 | Florianópolis, Santa Catarina, Brazil | 27°37'47.9"S; 48°27'04.0"W | Fungus garden of *Mycetophylax morschi* | MT273528 | This study |
| *Sympodiorosea kreiselii* | CBS 139320 ^ET^ | LESF 053 | Florianópolis, Santa Catarina, Brazil | 27°37'50.01''S 48°27'03.64''W | Fungus garden of *Mycetophylax morschi* | KJ808765 | Meirelles et al. (2015a) |
| *Escovopsis lentecrescens* | CBS 135750 ^ET^ | VIC:31755 | Viçosa, Minas Gerais, Brazil | 20°44'31.71''S 42°52'43.83''W | Fungus garden of *Acromyrmex subterraneus subterraneus* | JQ855717 | Augustin et al. (2013) |
| *Escovopsis microspora* | CBS 135751^ET^ | VIC:31756 | Viçosa, Minas Gerais, Brazil | 20°44'31.71''S; 42°52'43.83''W | Fungus garden of *Acromyrmex subterraneus molestans* | KF293284 | Augustin et al. (2013) |
| *Escovopsis moelleri* | CBS 135748 ^ET^ | VIC:31753 | Viçosa, Minas Gerais, Brazil | 20°44'31.71''S 42°52'43.83''W | Fungus garden of *Acromyrmex subterraneus molestans* | JQ855715 | Augustin et al. (2013) |
| *Escovopsis multiformis* | LESF 1136 | QVM277 | Alta Floresta, Mato Grosso, Brazil | 09°49'22.7''S 58°15'32.0''W | Fungus garden of *Apterostigma* sp. | MH715106 | Montoya et al. (2019) |
| *Escovopsis multiformis* | CBS 145327 ^ET^ | 1606w | Florianópolis, Santa Catarina, Brazil | 27°28'11.28''S 48°22'39.48''W | Fungus garden of *Apterostigma* sp. | MH715105 | Montoya et al. (2019) |
| *Escovopsis multiformis* | LESF 852 | 1706B | Florianópolis, Santa Catarina, Brazil | 27°44'39.4''S; 48°31'10.0''W | Fungus garden of *Apterostigma* sp. | MT273549 | This study |
| *Escovopsis primorosea* | UNQ E29 | E29 | Tucumán, Argentina | 7°13’51’’S; 65°54’46’’W | Fungus garden of *Acromyrmex aspersus* | KU298290 | Marfetán et al. (2018) |
| *Escovopsis primorosea* | UNQ E30 | E30 | Tucumán, Argentina | 27°13′51′′S; 65°54′47′′W | Fungus garden of *Acromyrmex aspersus* | KU298291 | Marfetán et al. (2018) |
| *Escovopsis primorosea* | UNQ E42 | E42 | Tucumán, Argentina | 27°13′51′′S; 65°54′47′′W | Fungus garden of *Acromyrmex aspersus* | KU298293 | Marfetán et al. (2018) |
| *Escovopsis primorosea* | UNQ E42(2) | E42(2) | Tucumán, Argentina | 27°13′51′′S; 65°54′47′′W | Fungus garden of *Acromyrmex aspersus* | KU298306 | Marfetán et al. (2018) |
| *Escovopsis pseudoweberi* | UNQ E4 | E4 | Buenos Aires, Argentina | 34°50′44′S; 58°07′05′′W | Fungus garden of *Acromyrmex lundii* | KU298300 | Marfetán et al. (2018) |
| *Escovopsis pseudoweberi* | UNQ E10(2) | E10(2) | Corrientes, Argentina | 29°11′50′′S; 58°02′28′′W | Fungus garden of *Acromyrmex lundii* | KU298297 | Marfetán et al. (2018) |
| *Escovopsis pseudoweberi* | UNQ E12 | E12 | Corrientes, Argentina | 29°11′57′′S; 58°02′18′′W | Fungus garden of *Acromyrmex heyeri* | KU298298 | Marfetán et al. (2018) |
| *Escovopsis pseudoweberi* | UNQ E13 | E13 | Corrientes, Argentina | 29°11′50′′S; 58°02′28′′W | Fungus garden of *Acromyrmex lundii* | KU298307 | Marfetán et al. (2018) |
| *Escovopsis pseudoweberi* | UNQ E20 | E20 | Santa Fé, Argentina | 30°22′56′′S; 61°14′16′′W | Fungus garden of *Acromyrmex lobcornis* | KU298299 | Marfetán et al. (2018) |
| *Escovopsis pseudoweberi* | UNQ E24 | E24 | Tucumán, Argentina | 7°13′50′′S; 65°55′51′′W | Fungus garden of *Acromyrmex aspersus* | KU298301 | Marfetán et al. (2018) |
| *Luteomyces trichodermoides* | LESF 310 | AR14022604A1 | Florianópolis, Santa Catarina, Brazil | 27°37'49.62''S, 48°27'3.6''W | Fungus garden of *Mycetophylax morschi* | MH715102 | Montoya et al. (2019) |
| *Luteomyces trichodermoides* | LESF 311 | AR14022604A2 | Florianópolis, Santa Catarina, Brazil | 27°37'49.62''S, 48°27'3.6''W | Fungus garden of *Mycetophylax morschi* | MH715103 | Montoya et al. (2019) |
| *Luteomyces trichodermoides* | LESF 312 | AR14022604ALA | Florianópolis, Santa Catarina, Brazil | 27°37'49.62''S, 48°27'3.6''W | Fungus garden of *Mycetophylax morschi* | MH715104 | Montoya et al. (2019) |
| *Luteomyces trichodermoides* | LESF 832 | 13I3 | Rio Claro, São Paulo, Brazil |  | Fungus garden of *Mycocepurus smithii* | MT273542 | This study |
| *Luteomyces trichodermoides* | LESF 833 | 13I1 | Rio Claro, São Paulo, Brazil |  | Fungus garden of *Mycocepurus smithii* | MT273543 | This study |
| *Luteomyces trichodermoides* | LESF 834 | 13I2 | Rio Claro, São Paulo, Brazil |  | Fungus garden of *Mycocepurus smithii* | MT273544 | This study |
| *Luteomyces trichodermoides* | LESF 835 | 3I1 | Rio Claro, São Paulo, Brazil |  | Midden of *Mycocepurus smithii* | MT273545 | This study |
| *Luteomyces trichodermoides* | LESF 837 | 2I2 | Rio Claro, São Paulo, Brazil |  | Fungus garden of *Mycocepurus smithii* | MT273547 | This study |
| *Luteomyces trichodermoides* | LESF 838 | 2I3 | Rio Claro, São Paulo, Brazil |  | Fungus garden of *Mycocepurus smithii* | MT273548 | This study |
| *Luteomyces trichodermoides* | LESF 895 | Q03I | Botucatu, São Paulo, Brazil | 22°54'19.6''S; 48°14' 33.7''W | Fungus garden of *Mycocepurus goeldii* | MT273559 | This study |
| *Luteomyces trichodermoides* | LESF 897 | Q03III | Botucatu, São Paulo, Brazil | 22°54'19.6''S; 48°14'33.7''W | Fungus garden of *Mycocepurus goeldii* | MT273561 | This study |
| *Luteomyces trichodermoides* | LESF 927 | Q23III | Botucatu, São Paulo, Brazil | 22° 54' 41.7''S; 48°14' 49.5'' NMG | Fungus garden of *Mycocepurus goeldii* | MT273563 | This study |
| *Luteomyces trichodermoides* | LESF 1049 | QVM177 | Botucatu, São Paulo, Brazil | 22°54'20.6''S; 48°14'34.2''W | Fungus garden of *Mycocepurus goeldii* | MT273581 | This study |
| *Luteomyces trichodermoides* | LESF 1051 | QVM179 | Botucatu, São Paulo, Brazil | 22°54'19.8''S; 48°14'33.6''W | Fungus garden of *Mycocepurus goeldii* | MT273582 | This study |
| *Luteomyces trichodermoides* | LESF 1052 | QVM180 | Botucatu, São Paulo, Brazil | 22°54'19.8''S; 48°14'33.6''W | Fungus garden of *Mycocepurus goeldii* | MT273583 | This study |
| *Luteomyces trichodermoides* | LESF 1055 | QVM183 | Botucatu, São Paulo, Brazil | 22°54'19.6''S; 48°14'33.6''W | Fungus garden of *Mycocepurus goeldii* | MT273584 | This study |
| *Luteomyces trichodermoides* | LESF 1057 | QVM185 | Botucatu, São Paulo, Brazil | 22°54'18.8''S; 48°14'33.3''W | Fungus garden of *Mycocepurus goeldii* | MT273585 | This study |
| *Luteomyces trichodermoides* | LESF 1061 | QVM189 | Botucatu, São Paulo, Brazil | 22°54'19.5''S; 48°14'32.7''W | Fungus garden of *Mycocepurus goeldii* | MT273586 | This study |
| *Luteomyces trichodermoides* | LESF 1077 | QVM205 | Botucatu, São Paulo, Brazil | 22°54'19.2''S; 48°14'32.3''W | Fungus garden of *Mycocepurus goeldii* | MT273587 | This study |
| *Luteomyces trichodermoides* | LESF 1078 | QVM206 | Botucatu, São Paulo, Brazil | 22°54'19.2''S; 48°14'32.3''W | Fungus garden of *Mycocepurus goeldii* | MT273588 | This study |
| *Luteomyces trichodermoides* | LESF 1082 | QVM210 | Botucatu, São Paulo, Brazil | 22°54'19.2''S; 48°14'32.3''W | Fungus garden of *Mycocepurus goeldii* | MT273589 | This study |
| *Luteomyces trichodermoides* | LESF 1090 | QVM218 | Botucatu, São Paulo, Brazil | 22°54'16.7''S; 48°14'31.3''W | Fungus garden of *Mycocepurus goeldii* | MT273590 | This study |
| *Luteomyces trichodermoides* | LESF 1109 | QVM237 | Botucatu, São Paulo, Brazil | 22°54'17.9''S; 48°14'33.0''W | Fungus garden of *Mycocepurus goeldii* | MT273591 | This study |
| *Luteomyces trichodermoides* | CBS 137343 ^ET^ | VEM001 | Rio Claro, São paulo, Brazil | 22°23'46.93''S, 47°32'40.12''W | Fungus garden of *Mycocepurus goeldii* | MF116052 | Osti and Rodrigues (2018) |
| *Escovopsis weberi* | UNQ E16 | E16 | Santa Fé, Argentina | 30°22′54′′S; 61°14′15′′W | Fungus garden of *Acromrmex lundii* | KU298308 | Marfetán et al. (2018) |
| *Escovopsis weberi* | UNQ E22 | E22 | La Pampa, Argentina | 36°36′58′′S; 64°18′51′′W | Fungus garden of *Acromyrmex striatus* | KU298304 | Marfetán et al. (2018) |
| *Escovopsis weberi* | UNQ E26 | E26 | La Pampa, Argentina | 36°32′42′′S; 64°03′01′′W | Fungus garden of *Acromyrmex striatus* | KU298305 | Marfetán et al. (2018) |
| *Escovopsis weberi* | UNQ E31 | E31 | Salta, Argentina | 25°13′59′′S; 64°42′48′′W | Fungus garden of *Acromrmex lundii* | KU298292 | Marfetán et al. (2018) |
| *Escovopsis weberi* | UNQ E41 | E41 | Salta, Argentina | 5°13′61′′S; 64°42′53′′W | Fungus garden of *Acromrmex lundii* | KU298294 | Marfetán et al. (2018) |
| *Escovopsis weberi* | ATCC 64542 ^ET^ |  | Viçosa, Minas Gerais, Brazil |  | Carpenter ant fungal mass | KF293281 | Augustin et al. (2013) |
| *Escovopsis weberi* | LESF 046 | SES001 | Rio Claro, São Paulo, Brazil | 22°23'45.9''S; 47°32'43.2''W | Fungus garden of *Trachymyrmex* sp. | MT273511 | This study |
| *Escovopsis weberi* | LESF 355 | ES021 | Corumbataí, São Paulo, Brazil |  | Fungus garden of *Atta sexdens rubropilosa* | MT273534 | This study |
| *Escovopsis weberi* | LESF 017 | NL001 | Botucatu, São Paulo, Brazil | 22°50'46.44''S; 48°26'9.6''W | Midden of *Atta capiguara* | MH715113 | Montoya et al. (2019) |
| *Escovopsis weberi* | LESF 019 | NL005 | Botucatu, São Paulo, Brazil | 22°50'45.8''S; 48°26'09.4''W | Fungus garden of *Atta sexdens rubropilosa* | MH715115 | Montoya et al. (2019) |
| *Escovopsis weberi* | LESF 020 | NL006 | Botucatu, São Paulo, Brazil | 22°50'45.8''S; 48°26'09.4''W | Fungus garden of *Atta sexdens rubropilosa* | MT273503 | This study |
| *Escovopsis weberi* | LESF 023 | ES005 | Alta Floresta, Mato Grosso, Brazil |  | Fungus garden of *Atta cephalotes* | MH715117 | Montoya et al. (2019) |
| *Escovopsis weberi* | LESF 024 | ES006 | Alta Floresta, Mato Grosso, Brazil |  | Fungus garden of *Acromyrmex coronatus* | MT273504 | This study |
| *Escovopsis weberi* | LESF 025 | ES007 | Alta Floresta, Mato Grosso, Brazil |  | Fungus garden of *Acromyrmex coronatus* | MT273505 | This study |
| *Escovopsis weberi* | LESF 027 | ES010 | Rio Claro, São Paulo, Brazil |  | Fungus garden of *Acromyrmex landolti* | MH715119 | Montoya et al. (2019) |
| *Escovopsis weberi* | LESF 029 | ES012 | Corumbataí, São Paulo, Brazil | 22°17'22''S;  47°39'23''W | Fungus garden of *Atta sexdens* | MH715120 | Montoya et al. (2019) |
| *Escovopsis weberi* | LESF 030 | ES013 | Corumbataí, São Paulo, Brazil | 22°17'22''S;  47°39'23''W | Fungus garden of *Atta sexdens* | MH715121 | Montoya et al. (2019) |
| *Escovopsis weberi* | LESF 031 | ES014 | Corumbataí, São Paulo, Brazil | 22°17'22''S;  47°39'23''W | Fungus garden of *Atta sexdens* | MT273506 | This study |
| *Escovopsis weberi* | LESF 033 | ES004 | Bahia, Brazil |  | Fungus garden of *Acromyrmex* sp. | MT273507 | This study |
| *Escovopsis weberi* | LESF 034 | ES024 | Botucatu, São Paulo, Brazil |  | Fungus garden of *Acromyrmex balzanii* | MT273508 | This study |
| *Escovopsis weberi* | LESF 042 | RS053 | Chuvisca, Rio Grande do Sul, Brazil | 30°50'10.2"S; 51°55'10.4"W | Fungus garden of *Acromyrmex lundii* | MT273509 | This study |
| *Escovopsis weberi* | LESF 043 | RS055 | Chuvisca, Rio Grande do Sul, Brazil | 30°50'10.2"S; 51°55'10.4"W | Fungus garden of *Acromyrmex heyeri* | MT273510 | This study |
| *Escovopsis weberi* | LESF 054 | AR003 | Ilhéus, Bahia, Brazil | 14°47'56.8''S; 39°10'16.4''W | Fungus garden of *Acromyrmex* *balzanii* | MT273512 | This study |
| *Escovopsis weberi* | LESF 056 | AR033 | Camacan, Bahia, Brazil | 15°22'50.3''S; 39°34'03.5''W | Fungus garden of *Acromyrmex* sp. | MT273513 | This study |
| *Escovopsis weberi* | LESF 136 | 4a | Corumbataí, São Paulo, Brazil | 22°17'21.7''S; 47°39'22.8''W | Fungus garden of *Atta sexdens rubropilosa* | MT273514 | This study |
| *Escovopsis weberi* | LESF 146 | 1cT4 | Corumbataí, São Paulo, Brazil | 22°17'21.7''S; 47°39'22.8''W | Fungus garden of *Atta sexdens rubropilosa* | MT273515 | This study |
| *Escovopsis weberi* | LESF 156 | A088 | Corumbataí, São Paulo, Brazil | 22°17'21.7''S; 47°39'22.8''W | Fungus garden of *Atta sexdens rubropilosa* | MT273516 | This study |
| *Escovopsis weberi* | LESF 178 | A086a | Corumbataí, São Paulo, Brazil | 22°17'21.7''S; 47°39'22.8''W | Fungus garden of *Atta sexdens rubropilosa* | MT273517 | This study |
| *Escovopsis weberi* | LESF 239 | 13B | Corumbataí, São Paulo, Brazil | 22°17'21.7''S; 47°39'22.8''W | Fungus garden of *Atta sexdens rubropilosa* | MT273518 | This study |
| *Escovopsis weberi* | LESF 241 | H1b | Corumbataí, São Paulo, Brazil | 22°17'21.7''S; 47°39'22.8''W | Fungus garden of *Atta sexdens rubropilosa* | MT273519 | This study |
| *Escovopsis weberi* | LESF 292 | NL003 | Botucatu, São Paulo, Brazil | 22°50'46.4"S 48°26'09.6"W | Fungus garden of *Atta capiguara* | MT273520 | This study |
| *Escovopsis weberi* | LESF 294 | H33 | Corumbataí, São Paulo, Brazil | 22°17'21.7''S; 47°39'22.8''W | Fungus garden of *Atta sexdens rubropilosa* | MT273521 | This study |
| *Escovopsis weberi* | LESF 295 | NL009 | Botucatu, São Paulo, Brazil | 22°50'45.8''S; 48°26'09.4''W | Fungus garden of *Atta sexdens rubropilosa* | MT273522 | This study |
| *Escovopsis weberi* | LESF 298 | NL004 | Botucatu, São Paulo, Brazil | 22°50'46.4"S 48°26'09.6"W | Fungus garden of *Atta capiguara* | MT273523 | This study |
| *Escovopsis weberi* | LESF 315 | NL007 | Botucatu, São Paulo, Brazil | 22°50'45.8''S; 48°26'09.4''W | Fungus garden of *Atta sexdens rubropilosa* | MH715125 | Montoya et al. (2019) |
| *Escovopsis weberi* | LESF 317 | ES026 | Rio Claro, São Paulo, Brazil |  | Fungus garden of *Trachymyrmex* sp. | MT273531 | This study |
| *Escovopsis weberi* | LESF 319 | ES030 | Palmas, Tocantins, Brazil | 10°10'52.9"S; 48°21'42.0"W | Fungus garden of *Acromyrmex* sp. | MT273532 | This study |
| *Escovopsis weberi* | LESF 324 | RS105 | Thermas de Santa Bárbara, São Paulo, Brazil | 22º49'10.6"S; 49º16'06.2"W | Fungus garden of *Atta laevigata* | MT273533 | This study |
| *Escovopsis weberi* | LESF 356 | ES032 | Botucatu, São Paulo, Brazil |  | Fungus garden of *Atta laevigata* | MT273535 | This study |
| *Escovopsis weberi* | LESF 359 | ES019 | Corumbataí, São Paulo, Brazil |  | Fungus garden of *Atta sexdens* | MT273536 | This study |
| *Escovopsis weberi* | LESF 362 | ES028 | Corumbataí, São Paulo, Brazil |  | Fungus garden of *Atta sexdens* | MT273537 | This study |
| *Escovopsis weberi* | LESF 363 | ES023 | Corumbataí, São Paulo, Brazil |  | Fungus garden of *Atta sexdens* | MT273538 | This study |
| *Escovopsis weberi* | LESF 364 | ES015 | Corumbataí, São Paulo, Brazil |  | Fungus garden of *Atta sexdens* | MT273539 | This study |
| *Escovopsis weberi* | LESF 519 | ES016 |  |  | Fungus garden of *Atta sexdens rubropilosa* | MT273540 | This study |
| *Escovopsis weberi* | LESF 575 | RS087 | Indaial, Santa Catarina, Brazil | 26º54'04.9"S; 49º10'51.2"W | Fungus garden of *Acromyrmex diciger* | MT273541 | This study |
| *Escovopsis weberi* | LESF 858 | A210201 | Camacan, Bahia, Brazil |  | Fungus garden of *Atta cephalotes* | MT273550 | This study |
| *Escovopsis weberi* | LESF 859 | B110302 | Camacan, Bahia, Brazil |  | Fungus garden of *Atta cephalotes* | MT273551 | This study |
| *Escovopsis weberi* | LESF 877 | NL010 |  |  |  | MT273555 | This study |
| *Escovopsis weberi* | LESF 880 | 2aT=3 |  |  |  | MT273556 | This study |
| *Escovopsis weberi* | LESF 994 | QVM81 | Novo Airão, Amazonas, Brazil | 2°36'37.9''S; 60°52'34.4''W | Fungus garden of *Acromyrmex* sp. | MT273568 | This study |
| *Escovopsis* sp*.* | LESF 052 | SES010 | Manaus, Amazonas, Brazil |  | Fungus garden of *Trachymyrmex diversus* | MH715124 | Montoya et al. (2019) |
| *Escovopsis* sp*.* | LESF 325 | BA004 | Camacan, Bahia, Brazil | 14°47'56.8''S; 39°10'16.4''W | Fungus garden of *Atta cephalotes* | MH715127 | Montoya et al. (2019) |
| *Escovopsis* sp. | LESF 962 | QVM49 | Novo Airão, Amazonas, Brazil | 2°16'15.7''S; 61°01'8.5''W | Fungus garden of *Acromyrmex* sp. | MT273564 | This study |
| *Escovopsis* sp*.* | LESF 969 | QVM56 | Novo Airão, Amazonas, Brazil | 2°31'23.4''S; 60°49'31.9''W | Fungus garden of *Apterostigma* sp. | MT273565 | This study |
| *Escovopsis* sp. | LESF 975 | QVM62 | Novo Airão, Amazonas, Brazil | 2°31'25.3''S; 60°49'33.1''W | Fungus garden of *Trachymyrmex* sp. | MT273566 | This study |
| *Escovopsis* sp. | LESF 979 | QVM66 | Novo Airão, Amazonas, Brazil |  | Fungus garden of *Trachymyrmex* sp | MT273567 | This study |
| *Escovopsis* sp. | LESF 996 | QVM83 | Novo Airão, Amazonas, Brazil | 2°32'02.7''S; 60°50'11.7''W | Fungus garden of *Apterostigma* sp. | MT273569 | This study |
| *Escovopsis* sp. | LESF 997 | QVM84 | Novo Airão, Amazonas, Brazil | 2°31'23.4''S; 60°49'31.9''W | Fungus garden of *Trachymyrmex* sp | MT273570 | This study |
| *Escovopsis* sp. | LESF 1003 | QVM90 | Novo Airão, Amazonas, Brazil | 2°32'1.4''S;  60°50'0.4''W | Fungus garden of *Trachymyrmex* sp. | MT273571 | This study |
| *Sympodiorosea* sp. | LESF 864 | SES030331-05 |  |  |  | MT273553 | This study |
| *Sympodiorosea* sp. | LESF 886 | UGM23 |  |  |  | MT273557 | This study |
| *Sympodiorosea* sp. | LESF 887 | UGM26(C) |  |  |  | MT273558 | This study |
| *Sympodiorosea* sp. | LESF 899 | Q03V | Botucatu, São Paulo, Brazil | 22°54'19.6''S; 48°14'33.7''W | Fungus garden of *Mycocepurus goeldii* | MT273562 | This study |
| *Sympodiorosea* sp. | LESF 1010 | QVM138 | Manaus, Amazonas, Brazil | 2°26'51.6''S; 59°45'53.4''W | Fungus garden of *Apterostigma* sp. | MT273573 | This study |
| *Cladobotryum asterophorum* | CBS 676.77 |  | Japan |  |  | AJ583469 | Põldmaa (2011) |
| *Cladobotryum protrusum* | TFC 201316 |  | Madagascar |  | Eucalyptus forest | FN859414 | Põldmaa (2011) |
| *Hypomyces samuelsii* | TFC 2007-23 |  | Peru |  | on  basidioma of an agaricoid basidiomycete on a stem of a palm | FN859451 | Põldmaa (2011) |
| *Hypomyces semicirculare* | CBS 705. 88 |  | Cuba |  | On old  polypore | FN859417 | Põldmaa (2011) |
| *Lecanicillium antillanum* | CBS 350.85 |  | Cuba |  | on  basidioma of an agaricoid | AF339536 | Spatafora et al. (2007) |
| *Protocrea pallida* | TFC 99-209 |  | New York, Cleaveland |  |  | EU710769 | Jaklitsch et al. (2011) |
| *Sphaerostilbella aureonitens* | GJS 74-87 |  |  |  |  | HM466683 | unpublished |
| *Trichoderma harzianum* | CBS 226.95 |  | England |  |  | HM466680 | Chaverri et al. (2003) |

Table S5. Strains of the *Hypocreales* and their metadata used in the phylogenetic analysis at order-level (Fig. 2).

| **Taxa** | **Specimen voucher** | **Host/substratum** | **GenBank accessions** | | | |
| --- | --- | --- | --- | --- | --- | --- |
|  |  |  | **LSU** | ***tef*1** | ***rpb*1** | ***rpb*2** |
| *Aphysiostroma stercorarium* | ATCC 62321 | on cow dung | AF543792 | AF543782 | AY489633 | EF469103 |
| *Aschersonia badia* | BCC 8105 | scale insect (Hemiptera) | DQ518752 | DQ522317 | DQ522363 | DQ522411 |
| *Aschersonia placenta* | BCC 7869 | scale insect (Hemiptera) | EF469074 | EF469056 | EF469085 | EF469104 |
| *Balansia henningsiana* | GAM 16112 | *Panicum* sp. (Poaceae) | AY545727 | AY489610 | AY489643 | DQ522413 |
| *Balansia pilulaeformis* | AEG 94-2 | Poaceae | AF543788 | DQ522319 | DQ522365 | DQ522414 |
| *Claviceps paspali* | ATCC 13892 | Poaceae | U47826 | DQ522321 | DQ522367 | DQ522416 |
| *Claviceps purpurea* | GAM 12885 | *Dactylis glomerata* (Poaceae) | AF543789 | AF543778 | AY489648 | DQ522417 |
| *Cordyceps bifusispora* | EFCC 5690 | lepidopteran pupa | EF468806 | EF468746 | EF468854 | EF468909 |
| *Cordyceps cardinalis* | OSC 93609 | lepidopteran larva | AY184962 | DQ522325 | DQ522370 | DQ522422 |
| *Cordyceps kyusyuënsis* | EFCC 5886 | lepidopteran pupa | EF468813 | EF468754 | EF468863 | EF468917 |
| *Cordyceps militaris* | OSC 93623 | lepidopteran pupa | AY184966 | DQ522332 | DQ522377 | AY545732 |
| *Cordyceps* cf. *ochraceostromata* | ARSEF 5691 | Lepidoptera | EF468819 | EF468759 | EF468867 | EF468921 |
| *Cordyceps scarabaeicola* | ARSEF 5689 | scarabaeid adult (Coleoptera) | AF339524 | DQ522335 | DQ522380 | DQ522431 |
| *Cordyceps* cf. *takaomontana* | NHJ 12623 | Lepidoptera | EF468838 | EF468778 | EF468884 | EF468932 |
| *Cordyceps tuberculata* | OSC 111002 | Lepidoptera | DQ518767 | DQ522338 | DQ522384 | DQ522435 |
| *Cosmospora cocinea* | CBS 114050  AR2741 | *Inonotus nodulosus* (Hymenomycetes) | AY489734 | AY489629 | AY489667 | DQ522438 |
| *Elaphocordyceps capitata* | OSC 71233 | *Elaphomyces* sp. (Euascomycetes) | AY489721 | AY489615 | AY489649 | DQ522421 |
| *Elaphocordyceps fracta* | OSC 110990 | *Elaphomyces* sp. (Euascomycetes) | DQ518759 | DQ522328 | DQ522373 | DQ522425 |
| *Elaphocordyceps japonica* | OSC 110991 | *Elaphomyces* sp. (Euascomycetes) | DQ518761 | DQ522330 | DQ522375 | DQ522428 |
| *Elaphocordyceps longisegmentis* | OSC 110992 | *Elaphomyces* sp. (Euascomycetes) | EF468816 | --- | EF468864 | EF468919 |
| *Elaphocordyceps ophioglossoides* | OSC 106405 | *Elaphomyces* sp. (Euascomycetes) | AY489723 | AY489618 | AY489652 | DQ522429 |
| *Elaphocordyceps subsessilis* | OSC 71235 | scarabaeid larva (Coleoptera) | EF469077 | EF469061 | EF469090 | EF469108 |
| *Engyodontium aranearum* | CBS 309.85 | spider (Arachnida) | AF339526 | DQ522341 | DQ522387 | DQ522439 |
| *Epichloë typhina* | ATCC 56429 | *Festuca rubra* (Poaceae) | U17396 | AF543777 | AY489653 | DQ522440 |
| *Haptocillium balanoides* | CBS 250.82 | nematode | AF339539 | DQ522342 | DQ522388 | DQ522442 |
| *Haptocillium sinense* | CBS 567.95 | nematode | AF339545 | DQ522343 | DQ522389 | DQ522443 |
| *Haptocillium zeosporum* | CBS 335.80 | nematode | AF339540 | EF469062 | EF469091 | EF469109 |
| *Hirsutella* sp. | OSC 128575 | hemipteran adult | EF469079 | EF469064 | EF469093 | EF469110 |
| *Hydropisphaera erubescens* | ATCC 36093 | *Cordyline banksii* (Laxmanniaceae) | AY545726 | DQ522344 | DQ522390 | AY545731 |
| *Hydropisphaera peziza* | CBS 102038  GJS92-101 | on bark | AY489730 | AY489625 | AY489661 | DQ522444 |
| *Hypocrea lutea* | ATCC 208838 | on decorticated conifer wood | AF543791 | AF543781 | AY489662 | DQ522446 |
| *Hypocrea rufa* | GJS 89-127 | on bark | AY489726 | AY489621 | AY489656 | AF545521 |
| *Hypocrella schizostachyi* | BCC 14123 | scale insect (Hemiptera) | DQ518771 | DQ522346 | DQ522392 | DQ522447 |
| *Hypocrella nectrioides* | GJS 89-104 | scale insect (Hemiptera) | U47832 | DQ522347 | DQ522393 | DQ522448 |
| *Escovopsis weberi* | ATCC 64542 ^ET^ | Carpenter ant fungal mass | KF293281 | MZ170961 | MT305412 | MT305537 |
| *Escovopsis* *aspergilloides* | CBS 423.93 ^ET^ | Fungus garden of *Trachymyrmex ruthae* | KF293283 | AY172632 | MT305421 | MT305546 |
| *Escovopsis clavata* | CBS 145326 ^ET^ | Fungus garden of *Apterostigma* sp. | MH715110 | MH724270 | MT305419 | MT305544 |
| *Escovopsis lentecrescens* | CBS 135750 ^ET^ | Fungus garden of *Acromyrmex subterraneus subterraneus* | JQ855717 | JQ855714 | MT305415 | MT305540 |
| *Escovopsis microspora* | CBS 135751^ET^ | Fungus garden of *Acromyrmex subterraneus molestans* | KF293284 | KJ935030 | MT305416 | MT305541 |
| *Escovopsis moelleri* | CBS 135748 ^ET^ | Fungus garden of *Acromyrmex subterraneus molestans* | JQ855715 | JQ855712 | MT305413^#^ | MT305538^#^ |
| *Escovopsis multiformis* | CBS 145327 ^ET^ | Fungus garden of *Apterostigma* sp. | MH715105 | MH724265 | MT305420^#^ | MT305545^#^ |
| *Luteomyces trichodermoides* | CBS 137343 ^ET^ | Fungus garden of *Mycocepurus goeldii* | MF116052 | KF033128 | MT305417 | MT305542 |
| *Luteomyces trichodermoides* | LESF 310 | Fungus garden of *Mycetophylax morschi* | MH715102 | MH724262 | MT305460 | MT305585 |
| *Luteomyces trichodermoides* | LESF 311 | Fungus garden of *Mycetophylax morschi* | MH715103 | MH724263 | MT305461 | MT305586 |
| *Luteomyces trichodermoides* | LESF 312 | Fungus garden of *Mycetophylax morschi* | MH715104 | MH724264 | MT305462 | MT305587 |
| *Luteomyces trichodermoides* | LESF 832 | Fungus garden of *Mycocepurus smithii* | MT273542 | MT305366 | MT305488 | MT305613 |
| *Luteomyces trichodermoides* | LESF 833 | Fungus garden of *Mycocepurus smithii* | MT273543 | MT305367 | MT305489 | MT305614 |
| *Luteomyces trichodermoides* | LESF 834 | Fungus garden of *Mycocepurus smithii* | MT273544 | MT305368 | MT305490 | MT305615 |
| *Luteomyces trichodermoides* | LESF 835 | Midden of *Mycocepurus smithii* | MT273545 | MT305369 | MT305491 | MT305616 |
| *Sympodiorosea kreiselii* | LESF 302 | Fungus garden of *Mycetophylax morschi* | MH715099 | MH724259 | MT305452 | MT305577 |
| *Sympodiorosea kreiselii* | LESF 303 | Fungus garden of *Mycetophylax morschi* | MH715100 | MH724260 | MT305453 | MT305578 |
| *Sympodiorosea kreiselii* | LESF 304 | Fungus garden of *Mycetophylax morschi* | MH715101 | MH724261 | MT305454 | MT305579 |
| *Sympodiorosea kreiselii* | LESF 305 | Fungus garden of *Mycetophylax morschi* | MT273524 | MT305353 | MT305455 | MT305580 |
| *Sympodiorosea kreiselii* | LESF 306 | Fungus garden of *Mycetophylax morschi* | MT273525 | MT305354 | MT305456 | MT305581 |
| *Sympodiorosea kreiselii* | LESF 307 | Fungus garden of *Mycetophylax morschi* | MT273526 | MT305355 | MT305457 | MT305582 |
| *Sympodiorosea kreiselii* | LESF 308 | Fungus garden of *Mycetophylax morschi* | MT273527 | MT305356 | MT305458 | MT305583 |
| *Sympodiorosea kreiselii* | LESF 309 | Fungus garden of *Mycetophylax morschi* | MT273528 | MT305357 | MT305459 | MT305584 |
| *Sympodiorosea kreiselii* | CBS 139320 ^ET^ | Fungus garden of *Mycetophylax morschi* | KJ808765 | KJ808766 | MT305418 | MT305543 |
| *Sympodiorosea* sp. | LESF 864 |  | MT273553 | MT305375 | MT305499 | MT305624 |
| *Sympodiorosea* sp. | LESF 886 |  | MT273557 | MT305378 | MT305502 | MT305627 |
| *Sympodiorosea* sp. | LESF 887 |  | MT273558 | MT305379 | MT305503 | MT305628 |
| *Sympodiorosea* sp. | LESF 899 | Fungus garden of *Mycocepurus goeldii* | MT273562 | MT305382 | MT305506 | MT305631 |
| *Sympodiorosea* sp. | LESF 1010 | Fungus garden of *Apterostigma* sp. | MT273573 | MT305393 | MT305517 | MT305642 |
| *Escovopsioides nivea* | CBS 135749^ET^ | Fungus garden of *Acromyrmex subterraneus subterraneus* | JQ855716 | JQ855713 | MT305414 | MT305539 |
| *Escovopsioides nivea* | LESF 588 | Fungus garden of *Atta cephalotes* | MF116037 | MF140952 | MT305478 | MT305603 |
| *Escovopsioides nivea* | LESF 596 | Fungus garden of *Acromyrmex* sp. | MF116045 | MF140960 | MT305483 | MT305608 |
| *Escovopsioides nivea* | LESF 599 | Fungus garden of *Acromyrmex heyeri* | MF116048 | MF140963 | MT305486 | MT305611 |
| *Escovopsioides nivea* | LESF 601 | Fungus garden of *Trachymyrmex* sp. | MF116049 | MF140964 | MT305487 | MT305612 |
| *Escovopsioides nivea* | LESF 587 | Fungus garden of *Atta cephalotes* | MF116036 | MF140951 | MT305477 | MT305602 |
| *Escovopsioides nivea* | LESF 589 | Fungus garden of *Atta cephalotes* | MF116038 | MF140953 | MT305479 | MT305604 |
| *Escovopsioides nivea* | LESF 1009 | Fungus garden of *Apterostigma* sp. | MT273572 | MT305392 | MT305516 | MT305641 |
| *Cladobotryum asterophorum* | CBS 676.77 | Japan | AJ583469 | FN868712 | FN868776 | FN868649 |
| *Cladobotryum protrusum* | TFC 201316 | Madagascar | FN859414 | FN868732 | FN868795 | FN868668 |
| *Hypomyces samuelsii* | TFC 2007-23 | Peru | FN859451 | FN868769 | FN868828 | FN868705 |
| *Hypomyces semicirculare* | CBS 705. 88 | Cuba | FN859417 | FN868735 | FN868671 | FN868798 |
| *Protocrea pallida* | TFC 99-209 | New York, Cleaveland | EU710769 | EU703903 | --- | EU703949 |
| *Trichoderma harzianum* | CBS 226.95 | England | HM466680 | AF534621 | JQ031082 | AF545549 |
| *Hypomyces polyporinus* | ATCC 76479 | *Trametes versicolor* (Hymenomycetes) | AF543793 | AF543784 | AY489663 | --- |
| *Isaria* cf. *farinosa* | OSC 111004 | lepidopteran pupa | EF468840 | EF468780 | EF468886 | --- |
| *Isaria tenuipes* | OSC 111007 | lepidopteran pupa | DQ518773 | DQ522349 | DQ522395 | DQ522449 |
| *Lecanicillium antillanum* | CBS 350.85 | agaric (Hymenomycetes) | AF339536 | DQ522350 | DQ522396 | DQ522450 |
| *Lecanicillium aranearum* | CBS 726.73a | spider (Arachnida) | AF339537 | EF468781 | EF468887 | EF468934 |
| *Lecanicillium attenuatum* | CBS 402.78 | leaf litter of *Acer saccharum* | AF339565 | EF468782 | EF468888 | EF468935 |
| *Lecanicillium psalliotae* | CBS 532.81 | soil | AF339560 | EF469067 | EF469096 | EF469112 |
| *Lecanicillium psalliotae* | CBS 101270 | soil | EF469081 | EF469066 | EF469095 | EF469113 |
| *Leuconectria clusiae* | ATCC 22228 | soil | AY489732 | AY489627 | AY489664 | EF469114 |
| *Mariannaea pruinosa* | ARSEF 5413 | *Iragoides fasciata* (Lepidoptera) | AY184968 | DQ522351 | DQ522397 | DQ522451 |
| *Melanopsamma pomifromis* | ATCC 18873 | *Ulmus* sp. (Ulmaceae) | AY489709 | AY489604 | AY489637 | EF692511 |
| *Metacordyceps chlamydosporia* | CBS 101244 | egg of slug (Diplopoda) | DQ518758 | DQ522327 | DQ522372 | DQ522424 |
| *Metacordyceps taii* | ARSEF 5714 | Lepidoptera | AF543787 | AF543775 | DQ522383 | DQ522434 |
| *Metacordyceps yongmunensis* | EFCC 2131 | lepidopteran pupa | EF468833 | EF468770 | EF468876 | KJ398690 |
| *Metacordyceps* sp. | OSC 110996 | Lepidoptera | EF468832 | EF468773 | EF468880 | EF468928 |
| *Metacordyceps* sp. | NHJ 12118 | Lepidoptera | EF468829 | EF468768 | EF468878 | EF468927 |
| *Metarhizium album* | ARSEF 2082 | *Cofana spectra* (Hemiptera) | DQ518775 | DQ522352 | DQ522398 | DQ522452 |
| *Metarhizium anisopliae* | ARSEF 3145 | *Oryctes rhinoceros* (Coleoptera) | AF339530 | AF543774 | DQ522399 | DQ522453 |
| *Metarhizium flavoviride* | ARSEF 2037 | *Nilaparvata lugens* (Hemiptera) | AF339531 | DQ522353 | DQ522400 | DQ522454 |
| *Microhilum oncoperae* | ARSEF 4358 | *Oncopera intricata* (Lepidoptera) | AF339532 | EF468785 | EF468891 | EF468936 |
| *Myriogenospora atramentosa* | AEG 96-32 | *Andropogon virginicus* (Poaceae) | AY489733 | AY489628 | AY489665 | DQ522455 |
| *Myrothecium cinctum* | ATCC 22270 | soil | AY489710 | AY489605 | AY489638 | EF692512 c |
| *Myrothecium roridum* | ATCC 16297 | soil | AY489708 | AY489603 | AY489636 | EF692513 c |
| *Myrothecium verrucaria* | ATCC 9095 | baled cotton | AY489713 | AY489608 | AY489641 | EF692514 c |
| *Nectria cinnabarina* | CBS 114055 | *Betula* sp. (Betulaceae) | U00748 | AF543785 | AY489666 | DQ522456 |
| *Nomuraea rileyi* | CBS 806.71 | Lepidoptera | AY624250 | EF468787 | EF468893 | EF468937 |
| *Ochronectria calami* | CBS 125.87 | on palm | AY489717 | AY489612 | AY489644 | EF692515 c |
| *Ophiocordyceps* cf*. acicularis* | OSC 128580 | Coleoptera | DQ518757 | DQ522326 | DQ522371 | DQ522423 |
| *Ophiocordyceps agriotidis* | ARSEF 5692 | Coleoptera | DQ518754 | DQ522322 | DQ522368 | DQ522418 |
| *Ophiocordyceps brunneipunctata* | OSC 128576 | Coleoptera | DQ518756 | DQ522324 | DQ522369 | DQ522420 |
| *Ophiocordyceps entomorrhiza* | KEW 53484 | coleopteran larva | EF468809 | EF468749 | EF468857 | EF468911 |
| *Ophiocordyceps gracilis* | EFCC 3101 | lepidopteran larva | EF468810 | EF468750 | EF468858 | EF468913 |
| *Ophiocordyceps nigrella* | EFCC 9247 | lepidopteran larva | EF468818 | EF468758 | EF468866 | EF468920 |
| *Ophiocordyceps ravenelii* | OSC 110995 | coleopteran larva | DQ518764 | DQ522334 | DQ522379 | DQ522430 |
| *Ophiocordyceps rhizoidea* | NHJ 12522 | Isoptera | EF468825 | EF468764 | EF468873 | EF468923 |
| *Ophiocordyceps sinensis* | EFCC 7287 | lepidopteran pupa | EF468827 | EF468767 | EF468874 | EF468924 |
| *Ophiocordyceps stylophora* | OSC 111000 | elaterid larva (Coleoptera) | DQ518766 | DQ522337 | DQ522382 | DQ522433 |
| *Ophiocordyceps unilateralis* | OSC 128574 | ant (Hymenoptera) | DQ518768 | DQ522339 | DQ522385 | DQ522436 |
| *Ophiocordyceps variabilis* | ARSEF 5365 | dipteran larva | DQ518769 | DQ522340 | DQ522386 | DQ522437 |
| *Ophionectria trichospora* | CBS 109876 | on liana | AF543790 | AF543779 | AY489669 | DQ522457 |
| *Paecilomyces carneus* | CBS 239.32 | sand dune | EF468843 | EF468789 | EF468894 | EF468938 |
| *Paecilomyces lilacinus* | CBS 431.87 | *Meloidogyne* sp. (Nematoda) | EF468844 | EF468791 | EF468897 | EF468940 |
| *Paecilomyces marquandii* | CBS 182.27 | soil | EF468845 | EF468793 | EF468899 | EF468942 |
| *Peethambara spirostriata* | CBS 110115 | on leaves of *Buxus sempervirens* | AY489724 | AY489619 | AY489654 | EF692516 ^c^ |
| *Pochonia bulbillosa* | CBS 145.70 | root of *Picea abies* | AF339542 | EF468796 | EF468902 | EF468943 |
| *Pochonia chlamydosporia* | CBS 504.66 | nematode | AF339544 | EF469069 | EF469098 | EF469120 |
| *Pochonia gonioides* | CBS 891.72 | nematode | AF339550 | DQ522354 | DQ522401 | DQ522458 |
| *Pochonia rubescens* | CBS 464.88 | *Heterodera avenae* (Nematoda) | AF339566 | EF468797 | EF468903 | EF468944 |
| *Pseudonectria rousseliana* | CBS 114049 | *Buxus sempervirens* (Buxaceae) | U17416 | AF543780 | AY489670 | DQ522459 |
| *Rotiferophthora angustispora* | CBS 101437 | rotifer (Rotifera) | AF339535 | AF543776 | DQ522402 | DQ522460 |
| *Roumegueriella rufula* | CBS 346.85 | *Globodera rostochiensis* (Nematoda) | DQ518776 | DQ522355 | DQ522403 | DQ522461 |
| *Shimizuomyces paradoxus* | EFCC 6279 | *Smilax sieboldi* (Smilacaceae) | EF469084 | EF469071 | EF469100 | EF469117 |
| *Simplicillium lamellicola* | CBS 116.25 | *Agaricus bisporus* (Hymenomycetes) | AF339552 | DQ522356 | DQ522404 | DQ522462 |
| *Simplicillium lanosoniveum* | CBS 101267 | *Hemileia vastatrix* (Urediales) | AF339554 | DQ522357 | DQ522405 | DQ522463 |
| *Sphaerostilbella aureonitens* | GJS 74-87 | polypore (fungus) | HM466683 | FJ467644 | --- | FJ442763 |
| *Sphaerostilbella berkeleyana* | CBS 102308 | polypore (Hymenomycetes) | U00756 | AF543783 | AY489671 | DQ522465 |
| *Stachybotrys echinata* | UAMH6594 | indoor air | AY489736 | AY489631 | AY489672 | EF692518 ^c^ |
| *Stachybotrys chlorohalonata* | ATCC 66238  UAMH6417  CBS 25089 | Namibia, isol. from  desert sand | AY489712 | AY489607 | AY489640 | EF692517 |
| *Stachybotrys subsimplex* | ATCC 32888 | water hyacinth | AY489711 | AY489606 | AY489639 | EF692519 ^c^ |
| *Stilbocrea macrostoma* | CBS 114375  GJS 73-26 | *Geniostoma ligustifolia* | AY489725 | AY489620 | AY489655 | EF692520 ^c^ |
| *Tolypocladium parasiticum* | ARSEF 3436 | bdelloid rotifer (Rotifera) | EF468848 | EF468799 | EF468904 | EF468945 |
| *Torrubiella confragosa* | CBS 101247 | *Coccus viridis* (Hemiptera) | AF339555 | DQ522359 | DQ522407 | DQ522466 |
| *Torrubiella ratticaudata* | ARSEF 1915 | spider (Arachnida) | DQ518777 | DQ522360 | DQ522408 | DQ522467 |
| *Torrubiella wallacei* | CBS 101237 | Lepidoptera | AY184967 | EF469073 | EF469102 | EF469119 |
| *Verticillium epiphytum* | CBS 384.81 | *Hemileia vastatrix* (Uredinales) | AF339547 | DQ522361 | DQ522409 | DQ522469 |
| *Verticillium incurvum* | CBS 460.88 | *Ganoderma lipsiense* (Hymenomycetes) | AF339551 | DQ522362 | DQ522410 | DQ522470 |
| *Verticillium* sp. | CBS 101284 | spider (Arachnida) | AF339564 | EF468803 | EF468907 | EF468948 |
| *Viridispora diparietispora* | ATCCMYA627 | *Crataegus crus-galli* (Rosaceae) | AY489735 | AY489630 | AY489668 | DQ522471 |

**References**

Augustin JO, Groenewald JZ, Nascimento RJ, Mizubuti ESG, Barreto RW, Elliot SL, Evans HC (2013). Yet more “weeds” in the garden: Fungal novelties from nests of leaf-cutting ants. PLoS One 8:e82265. doi: 10.1371/journal.pone.0082265

Chaverri P, Castlebury LA, Samuels GJ, Geiser DM (2003). Multilocus phylogenetic structure within the *Trichoderma harzianum* / *Hypocrea lixii* complex. Mol Phylogenet Evol 27:302–313. doi: 10.1016/S1055-7903(02)00400-1

Currie CR, Wong B, Stuart AE, Schultz TR, Rehner SA, Mueller UG, Sung GH, Spatafora JW, Straus NA (2003). Ancient tripartite coevolution in the attine ant-microbe symbiosis. Science 299:386–388. doi:10.1126/science.1078155

Gerardo NM, Mueller UG, Price SL, Currie CR (2004) Exploiting a mutualism: parasite specialization on cultivars within the fungus–growing ant symbiosis. Proc Roy Soc B-Biol Sci 271:1791–1798. doi.org/10.1098/rspb.2004.2792

Gerardo NM, Mueller UG, Currie CR (2006) Complex host-pathogen coevolution in the *Apterostigma* fungus-growing ant-microbe symbiosis. BMC Evol Biol 6:88. doi: 10.1186/1471-2148-6-88

Haugland RL, Heckman JL (1998). Identification of putative sequence specific PCR primers for detection of the toxigenic fungal species *Stachybotrys chartarum*. Mol Cell Probes 12:387–396. doi: 10.1006/mcpr.1998.0197

Jaklitsch WM, Põldmaa K, Samuels GJ (2008a) Reconsideration of *Protocrea* (*Hypocreales*, *Hypocreaceae*). Mycologia 100:962-984.

Jaklitsch WM, Kubicek CP, Druzhinina IS (2008b) Three European species of Hypocrea with reddish brown stromata and green ascospores. Mycologia 100: 796-815. doi: 10.3852/08-039

Jaklitsch WM (2011) European species of *Hypocrea* part II: species with hyaline ascospores. Fungal Divers 48:1-250.

Jaklitsch WM, Stadler M, Voglmayr H (2012) Blue pigment in *Hypocrea caerulescens* sp. nov. and two additional new species in sect. *Trichoderma*. Mycologia 104:925-941.

Liu YJ, Whelen S, Hall BD (1999). Phylogenetic relationships among *Ascomycetes*: Evidence from an RNA polymerse II dubunit. Mol Biol Evol 16:1799–1808.

Marfetán JA, Romero AI, Cafaro MJ, Folgarait PJ (2018) Five new *Escovopsis* species from Argentina. Mycotaxon 133:569–589. doi: 10.5248/133.569

Masiulionis VE, Cabello MN, Seifert KA, Rodrigues A, Pagnocca FC (2015). *Escovopsis trichodermoides* sp. nov., isolated from a fungus garden of the lower attine ant *Mycocepurus goeldii*. Antonie van Leeuwenhoek 107: 31–40. doi: 10.1007/s10482-014-0367-1

Meirelles LA, Montoya QV, Solomon SE, Rodrigues A (2015a). New light on the systematics of fungi associated with attine ant gardens and the description of *Escovopsis kreiselii* sp. nov. PLoS One 10:e0112067. doi: 10.1371/journal.pone.0112067

Meirelles LA, Solomon SE, Bacci M, Wright AM, Mueller UG, Rodrigues A (2015b). Shared *Escovopsis* parasites between leaf-cutting and non-leaf-cutting ants in the higher attine fungus-growing ant symbiosis. Roy Soc Open Sci 2:150257. doi:10.1098/rsos.150257

Montoya QV, Martiarena MJS, Polezel DA, Kakazu, S, Rodrigues A (2019). More pieces to a huge puzzle: Two new Escovopsis species from fungus gardens of attine ants. MycoKeys 46:97–118. doi: 10.3897/mycokeys.46.30951

Osti JF, Rodrigues A (2018). *Escovopsioides* as a fungal antagonist of the fungus cultivated by leafcutter ants. BMC Microbiol 18:130. doi:10.1186/s12866-018-1265-x.

Overton BE, Stewart EL, Geiser DM (2006). Taxonomy and phylogenetic relationships of nine species of *Hypocrea* with anamorphs assignable to *Trichoderma* section Hypocreanum. Stud Mycol 56: 39–65. doi:10.3114/sim.2006.56.02

Põldmaa K (2011). Tropical species of *Cladobotryum* and *Hypomyces* producing red pigments. Stud Mycol 68:1–34. doi: 10.3114/sim.2011.68.01

Schoch CL, Seifert KA, Huhndorf S, Robert V, Spouge JL, Levesque CA, Chen NMG, Fungal Barcoding Consortium (2012). Nuclear ribosomal internal transcribed spacer (ITS) region as a universal DNA barcode marker for Fungi. Proc Natl Acad Sci USA 109:6241–6246. doi: 10.1073/pnas.1117018109

Spatafora JW, Sung GH, Sung JM, Hywel-Jones NL, White JF Jr (2007). Phylogenetic evidence for an animal pathogen origin of ergot and the grass endophytes. Mol Ecol 16: 1701–1711. doi: 10.1111/j.1365-294X.2007.03225.x

Taerum SJ, Cafaro MJ, Little AE, Schultz TR, Currie CR (2007). Low host-pathogen specificity in the leaf-cutting ant-microbe symbiosis. Proc Roy Soc B-Biol Sci 274:1971–1978. doi: 10.1098/rspb.2007.043

Taerum SJ, Cafaro MJ, Currie CR (2010) Presence of multiparasite infections within individual colonies of leaf-cutter ants. Environ Entomol 39:105 -113. doi: 10.1603/EN09137

Tamm H, Põldmaa K (2013) Diversity, host associations, and phylogeography of temperate aurofusarin-producing Hypomyces/Cladobotryum including causal agents of cobweb disease of cultivated mushrooms. Fungal Biol 117:348-367. doi: 10.1016/j.funbio.2013.03.005

White TJ, Bruns T, Lee SH, Taylor JW (1990). Amplification and direct sequencing of fungal ribosomal RNA genes for phylogenetics. In: PCR protocols: a guide to methods and applications (Innis MA, Gelfand DH, Sninsky JJ, White TJ). Academic Press, London:315−322. doi: 10.1016/b978-0-12-372180-8.50042-1
