# Supplementary figures and images for: Fungi inhabiting attine ant colonies: reassessment of the genus Escovopsis and description of Luteomyces and Sympodiorosea gens. nov
Source: IMA Fungus. 2021 Aug 24;12:23. doi: 10.1186/s43008-021-00078-8 (PMC8383443; doi:10.1186/s43008-021-00078-8)

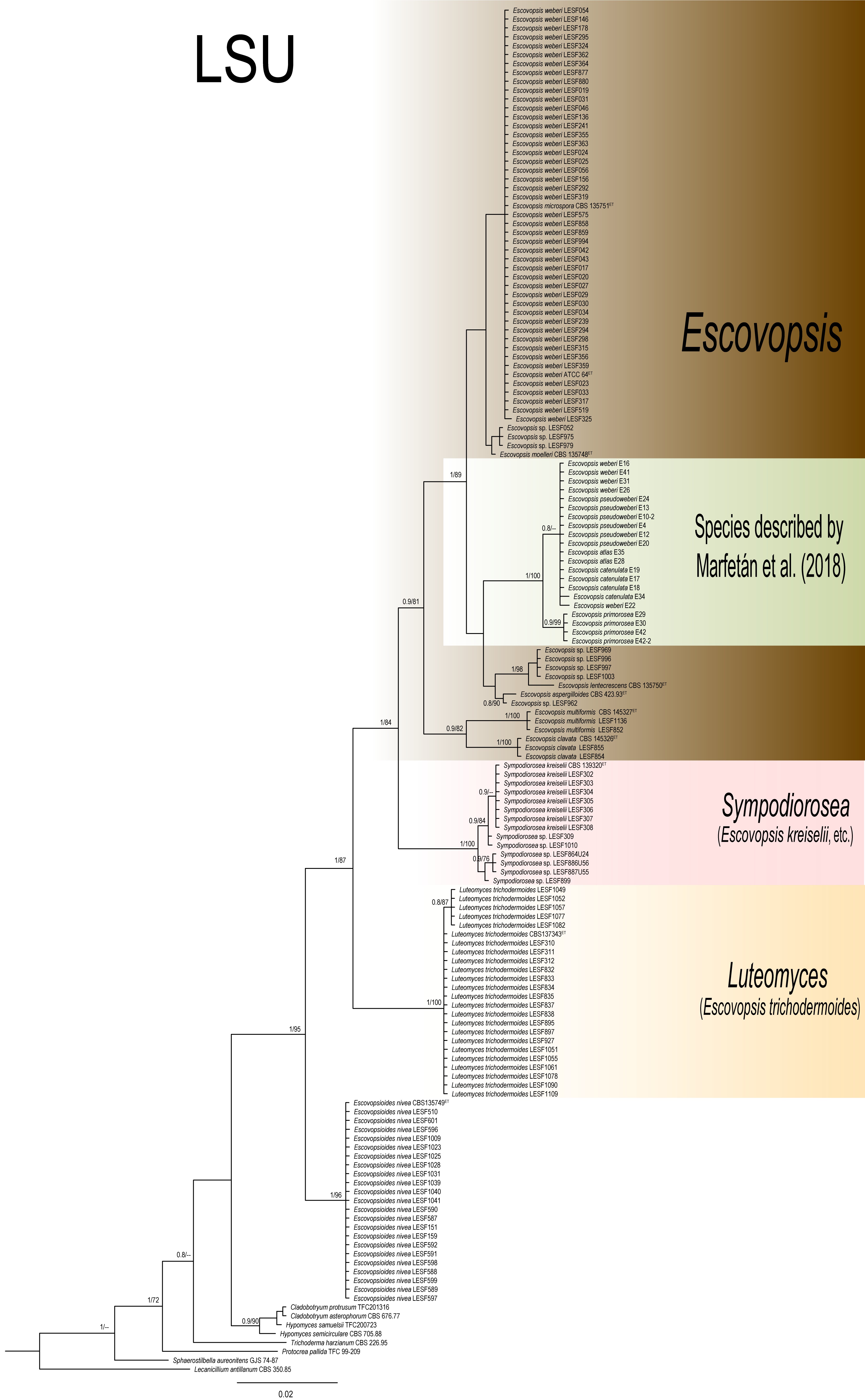

Supplement: Supplementary file 2 — Additional file 2: Figure S1. Phylogenetic placement of Escovopsis species described by Marfetán et al. (2018). The phylogenetic tree was reconstructed to include the LSU sequences (in the green box on the tree) generated by Marfetán et al. (2018). The tree was performed using Maximum Likelihood (ML) in RAxML v.8 (Stamatakis 2014) and Bayesian Inference (BI) in MrBayes v.3.2.2 (Ronquist et al. 2012) using the GTR model. For ML analyses, 1000 independent trees and 1000 bootstrap replicates were generated. For BI analyses, two million generations of the Markov Chain Monte Carlo were enough to reach convergence. Numbers on branches indicate BI posterior probabilities (PP) and ML bootstrap support values (MLB), respectively. Hyphens (--) indicate MLB < 70%. Lecanicillium antillanum CBS 350.85 was used as the outgroup. Four species described by Marfetán et al. (2018) (Escovopsis atlas, E. catenulata, E. pseudoweberi, and E. primorosea) formed two clades (green box) within Escovopsis close to E. aspergilloides and E. lentecrescens. Escovopsis atlas, E. catenulata, E. pseudoweberi were placed into the same clade along with five strains identified as E. weberi (distant from the type of E. weberi) and four strains of E. primorosea formed a monophyletic clade. Escovopsis longivesica was not included in this tree because the LSU sequences of this species do not have similarity with Escovopsis but with Ceriporia alachuana (95.4% identity for E. longivesica E5 and E. longivesica E9) and Penicillium glabrum (95.3% identity for E. longivesica E10). See Additional file 1: Table S4 for all strains and their associated metadata used to infer this phylogenetic tree. [file 43008_2021_78_MOESM2_ESM.jpg]

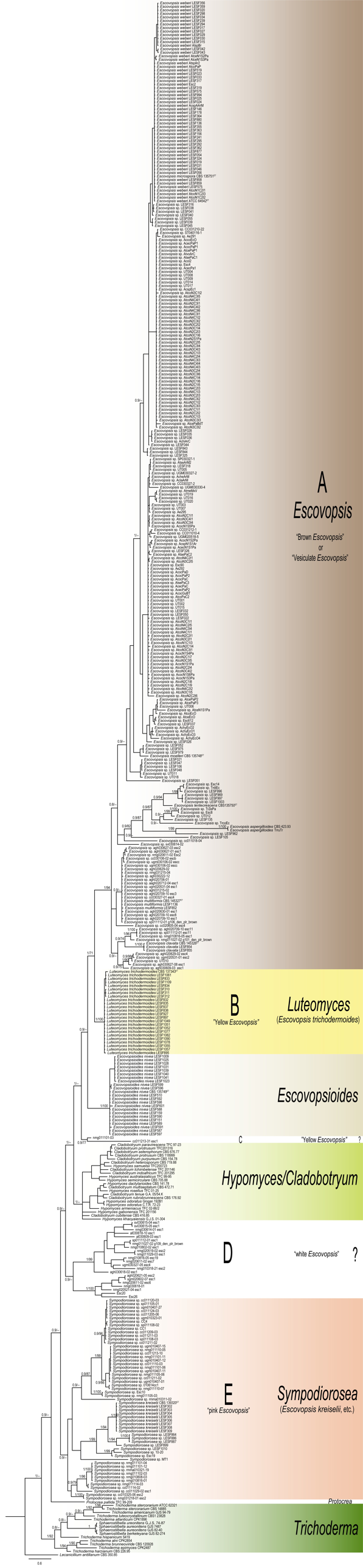

Supplement: Supplementary file 3 — Additional file 3: Figure S2. Extended phylogenetic tree (from Fig. 1) indicating the placement of every isolate previously treated as Escovopsis. The tree shown was inferred using Bayesian Inference (BI). The tree gathers all available tef1 sequences found in the literature and the data set used in this study, including the sequences of the nine Escovopsis ex-type cultures. The tree contains a total of 440 sequences which include: 274 strains of vesiculate-Escovopsis (Clade A), 105 strains of non-vesiculate Escovopsis (Clades B, C, D, E) and 60 strains from four genera, i.e., Escovopsioides, Hypomyces (along with species under its anamorphic genus Cladobotryum), Protocrea, and Trichoderma, in the Hypocreaceae. Lecanicillium antillanum CBS 350.85 was used as the outgroup. Numbers on branches indicate BI posterior probabilities (PP) and Maximum Likelihood bootstrap support values (MLB), respectively. Hyphens (--) indicate MLB < 70%. There is only information, in the literature, on the colour of the colonies of the clades C and D, but the microscopic features of these clades are unknown. See Additional file 1: Table S3 for all strains and their associated metadata used to infer this phylogenetic tree. [file 43008_2021_78_MOESM3_ESM.jpg]
